# Supplementary material for: Synthesis and versatile reactivity of scandium phosphinophosphinidene complexes
Source: Nat Commun. 2020 Jun 9;11:2916. doi: 10.1038/s41467-020-16773-w (PMC7283324; doi:10.1038/s41467-020-16773-w)
Supplement: Supplementary file 1 — Supplementary Information [file 41467_2020_16773_MOESM1_ESM.pdf]

Synthesis and versatile reactivity of scandium phosphinophosphinidene complexes

Feng et al

Supplementary Information

Supplementary Methods........2
1. General........2
2. Synthesis of **2**–**9**........2
3. X-ray crystallography for H_2P\{PN(DIPP)CH_2CH_2N(DIPP)\} and **1**–**9**........8
4. Molecular structures of H_2P\{PN(DIPP)CH_2CH_2N(DIPP)\} and **1**–**9**........15
5. NMR spectra of H_2P\{PN(DIPP)CH_2CH_2N(DIPP)\} and **1**–**9**........20
6. Kinetic study........55
7. Computations........62
Supplementary References........68

Supplementary Methods

General. All operations were carried out under an atmosphere of argon using Schlenk techniques or in a nitrogen filled glovebox. Toluene, tetrahydrofuran, hexane, C_6D_6 and THF-d_8 were dried over Na/K alloy, transferred under vacuum, and stored in the glovebox. ClP{N(DIPP)CH_2CH_2N(DIPP)} (DIPP = 2,6-(^{i}Pr)$_2C_6H_3$) [1,2], $NaPH_2$ [3], [LSc(Me)Cl] (L = [MeC(NDIPP)CHC(Me)(NDIPP)]$^-$) [4], [L'Sc(Me)Cl] (L' = [MeC(NDipp)CHC(Me)$NCH_2CH_2NMe_2$]$^-$) [5], and N-benzylidenepropylamine [6] were synthesized as reported. N-benzylidenepropylamine, phenylacetylene and 1-phenyl-1-propyne were dried over activated 4 Å molecular sieves and degassed by three freeze-pump-thaw cycles before use. Ethylene (99.99%) was further purified by PEN and FA molecular sieves. ^{1}H, ^{13}C{^{1}H}, ^{31}P and ^{31}P{^{1}H} NMR spectra were recorded on a Varian 400 MHz, an Agilent 400 MHz or a Bruker 400 MHz spectrometer. Chemical shifts were reported in δ units with references to the residual solvent resonance of the deuterated solvents for proton and carbon chemical shifts, to external H_3PO_4 (85%) for phosphorus chemical shifts. The assignment of ^{1}H and ^{13}C{^{1}H} resonances was assisted with gCOSY, gHSQC and gHMBC spectra. Elemental analysis was performed by the Analytical Laboratory of Shanghai Institute of Organic Chemistry.

Synthesis of 2. To a THF solution (2 mL) of H_2PP{N(DIPP)CH_2CH_2N(DIPP)} (150 mg, 0.34 mmol) was added $KCH_2(C_6H_5)$ (44 mg, 0.34 mmol) at -35 °C. After standing at -35 °C overnight, to the reaction solution was added a toluene solution (2 mL) of [L'Sc(Me)Cl] (144 mg, 0.34 mmol). After standing at room temperature for 4 h, the solvent was removed under vacuum and the residue was extracted with toluene (6 mL). The solvent of the extraction was removed under vacuum, the residue was washed with hexane (2 × 1 mL) and dried under vacuum to give **2** as a dark red solid (200 mg, 67% yield). ^{1}H NMR (400 MHz, C_6D_6, 25 °C): δ (ppm) 7.36–7.07 (m, Ar*H* of DIPP, overlapped with the residual solvent resonance of the deuterated solvent), 6.99 (m, 1H, Ar*H* of DIPP), 6.85 (m, 1H, Ar*H* of DIPP), 5.23 (s, 1H, MeC(N)C*H*), 4.81 (m, 1H, C*H*Me_2), 4.61 (sept, $^3J_{H\text{-}H}$ = 6.8 Hz, 1H, C*H*Me_2), 4.52 (m, 2H, C*H*Me_2 and NCH_2), 4.29 (m, 2H, C*H*Me_2), 4.17 (m, 1H, NCH_2), 3.91 (m, 1H, NCH_2), 3.79 (m, 1H, NCH_2), 3.55 (m, 1H, NCH_2), 3.04 (br, 4H, THF-*H*), 2.59 (m, 1H, NCH_2), 2.35 (m, 2H, C*H*Me_2 and NCH_2), 1.84 (br, 6H, NMe_2), 1.76 (s, 3H, C*Me*), 1.65 (s, 3H, C*Me*), 1.60 (m, 1H, NCH_2), 1.50 (m, 9H, CHMe_2), 1.41 (m, 13H, THF-*H* and CHMe_2), 1.29 (m, 9H, CHMe_2), 0.98 (m, 3H, CHMe_2),

0.88 (m, 3H, CH*Me*$_2$), 0.77 (d, $^3J_{H\text{-}H}$ = 6.8 Hz, 3H, CH*Me*$_2$). The solubility of **2** in C_6D_6 is low, therefore its $^{13}C\{^1H\}$ NMR spectrum was recorded in THF-d_8. $^{13}C\{^1H\}$ NMR (100 MHz, THF-d_8, 25 °C): δ (ppm) 166.2, 164.4 (imine *C*), 153.6, 153.1 (Ar*C* of DIPP), 150.9 (d, $^2J_{P\text{-}C}$ = 9.5 Hz, *i*-Ar*C* of DIPP), 148.7, 146.0 (Ar*C* of DIPP), 144.2 (dd, $^2J_{P\text{-}C}$ = 11.0 Hz, $^3J_{P\text{-}C}$ = 7.4 Hz, *i*-Ar*C* of DIPP), 143.1, 125.7, 125.6, 125.5, 125.0, 124.9, 124.7, 123.4, 122.2 (Ar*C* of DIPP), 100.7 (MeC(N)*C*H), 68.0 (THF-*C*), 60.1 (d, $^3J_{P\text{-}C}$ = 15.7 Hz, N*C*H$_2$), 54.7 (d, $^2J_{P\text{-}C}$ = 8.7 Hz, N*C*H$_2$), 53.5 (d, $^2J_{P\text{-}C}$ = 7.3 Hz, N*C*H$_2$), 46.7 (N*C*H$_2$), 46.4 (br, N*Me*$_2$), 29.7, 29.6, 29.5, 29.2, 28.8, 27.8 (*C*HMe$_2$), 28.3, 28.2, 27.3, 26.8, 26.7, 26.1, 24.1, 23.7, 22.7, 22.0 (CH*Me*$_2$), 26.2 (THF-*C*), 25.7, 22.9 (*CMe*). $^{31}P\{^1H\}$ NMR (162 MHz, C_6D_6, 25 °C): δ (ppm) 324.8 (d, J = 518.0 Hz, P_α), 169.0 (d, J = 518.0 Hz, P_β). $^{31}P\{^1H\}$ NMR (162 MHz, THF-d_8, 25 °C): δ (ppm) 312.2 (d, $^1J_{P\text{-}P}$= 518.0 Hz, P_α), 166.8 (d, $^1J_{P\text{-}P}$ = 518.0 Hz, P_β). Complex **2** decomposes when it is dried under vacuum, this is possibly associated with the loss of the coordinated THF under vacuum. The XRD study reveals the distance from Sc ion to oxygen of THF in **2** is 0.08 Å longer than that in **1**. When the THF is lost, the complex becomes instable. Therefore, the complex can't be dried under vacuum for a long time, hexane and toluene can't be completely removed, and a satisfied elemental analysis for **2** was not obtained. For the NMR spectra of **2**, see Supplementary Figures 20-22.

Synthesis of 3. N-benzylidenepropylamine (16 mg, 0.106 mmol) was added to the toluene solution (3 mL) of **1**·hexane (106 mg, 0.10 mmol). After standing at room temperature for 12 h, the reaction solution was concentrated to approximately 0.5 mL, then 2 mL of hexamethyl disiloxane ($Me_3SiOSiMe_3$) was added. **3**·2$Me_3SiOSiMe_3$ was isolated as an orange crystalline solid after this solution was allowed to stand at -35 °C overnight (107 mg, 78% yield) (complex **3** was obtained with hexamethyl disiloxane in the lattice). ^1H NMR (400 MHz, C_6D_6, 25 °C): δ (ppm) 7.37–7.13 (m, Ar*H* of DIPP, overlapped with the residual solvent resonance of the deuterated solvent), 6.99 (m, 3H, Ar*H* of DIPP), 6.91 (m, 1H, Ar*H* of DIPP), 6.85 (m, 1H, Ph*H*), 6.75 (m, 2H, Ph*H*), 5.84 (d, $^3J_{H\text{-}H}$ = 6.0 Hz, 2H, Ph*H*), 4.97 (s, 1H, MeC(N)C*H*), 4.34 (d, $^2J_{P\text{-}H}$ = 5.6 Hz, 1H, PC*H*Ph), 4.15 (m, 2H, C*H*Me$_2$), 3.95 (m, 1H, NC*H*$_2$), 3.90 (m, 1H, C*H*Me$_2$), 3.68 (m, 1H, NC*H*$_2$), 3.56 (m, 1H, C*H*Me$_2$), 3.45 (m, 2H, NC*H*$_2$ and C*H*Me$_2$), 3.23 (m, 1H, C*H*Me$_2$), 3.11 (m, 3H, NC*H*$_2$, C*H*Me$_2$ and NC*H*$_2$CH$_2$CH$_3$), 2.26 (m, 1H, C*H*Me$_2$), 2.10 (m, 1H, NC*H*$_2$CH$_2$CH$_3$), 1.86 (d, $^3J_{H\text{-}H}$ = 5.6 Hz, 3H, CH*Me*$_2$), 1.59 (m, 15H, C*Me* and CH*Me*$_2$), 1.43 (d, $^3J_{H\text{-}H}$ = 6.8 Hz, 3H, CH*Me*$_2$), 1.39 (d, $^3J_{H\text{-}H}$ = 6.0 Hz, 3H, CH*Me*$_2$), 1.28 (m, 15H,

CHMe_2), 1.11 (d, $^3J_{H\text{-}H}$ = 6.4 Hz, 3H, CHMe_2), 1.07 (d, $^3J_{H\text{-}H}$ = 6.4 Hz, 3H, CHMe_2), 0.99 (m, 1H, $NCH_2CH_2CH_3$), 0.90 (d, $^3J_{H\text{-}H}$ = 6.0 Hz, 3H, CHMe_2), 0.69 (d, $^3J_{H\text{-}H}$ = 6.0 Hz, 3H, CHMe_2), 0.34 (d, $^3J_{H\text{-}H}$ = 6.0 Hz, 3H, CHMe_2), 0.23 (m, 4H, $NCH_2CH_2CH_3$ and $NCH_2CH_2CH_3$), 0.12 (s, 36H, $SiMe_3$). $^{13}C\{^1H\}$ NMR (100 MHz, C_6D_6, 25 °C): δ (ppm) 170.0, 166.9 (imine *C*), 150.6 (d, $^2J_{P\text{-}C}$ = 8.9 Hz, *i*-Ph*C*), 149.9, 149.4, 148.3, 143.8, 142.9, 142.8, 142.7, 142.4, 141.5 (Ar*C* of DIPP), 138.9 (d, $^2J_{P\text{-}C}$ = 9.6 Hz, *i*-Ar*C* of DIPP), 128.1 (*o*-Ph*C*), 127.2 (*m*-Ph*C*), 127.1, 126.1, 126.0, 125.5, 124.8, 124.5, 124.2, 123.9, 123.86, 123.8 (Ar*C* of DIPP), 123.7 (*p*-Ph*C*), 98.7 (MeC(N)*C*H), 57.2 (NCH_2), 55.5 (d, $^2J_{P\text{-}C}$ = 7.6 Hz, NCH_2), 54.4 ($NCH_2CH_2CH_3$), 52.8 (dd, $^1J_{P\text{-}C}$ = 44.0 Hz, $^2J_{P\text{-}C}$ = 40.3 Hz, P*C*HPh), 32.4, 29.5, 29.0, 28.92, 28.86, 28.69, 28.65, 28.5 (*C*HMe_2), 27.2, 27.0, 26.8, 26.3, 25.7, 25.6, 25.3, 25.1, 25.0, 24.9, 24.8, 24.4, 24.3, 24.2, 24.1, 23.7, 23.66, 23.3 (*C*HMe_2 and C*Me*), 21.8 ($NCH_2CH_2CH_3$), 11.3 ($NCH_2CH_2CH_3$), 2.09 (SiMe_3). $^{31}P\{^1H\}$ NMR (162 MHz, C_6D_6, 25 °C): δ (ppm) 169.0 (d, $^1J_{P\text{-}P}$ = 383.6 Hz, P_β), 40.8 (d, $^1J_{P\text{-}P}$ = 383.6 Hz, P_α). Anal. Calcd for $C_{65}H_{92}N_5P_2Sc\cdot 2Me_3SiOSiMe_3$: C 67.25; H 9.38; N 5.09. Found: C 66.63; H 9.74; N 4.99.

Synthesis of 4. 2,2'-Bipyridine (16 mg, 0.10 mmol) was added to the toluene solution (4 mL) of **1**·hexane (106 mg, 0.10 mmol). After standing at room temperature for 0.5 h, the volatiles were removed under vacuum to give **4** as a dark blue solid (106 mg, 97% yield). ^1H NMR (400 MHz, C_6D_6, 25 °C): δ (ppm) 8.58 (d, $^3J_{H\text{-}H}$ = 5.6 Hz, 1H, py-*H*), 7.29–7.07 (m, Ar*H* of DIPP and py-*H*, overlapped with the residual solvent resonance of the deuterated solvent), 7.01 (m, 1H, Ar*H* of DIPP), 6.97 (m, 1H, Ar*H* of DIPP), 6.90 (m, 1H, Ar*H* of DIPP), 6.83 (m, 1H, py-*H*), 6.40 (m, 1H, py-*H*), 6.11 (m, 1H, NCH(P)CHC*H*CH), 5.87 (m, 2H, NC*H*(P)CHCHCH and NCH(P)C*H*CHCH), 5.26 (d, $^3J_{H\text{-}H}$ = 6.2 Hz, 1H, NCH(P)CHCHC*H*), 4.95 (s, 1H, MeC(N)C*H*), 4.76 (sept, $^3J_{H\text{-}H}$ = 6.8 Hz, 1H, C*H*Me_2), 3.90 (m, 1H, NCH_2), 3.82 (m, 2H, C*H*Me_2), 3.58 (m, 3H, C*H*Me_2 and NCH_2), 3.40 (sept, $^3J_{H\text{-}H}$ = 6.8 Hz, 1H, C*H*Me_2), 3.23 (m, 2H, NCH_2), 2.50 (sept, $^3J_{H\text{-}H}$ = 6.8 Hz, 1H, C*H*Me_2), 2.21 (sept, $^3J_{H\text{-}H}$ = 6.8 Hz, 1H, C*H*Me_2), 1.56 (d, $^3J_{H\text{-}H}$ = 6.8 Hz, 3H, CHMe_2), 1.53 (s, 3H, C*Me*), 1.47 (d, $^3J_{H\text{-}H}$ = 6.8 Hz, 3H, CHMe_2), 1.37 (s, 3H, C*Me*), 1.32 (d, $^3J_{H\text{-}H}$ = 6.8 Hz, 3H, CHMe_2), 1.25–1.15 (m, 24H, CHMe_2), 1.10 (d, $^3J_{H\text{-}H}$ = 6.8 Hz, 3H, CHMe_2), 1.08 (d, $^3J_{H\text{-}H}$ = 6.8 Hz, 3H, CHMe_2), 0.87 (d, $^3J_{H\text{-}H}$ = 6.8 Hz, 3H, CHMe_2), 0.70 (d, $^3J_{H\text{-}H}$ = 6.8 Hz, 3H, CHMe_2), 0.34 (d, $^3J_{H\text{-}H}$ = 6.8 Hz, 3H, CHMe_2). $^{13}C\{^1H\}$ NMR (100 MHz, C_6D_6, 25 °C): δ (ppm) 168.5, 168.4 (imine *C*), 158.8, 151.4, 150.4, 150.2, 150.0, 149.7, 149.5, 145.5 143.6, 143.2, 143.0, 142.4, 141.51, 141.45, 138.4 (Ar*C* of DIPP, py-*C* and NCH(P)CHCHCH*C*), 145.2, 136.8

(py-*C*H), 127.1, 126.9, 126.6, 126.0, 125.1, 125.0, 124.9, 124.8, 123.8, 123.6, 123.5, 123.2 (Ar*C* of DIPP), 125.3 (d, $^{3}J_{P\text{-}C}$ = 7.8 Hz, NCH(P)CH*C*HCH), 120.2, 119.9 (py-*C*H), 117.2 (d, $^{2}J_{P\text{-}C}$ = 8.5 Hz, NCH(P)*C*HCHCH), 98.5 (MeC(N)*C*H), 95.3 (NCH(P)CHCH*C*H), 78.3 (dd, $^{1}J_{P\text{-}C}$ = 77.7 Hz, $^{2}J_{P\text{-}C}$ = 9.3 Hz, NC*H*(P)CHCHCH), 52.9 (N*C*H_2), 51.4 (d, $^{2}J_{P\text{-}C}$ = 4.3 Hz, N*C*H_2), 29.5, 29.4, 29.2, 29.1, 28.9, 28.6, 28.5, 27.5 (*C*HMe_2), 27.9, 27.3, 27.2, 27.1, 25.8, 25.7, 25.4, 25.34, 25.30, 25.2, 25.1, 25.07, 24.99, 24.90, 24.8, 24.3, 23.9, 23.7 (CHMe_2 and C*Me*). ^{31}P{^{1}H} NMR (162 MHz, C_6D_6, 25 °C): δ (ppm) 110.0 (d, $^{1}J_{P\text{-}P}$ = 639.4 Hz, P_β), -64.4 (d, $^{1}J_{P\text{-}P}$ = 639.4 Hz, P_α). Anal. Calcd for $C_{65}H_{87}N_6P_2Sc$: C 73.70; H 8.28; N 7.93. Found: C 73.46; H 8.58; N 7.86.

Synthesis of 5. Ethylene (15 mL, 0.61 mmol) was injected into a 15 mL flask containing a toluene solution (4 mL) of **1**·hexane (106 mg, 0.10 mmol) at room temperature. After 15 minutes, the volatiles of the reaction solution were removed under vacuum to give a yellow solid. The solid was washed with hexane (1 mL) and dried under vacuum to give **5** as a yellow solid (88 mg, 92% yield). ^{1}H NMR (400 MHz, C_6D_6 , 25 °C): δ (ppm) 7.23 (m, 6H, Ar*H* of DIPP), 7.06 (m, 6H, Ar*H* of DIPP), 4.71 (s, 1H, MeC(N)C*H*), 4.00 (sept, $^{3}J_{H\text{-}H}$ = 6.8 Hz, 2H, C*H*Me_2), 3.49 (sept, $^{3}J_{H\text{-}H}$ = 6.8 Hz, 2H, C*H*Me_2), 3.39 (m, 2H, NCH_2), 3.31 (m, 2H, NCH_2), 3.10 (sept, $^{3}J_{H\text{-}H}$ = 6.8 Hz, 4H, C*H*Me_2), 2.68 (m, 2H, PCH_2), 1.63 (d, $^{3}J_{H\text{-}H}$ = 6.8 Hz, 6H, CHMe_2), 1.43 (s, 6H, C*Me*), 1.25 (d, $^{3}J_{H\text{-}H}$ = 6.8 Hz, 6H, CHMe_2), 1.15 (d, $^{3}J_{H\text{-}H}$ = 6.8 Hz, 6H, CHMe_2), 1.11 (br, 12H, CHMe_2), 1.01 (d, $^{3}J_{H\text{-}H}$ = 6.8 Hz, 12H, CHMe_2), 0.94 (d, $^{3}J_{H\text{-}H}$ = 6.8 Hz, 6H, CHMe_2), 0.82 (m, 1H, ScCH_2), 0.73 (m, 1H, ScCH_2). ^{13}C{^{1}H} NMR (100 MHz, C_6D_6): δ (ppm) 167.9 (imine *C*), 151.0, 150.2, 144.1, 139.7, 139.1 , 127.4, 127.2, 124.9, 124.5, 123.7 (Ar*C* of DIPP), 98.4 (MeC(N)*C*H), 51.3 (d, $^{2}J_{P\text{-}C}$ = 4.6 Hz, N*C*H_2), 48.7 (m, Sc*C*H_2), 39.9 (dd, $^{1}J_{P\text{-}C}$ = 61.1 Hz, $^{2}J_{P\text{-}C}$ = 9.7 Hz, P*C*H_2), 29.3, 29.0, 28.4 (*C*HMe_2), 27.7, 27.2, 25.1, 24.9, 24.3, 24.1 (CHMe_2), 23.9 (C*Me*). ^{1}P{^{1}H} NMR (162 MHz, C_6D_6, 25 °C): δ (ppm) 90.4 (d, $^{1}J_{P\text{-}P}$ = 634.0 Hz, P_β), -61.3 (d, $^{1}J_{P\text{-}P}$ = 634.0 Hz, P_α). Anal. Calcd for $C_{57}H_{83}N_4P_2Sc$: C 73.52; H 8.98; N 6.02. Found: C 73.30; H 9.27; N 5.87.

Synthesis of 6. Phenylacetylene (11 mg, 0.10 mmol) was added to the toluene solution (2 mL) of **1**·hexane (106 mg, 0.10 mmol). After standing at room temperature for 2 h, the volatiles of the reaction solution were removed under vacuum, the residue was washed with hexane (2 × 1 mL) and dried under vacuum to give **6** as an orange solid (86 mg, 84% yield). ^{1}H NMR (400 MHz, C_6D_6, 25 °C): δ (ppm)

7.88 (d, $^2J_{P-H}$ = 15.0 Hz, 1H, PhC=C(P)*H*), 7.45 (d, $^3J_{H-H}$ = 7.2 Hz, 2H, Ar*H* of DIPP or Ph*H*), 7.31–7.10 (m, Ar*H* of DIPP or Ph*H*, overlapped with the residual solvent resonance of the deuterated solvent), 7.08–6.98 (m, 5H, Ar*H* of DIPP or Ph*H*), 6.92–6.81 (m, 2H, Ar*H* of DIPP or Ph*H*), 4.91 (s, 1H, MeC(N)C*H*), 4.27–4.08 (m, 4H, C*H*Me$_2$), 3.57 (m, 2H, NC*H*$_2$), 3.46 (m, 2H, NC*H*$_2$), 3.36 (sept, $^3J_{H-H}$ = 6.8 Hz, 2H, C*H*Me$_2$), 2.72 (sept, $^3J_{H-H}$ = 6.8 Hz, 2H, C*H*Me$_2$), 1.64 (d, $^3J_{H-H}$ = 6.8 Hz, 6H, CH*Me*$_2$), 1.42 (d, $^3J_{H-H}$ = 6.8 Hz, 6H, CH*Me*$_2$), 1.40 (s, 6H, C*Me*), 1.28 (d, $^3J_{H-H}$ = 6.8 Hz, 6H, CH*Me*$_2$), 1.16 (d, $^3J_{H-H}$ = 6.8 Hz, 6H, CH*Me*$_2$), 1.10 (d, $^3J_{H-H}$ = 6.8 Hz, 6H, CH*Me*$_2$), 0.81 (d, $^3J_{H-H}$ = 6.8 Hz, 12H, CH*Me*$_2$), 0.20 (d, $^3J_{H-H}$ = 6.8 Hz, 6H, CH*Me*$_2$). ^{13}C{^{1}H} NMR (100 MHz, C_6D_6, 25 °C): δ (ppm) 168.8 (imine *C*), 152.3, 149.8 (Ar*C* of DIPP), 145.0 (d, $^2J_{P-C}$ = 38.1 Hz, Ph*C*=C(P)H), 143.3, 142.6, 141.3 (Ar*C* of DIPP), 137.8 (dd, $^1J_{P-C}$ = 93.1 Hz, $^2J_{P-C}$ = 11.4 Hz, PhC=*C*(P)H), 137.7 (d, $^3J_{P-C}$ = 5.2 Hz, *i*-Ph*C*), 127.5, 126.9, 125.1, 124.5, 124.4, 123.7 (Ar*C* of DIPP and Ph*C*), 97.7 (MeC(N)*C*H), 52.1 (d, $^2J_{P-C}$ = 7.2 Hz, N*C*H$_2$), 29.6, 29.5, 29.4, 29.2, 28.4 (*C*HMe$_2$), 27.15, 27.13, 25.3, 25.26, 24.8, 24.7, 24.4, 24.2 (CH*Me*$_2$), 24.1 (C*Me*). ^{31}P{^{1}H} NMR (162 MHz, C_6D_6, 25 °C): δ (ppm) 69.0 (d, $^1J_{P-P}$ = 614.8 Hz, P_β), 24.9 (d, $^1J_{P-P}$ = 614.8 Hz, P_α). Anal. Calcd for $C_{63}H_{85}N_4P_2Sc$: C 75.27; H 8.52; N 5.57. Found: C 75.36; H 8.79; N 5.22.

Synthesis of 7. 1-Phenyl-1-propyne (17 mg, 0.15 mmol) was added to the toluene solution (3 mL) of **1**·hexane (159 mg, 0.15 mmol). After standing at room temperature for 8 h, the volatiles of the reaction solution were removed under vacuum, the residue was extracted with hexane (4 mL). The resulting solution is concentrated to 1 mL to give a yellow crystalline solid. The solid was washed with hexane (3 × 0.5 mL) and dried under vacuum to give **7** as an orange solid (51mg, 34% yield). ^{1}H NMR (400 MHz, C_6D_6, 25 °C): δ (ppm) 7.31–7.07 (m, Ar*H* of DIPP and Ph*H*, overlapped with the residual solvent resonance of the deuterated solvent), 7.01 (m, 2H, Ar*H* of DIPP or Ph*H*), 6.95 (t, $^3J_{H-H}$ = 7.6 Hz, 1H, Ar*H* of DIPP or Ph*H*), 6.48 (d, $^3J_{H-H}$ = 7.6 Hz, 2H, Ar*H* of DIPP or Ph*H*), 4.77 (s, 1H, MeC(N)C*H*), 3.78 (sept, $^3J_{H-H}$ = 6.8 Hz, 2H, C*H*Me$_2$), 3.64 (sept, $^3J_{H-H}$ = 6.8 Hz, 2H, C*H*Me$_2$), 3.48 (m, 2H, NC*H*$_2$), 3.38 (m, 4H, NC*H*$_2$ and C*H*Me$_2$), 2.42 (sept, $^3J_{H-H}$ = 6.8 Hz, 2H, C*H*Me$_2$), 2.07 (d, $^3J_{P-H}$ = 9.8 Hz, 3H, PhC=C(P)*Me*), 1.58 (d, $^3J_{H-H}$ = 6.8 Hz, 6H, CH*Me*$_2$), 1.45 (s, 6H, C*Me*), 1.27 (d, $^3J_{H-H}$ = 6.8 Hz, 6H, CH*Me*$_2$), 1.18 (d, $^3J_{H-H}$ = 6.8 Hz, 6H, CH*Me*$_2$), 1.11 (d, $^3J_{H-H}$ = 6.8 Hz, 6H, CH*Me*$_2$), 0.99 (d, $^3J_{H-H}$ = 6.8 Hz, 6H, CH*Me*$_2$), 0.89 (d, $^3J_{H-H}$ = 6.8 Hz, 6H, CH*Me*$_2$), 0.78 (d, $^3J_{H-H}$ = 6.8 Hz, 6H, CH*Me*$_2$), 0.74 (d, $^3J_{H-H}$ = 6.8 Hz, 6H, CH*Me*$_2$). ^{13}C{^{1}H} NMR (100 MHz, C_6D_6, 25 °C): δ (ppm) 168.1 (imine *C*),

150.7, 150.1 (Ar*C* of DIPP), 149.3 (d, $^1J_{P\text{-}C}$ = 41.3 Hz, PhC=*C*(P)Me), 145.1, 142.1, 141.6 (Ar*C* of DIPP), 140.6 (d, $^3J_{P\text{-}C}$ = 5.7 Hz, *i*-Ph*C*), 126.8, 125.9, 125.2, 124.1, 124.0, 123.8 (Ar*C* of DIPP and Ph*C*), 98.1 (MeC(N)*C*H), 53.3 (d, $^2J_{P\text{-}C}$ = 3.7 Hz, N*C*H_2), 29.2, 28.9, 28.5, 27.8 (*C*HMe_2), 26.7, 26.6, 26.0, 25.9, 25.6, 25.55, 25.1, 24.8, 24.7, 23.6 (CH*Me*$_2$), 24.3 (C*Me*), 19.3 (d, $^2J_{P\text{-}C}$ = 12.7 Hz, PhC=C(P)*Me*), the signal of Ph*C*=C(P)Me was not observed. ^{31}P{^{1}H} NMR (162 MHz, C_6D_6, 25 °C): δ (ppm) 80.3 (d, $^1J_{P\text{-}P}$ = 646.6 Hz, P_β), -18.6 (d, $^1J_{P\text{-}P}$ = 646.6 Hz, P_α). Anal. Calcd for $C_{64}H_{87}N_4P_2Sc$: C 75.41; H 8.60; N 5.50. Found: C 75.35; H 8.90; N 5.22.

Synthesis of 8. Phenylacetylene (12 mg, 0.113 mmol) was added to the toluene solution (2 mL) of **2** (100 mg, 0.113 mmol). After standing at room temperature for 0.5 h, the volatiles of the reaction solution were removed under vacuum, the residue was washed with hexane (1 mL) and dried under vacuum to give **8** as a yellow solid (78 mg, 76% yield). ^{1}H NMR (400 MHz, C_6D_6, 25 °C): δ (ppm) 7.29–7.10 (m, Ar*H* of DIPP and Ph*H*, overlapped with the residual solvent resonance of the deuterated solvent), 7.09–7.00 (m, 3H, Ar*H* of DIPP and Ph*H*), 6.92 (m, 1H, Ar*H* of DIPP or Ph*H*), 4.81 (s, 1H, MeC(N)C*H*), 4.36 (m, 1H, C*H*Me_2), 4.23 (m, 1H, C*H*Me_2), 4.14 (m, 1H, NCH_2), 3.96 (m, 1H, NCH_2), 3.85 (m, 2H, C*H*Me_2), 3.77 (m, 1H, C*H*Me_2), 3.25 (m, 4H, NCH_2), 3.01 (overlapped with other signals, 1H, P*H*), 2.77 (m, 2H, NCH_2 and C*H*Me_2), 2.20 (s, 3H, N*Me*$_2$), 2.07 (s, 3H, N*Me*$_2$), 1.78 (m, 4H, CH*Me*$_2$ and NCH_2), 1.70 (d, $^3J_{H\text{-}H}$ = 6.8 Hz, 3H, CH*Me*$_2$), 1.58 (m, 6H, C*Me* and CH*Me*$_2$), 1.40 (m, 12H, CH*Me*$_2$), 1.29 (m, 23H, C*Me*, CH*Me*$_2$ and hexane-*H*), 1.01 (d, $^3J_{H\text{-}H}$ = 6.8 Hz, 3H, CH*Me*$_2$). ^{13}C{^{1}H} NMR (100 MHz, C_6D_6, 25 °C): δ (ppm) 166.7, 166.5 (imine *C*), 151.3, 149.2, 148.8, 145.1, 144.1, 141.6, 139.5, 132.3, 127.0, 126.9, 126.3, 126.1, 125.3, 124.8, 124.6, 123.7, 123.5, 123.2, 105.0 (Ar*C* of DIPP, Ph*C* and Sc<u>C</u>CPh), 100.1 (MeC(N)*C*H), 99.0 (ScC*C*Ph), 59.1 (d, $^3J_{P\text{-}C}$ = 16.8 Hz, N*C*H_2), 54.8 (d, $^2J_{P\text{-}C}$ = 7.8 Hz, N*C*H_2), 54.0 (d, $^2J_{P\text{-}C}$ = 8.1 Hz, N*C*H_2), 47.4 (N*C*H_2), 46.3 (N*Me*$_2$), 43.5 (N*Me*$_2$), 29.5, 29.2, 29.1, 28.9, 28.8, 28.5 (*C*HMe_2), 26.8, 26.4, 26.3, 26.2, 25.9, 25.8, 25.1, 24.8, 24.5, 24.2, 24.0, 23.5, 22.8 (CH*Me*$_2$ and C*Me*), 32.0, 23.0, 14.4 (hexane-*C*). ^{31}P{^{1}H} NMR (162 MHz, C_6D_6, 25 °C): δ (ppm) 156.7 (d, $^1J_{P\text{-}P}$ = 285.4 Hz, P_β), -41.0 (d, $^1J_{P\text{-}P}$ = 285.4 Hz, P_α). ^{31}P NMR (162 MHz, C_6D_6, 25 °C): δ (ppm) 156.7 (d, $^1J_{P\text{-}P}$ = 285.4 Hz, P_β), -41.0 (dd, $^1J_{P\text{-}P}$ = 285.4 Hz, $^1J_{P\text{-}H}$ = 141.4 Hz, P_α). Anal. Calcd for $C_{55}H_{78}N_5P_2Sc$·hexane: C 73.10; H 9.29; N 6.99. Found: C 72.73; H 8.91; N 6.79.

Synthesis of 9. 1-Phenyl-1-propyne (13 mg, 0.11 mmol) was added to a toluene solution (2 mL) of **2**

(100 mg, 0.11 mmol). After standing at room temperature for 8 h, the volatiles of the reaction solution were removed under vacuum, the residue was washed with hexane (1 mL) and dried under vacuum to give **9** as a yellow solid (81 mg, 79% yield). ^{1}H NMR (400 MHz, C_6D_6, 25 °C): δ (ppm) 7.18 (m, Ar*H* of DIPP and Ph*H*, overlapped with the residual solvent resonance of the deuterated solvent), 7.08 (m, 3H, Ar*H* of DIPP and Ph*H*), 6.98 (m, 3H, Ar*H* of DIPP and Ph*H*), 6.84 (t, $^{3}J_{H\text{-}H}$ = 7.2 Hz, 1H, Ar*H* of DIPP or Ph*H*), 5.79 (d, $^{3}J_{H\text{-}H}$ = 7.2 Hz, 2H, Ph*H*), 4.92 (s, 1H, MeC(N)C*H*), 4.40 (m, 1H, NCH_2), 4.30 (sept, $^{3}J_{H\text{-}H}$ = 6.8 Hz, 1H, C*H*Me_2), 4.00 (m, 3H, C*H*Me_2), 3.76 (m, 1H, C*H*Me_2), 3.68 (m, 1H, NCH_2), 3.36 (m, 2H, NCH_2), 2.73 (sept, $^{3}J_{H\text{-}H}$ = 6.8 Hz, 1H, C*H*Me_2), 2.55 (m, 1H, NCH_2), 2.38 (m, 4H, NCH_2 and NMe_2), 2.08 (m, 1H, NCH_2), 1.94 (d, $^{3}J_{P\text{-}H}$ = 4.4 Hz, 3H, PhC=C(P)*Me*), 1.82 (s, 3H, NMe_2), 1.68 (s, 3H, C*Me*), 1.48 (m, 19H, NCH_2, C*Me* and CHMe_2), 1.38 (d, $^{3}J_{H\text{-}H}$ = 6.8 Hz, 3H, CHMe_2), 1.33 (m, 9H, CHMe_2), 1.21 (d, $^{3}J_{H\text{-}H}$ = 6.8 Hz, 3H, CHMe_2), 0.99 (d, $^{3}J_{H\text{-}H}$ = 6.8 Hz, 3H, CHMe_2), 0.84 (d, $^{3}J_{H\text{-}H}$ = 6.8 Hz, 3H, CHMe_2). ^{13}C{^{1}H} NMR (100 MHz, C_6D_6, 25 °C): δ (ppm) 213.1 ($^{2}J_{P\text{-}C}$ = 18.0 Hz, Ph*C*=C(P)Me), 166.7, 166.4 (imine *C*), 151.6 (Ar*C* of DIPP), 150.1 (d, $^{3}J_{P\text{-}C}$ = 6.0 Hz, Ph*C*), 149.9, 149.3, 147.1 (Ar*C* of DIPP), 145.2 (dd, $^{1}J_{P\text{-}C}$ = 52.6, $^{2}J_{P\text{-}C}$ = 13.2 Hz, PhC=*C*(P)Me), 143.5, 141.7, 141.7, 140.6, 127.4, 126.6, 126.4, 125.6, 124.9, 124.9, 124.7, 124.2, 124.0, 123.6, 121.7 (Ar*C* of DIPP and Ph*C*), 100.0 (MeC(N)*C*H), 58.3 (N*C*H_2), 55.0 (d, $^{2}J_{P\text{-}C}$ = 5.7 Hz, N*C*H_2), 54.5 (d, $^{2}J_{P\text{-}C}$ = 7.2 Hz, N*C*H_2), 49.5 (d, $^{3}J_{P\text{-}C}$ = 21.6 Hz, NMe_2), 47.0 (N*C*H_2), 41.3 (NMe_2), 29.4, 29.12, 29.05, 28.93, 28.89, 28.8, 28.6 (CHMe_2 and PhC=C(P)*Me*), 28.3, 27.4, 26.5, 25.9, 25.2, 24.9, 24.8, 24.6, 24.5, 24.4, 24.3, 23.4, 22.9 (CHMe_2 and C*Me*). ^{31}P{^{1}H} NMR (162 MHz, C_6D_6, 25 °C): δ (ppm) 173.5 (d, J = 361.4 Hz, P_β), 70.6 (d, J = 361.4 Hz, P_α). Anal. Calcd for $C_{56}H_{80}N_5P_2Sc$: C 72.31; H 8.67; N 7.53. Found: C 72.06; H 8.78; N 7.57.

X-ray Crystallography. Single crystals of **1**·hexane, **2** and **4**–**9** suitable for single-crystal X-ray diffraction were grown a mixture solution of toluene and hexane, those of H_2PP{N(DIPP)CH_2CH_2N(DIPP)} were grown from hexane solution, and those of **3**·2Me_3SiOSiMe_3 were grown from a mixture solution of hexane and hexamethyl disiloxane. The single crystals of H_2PP{N(DIPP)CH_2CH_2N(DIPP)}, **1**·hexane, **2**, **3**·2Me_3SiOSiMe_3 and **4**–**9** were mounted under nitrogen atmosphere on a glass fiber at low temperature. Data collections of H_2PP{N(DIPP)CH_2CH_2N(DIPP)}, **1**·hexane, **2**, **3**·2Me_3SiOSiMe_3, **4**, **5** and **7**–**9** were performed on a Bruker D8 Venture with Ga Kα radiation (λ = 1.34139 Å), that of **6** was performed on a Bruker APEX-

II CCD with Ga Kα radiation (λ = 1.34139 Å). The SMART program package was used to determine the unit cell parameters. The absorption correction was applied using SADABS program[7]. All structures were solved by direct methods and refined on F^2 by full-matrix least-squares techniques with anisotropic thermal parameters for non-hydrogen atoms. Hydrogen atoms were placed at calculated positions and were included in the structure calculation, except for the hydrogen atoms on the P_α in $H_2PP\{N(DIPP)CH_2CH_2N(DIPP)\}$ and **8**, which were located from the Fourier map. Calculations were carried out using the SHELXL-97, SHELXL-2014 or Olex2 program[8-13]. Crystallographic data and refinement for $H_2PP\{N(DIPP)CH_2CH_2N(DIPP)\}$, **1**·hexane, **2**, **3**·$2Me_3SiOSiMe_3$ and **4**–**9** are listed in Supplementary Table 1.

Supplementary Table 1. Crystallographic Data and Refinement for $H_2PP\{N(DIPP)CH_2CH_2N(DIPP)\}$ and **1**–**9**.

	$H_2PP\{N(DIPP)CH_2CH_2N(DIPP)\}$	**1**·hexane
formula	$C_{26}H_{40}N_2P_2$	$C_{65}H_{101}N_4OP_2Sc$
fw	442.59	1061.39
color	colorless	red
cryst syst.	orthorhombic	monoclinic
space group	$P2_12_12_1$	$P2_1/c$
a, Å	9.112(1)	14.289(1)
b, Å	9.879(1)	21.347(1)
c, Å	28.376(1)	20.411(1)
α, deg	90	90
β, deg	90	90.32(1)
γ, deg	90	90
V, Å^3	2554.4(1)	6225.8(3)
Z	4	4
D_{calcd}, (g/cm^3)	1.151	1.132
F(000)	960	2312
T(K)	170	170
θ range (deg)	2.709 to 54.953	3.603 to 57.212
no. of refns collected	24814	59373
no. of unique refns	4835	12557
no. of obsd refns ($I > 2\sigma(I)$)	4501	8654
No. of params	287	678
Final R, R_w ($I > 2\sigma(I)$)	0.0343, 0.1080	0.0589, 0.1344
Goodness-of-fit on F^2	0.915	1.025
$\Delta\rho_{max, min}$, eÅ^{-3}	0.192, -0.211	0.614, -0.381

	**2**	**3**·$2Me_3SiOSiMe_3$
formula	$C_{51}H_{80}N_5OP_2Sc$	$C_{77}H_{128}N_5O_2P_2ScSi_4$
fw	886.10	1375.10
color	red	orange
cryst syst.	triclinic	monoclinic
space group	$P\bar{1}$	$P2_1/c$
a, Å	9.077(1)	15.501(1)
b, Å	13.920(1)	21.182(1)
c, Å	21.718(1)	26.015(1)
α, deg	83.163(1)	90
β, deg	89.581(1)	98.828(2)
γ, deg	74.536(1)	90
V, Å^3	2625.2(1)	8440.5(4)
Z	2	4
D_{calcd}, (g/cm^3)	1.121	1.082
F(000)	960	2992
T(K)	170	170
θ range(deg)	2.887 to 54.985	3.268 to 54.928
no. of refns collected	34209	63982
no. of unique refns	9964	15956
no. of obsd refns ($I > 2\sigma(I)$)	6459	9796
No. of params	558	855
Final R, R_w ($I > 2\sigma(I)$)	0.0788, 0.2014	0.0789, 0.2009
Goodness-of-fit on F^2	1.023	1.028
$\Delta\rho_{max, min}$, eÅ^{-3}	0.639, -0.646	0.708, -0.624

	0.5 **4**	**5**
formula	$C_{32.50}H_{43.50}N_3PSc_{0.50}$	$C_{57}H_{83}N_4P_2Sc$
fw	529.65	931.17
color	blue	yellow
cryst syst.	monoclinic	Triclinic
space group	*C*2/*c*	$P\bar{1}$
a, Å	45.967(1)	12.885(1)
b, Å	13.441(1)	12.983(1)
c, Å	22.905(1)	20.810(1)
α, deg	90	78.623(4)
β, deg	117.427(1)	79.549(5)
γ, deg	90	86.876(4)
V, $Å^3$	12561.1(5)	3355.3(4)
Z	16	2
D_{calcd}, (g/cm^3)	1.120	0.920
F(000)	4560	1008
T(K)	170	170
θ range(deg)	3.012 to 54.974	3.035 to 55.459
no. of refns collected	88360	34110
no. of unique refns	11922	12751
no. of obsd refns ($I > 2\sigma(I)$)	8396	8325
No. of params	685	595
Final R, R_w ($I > 2\sigma(I)$)	0.0591, 0.1145	0.1059, 0.3150*
Goodness-of-fit on F^2	1.017	1.090
$\Delta\rho_{max, min}$, $eÅ^{-3}$	0.320, -0.505	0.675, -0.999

* We had the difficulty in growing single crystals of **5** in high quality, the structural data of **5** presented is the best one we had.

	**6**	**7**
formula	$C_{63}H_{85}N_4P_2Sc$	$C_{64}H_{87}N_4P_2Sc$
fw	1005.24	1019.27
color	orange	orange
cryst syst.	orthorhombic	triclinic
space group	*Cmca*	$P\bar{1}$
a, Å	17.560(1)	13.374(1)
b, Å	19.084(1)	13.423(1)
c, Å	40.689(1)	19.781(1)
α, deg	90	88.642(1)
β, deg	90	72.375(1)
γ, deg	90	61.270(1)
V, Å^3	13635.2(5)	2936.0(1)
Z	8	2
D_{calcd}, (g/cm^3)	0.979	1.153
F(000)	4336	1100
T(K)	170	170
θ range(deg)	3.122 to 55.009	3.403 to 54.895
no. of refns collected	73276	31517
no. of unique refns	6717	11092
no. of obsd refns ($I > 2\sigma(I)$)	4774	9294
No. of params	343	659
Final R, R_w (I > 2σ(I))	0.0649, 0.1712	0.0453, 0.1107
Goodness-of-fit on F^2	1.036	1.024
$\Delta\rho_{max, min}$, eÅ^{-3}	1.577, -0.469	0.430, -0.524

	**8**	**9**
formula	$C_{55}H_{78}N_5P_2Sc$	$C_{56}H_{80}N_5P_2Sc$
fw	916.12	930.15
color	yellow	yellow
cryst syst.	monoclinic	orthorhombic
space group	$P2_1/n$	*Pbca*
a, Å	21.463(1)	23.372(1)
b, Å	11.283(1)	16.724(1)
c, Å	23.269(1)	27.947(1)
α, deg	90	90
β, deg	106.909(1)	90
γ, deg	90	90
V, $Å^3$	5391.3(2)	10923.7(3)
Z	4	8
D_{calcd}, (g/cm^3)	1.129	1.131
F(000)	1976	4016
T(K)	170	170
θ range(deg)	2.893 to 54.964	3.144 to 54.967
no. of refns collected	58266	86992
no. of unique refns	10241	10383
no. of obsd refns ($I > 2\sigma(I)$)	6911	7405
No. of params	608	594
Final R, R_w ($I > 2\sigma(I)$)	0.0562, 0.1090	0.0479, 0.1026
Goodness-of-fit on F^2	1.028	1.026
$\Delta\rho_{max, min}$, e$Å^{-3}$	0.354, -0.338	0.384, -0.324

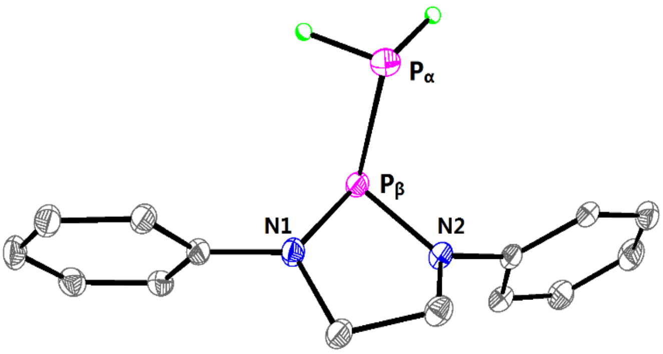

Supplementary Figure 1. Molecular structure of $H_2PP\{N(DIPP)CH_2CH_2N(DIPP)\}$ with ellipsoids at 30% probability level. DIPP isopropyl groups and hydrogen atoms (except the hydrogen atoms on P_α) were omitted for clarity. Selected bond distances [Å] and angles [°]: P_α–P_β 2.277(1), P_β–N1 1.697(2), P_β–N2 1.684(2); P_α–P_β–N1 98.1(1), P_α–P_β–N2 102.8(1), N1–P_β–N2 91.0(1).

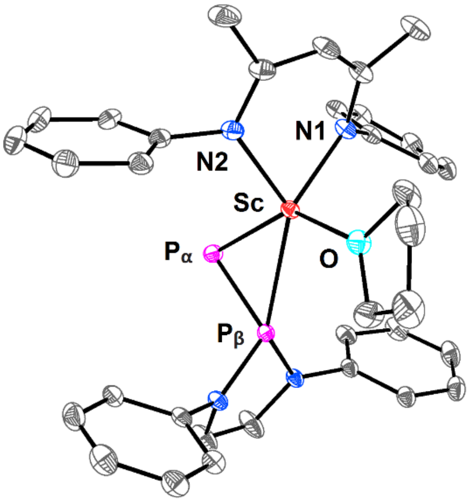

Supplementary Figure 2. Molecular structure of **1** with ellipsoids at 30% probability level. DIPP isopropyl groups and hydrogen atoms were omitted for clarity. Selected bond distances [Å] and angles [°]: Sc–N1 2.180(2), Sc–N2 2.196(2), Sc–O 2.197(2), Sc–P_α 2.448(1), Sc–P_β 2.718(1), P_α–P_β 2.105(1); Sc–P_α–P_β 72.9(1), Sc–P_β–P_α 59.4(1), P_α–Sc–P_β 47.7(1).

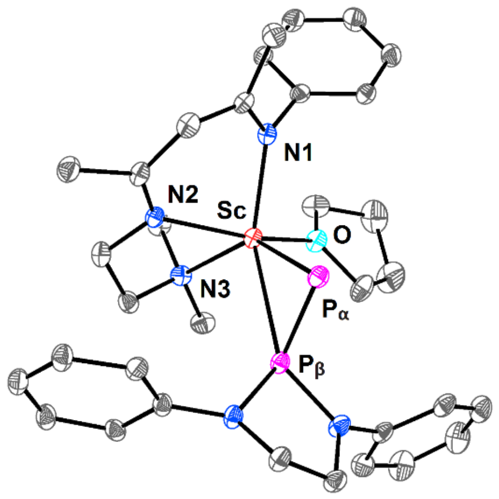

Supplementary Figure 3. Molecular structure of **2** with ellipsoids at 30% probability level. DIPP isopropyl groups and hydrogen atoms were omitted for clarity. Selected bond distances [Å] and angles [°]: Sc−N1 2.194(3), Sc−N2 2.183(3), Sc−N3 2.393(3), Sc−O 2.275(2), Sc−P_α 2.484(1), Sc−P_β 2.814(1), P_α−P_β 2.095(1); Sc−P_α−P_β 75.3(1), Sc−P_β−P_α 58.6(1), P_α−Sc−P_β 46.1(1).

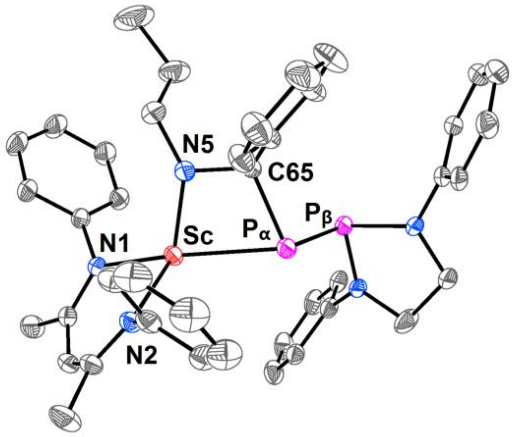

Supplementary Figure 4. Molecular structure of **3** with ellipsoids at 30% probability level. DIPP isopropyl groups and hydrogen atoms were omitted for clarity. Selected bond distances [Å] and angles [°]: Sc−N1 2.132(3), Sc−N2 2.185(3), Sc−N5 1.933(3), Sc−P_α 2.547(1), N5−C56 1.479(5), C56−P_α 1.949(4), P_α−P_β 2.211(1); Sc−P_α−P_β 121.3(1), N5−Sc−P_α 76.7(1), N5−C56−P_α 110.1(2), Sc−P_α−C56 71.3(1), Sc−N5−C56 101.9(2).

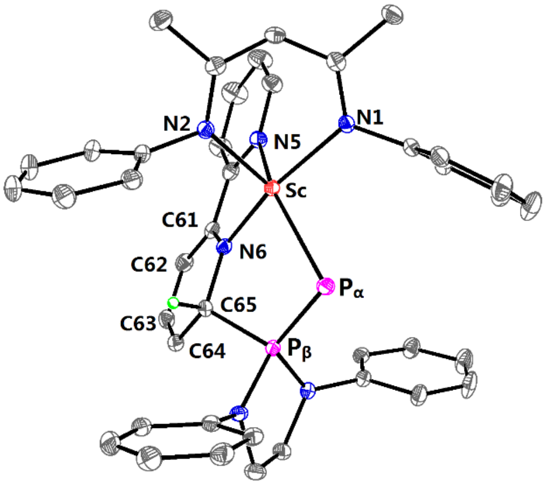

Supplementary Figure 5. Molecular structure of **4** with ellipsoids at 30% probability level. DIPP isopropyl groups and hydrogen atoms (except the hydrogen atom on C65) were omitted for clarity. DIPP isopropyl groups and hydrogen atoms (except the hydrogen atom on C65) were omitted for clarity. Selected bond distances [Å] and angles [°]: Sc−N1 2.195(2), Sc−N2 2.167(2), Sc−N5 2.299(2), Sc−N6 2.124(2), Sc−$P_α$ 2.557(1), $P_α$−$P_β$ 2.027(1), $P_β$−C65 1.886(3), N6−C61 1.370(3), N6−C65 1.487(3), C61−C62 1.365(4), C62−C63 1.428(4), C63−C64 1.336(4), C64−C65 1.502(4); Sc−$P_α$−$P_β$ 96.7(1), $P_α$−$P_β$−C65 110.9(1), $P_α$−Sc−N6 88.4(1), Sc−N6−C65 119.1(2), $P_β$−C65−N6 108.5(2).

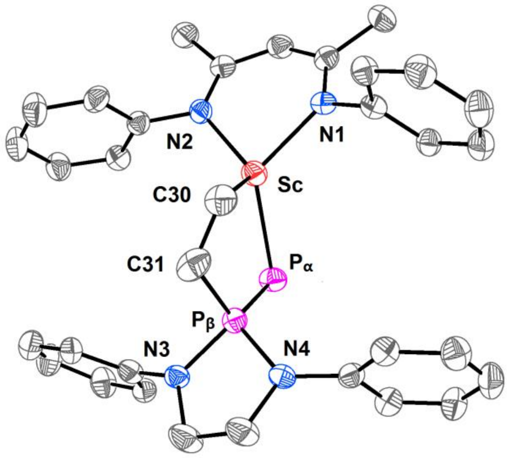

Supplementary Figure 6. Molecular structure of **5** with ellipsoids at 30% probability level. DIPP isopropyl groups and hydrogen atoms were omitted for clarity. Selected bond distances [Å] and angles [°]: Sc−N1 2.120(4), Sc−N2 2.143(4), Sc−$P_α$ 2.535(2), Sc−C30 2.126(5), $P_α$−$P_β$ 2.021(1), C30−C31 1.424(8), C31−$P_β$ 1.840(5), $P_β$−N3 1.721(4), $P_α$−N4 1.729(4), Sc−$P_α$−$P_β$ 89.7(1), $P_α$−$P_β$−C31 115.3(2), $P_β$−C31−C30 117.1(4), Sc−C30−C31 110.4(3), $P_α$−Sc−C30 96.9(2).

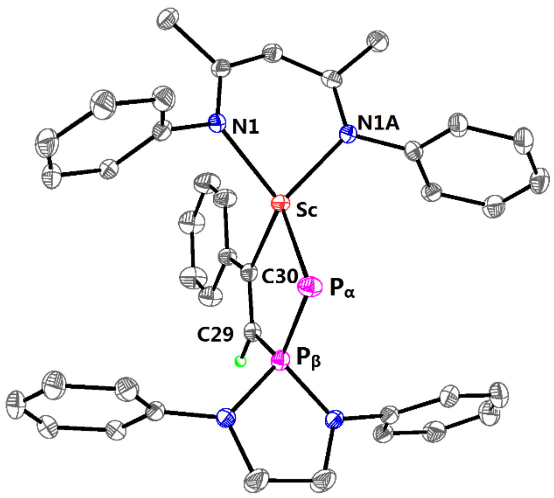

Supplementary Figure 7. Molecular structure of **6** with ellipsoids at 30% probability level. DIPP isopropyl groups and hydrogen atoms (except the hydrogen atom on C29) were omitted for clarity. Selected bond distances [Å] and angles [°]: Sc−N1 2.158(2), Sc−P_α 2.505(1), Sc−C30 2.253(4), P_α−P_β 2.097(2), C29−C30 1.342(5), P_β−C29 1.797(4); Sc−P_α−P_β 91.1(1), P_α−P_β−C29 115.7(1), P_β−C29−C30 123.6(3), Sc−C30−C29 113.9(3), P_α−Sc−C30 95.8(1).

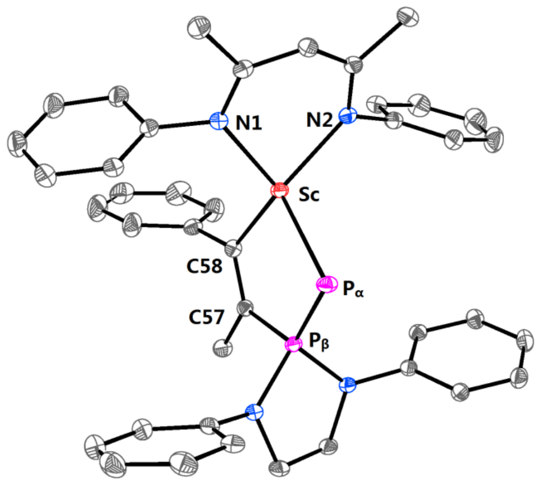

Supplementary Figure 8. Molecular structure of **7** with ellipsoids at 30% probability level. DIPP isopropyl groups and hydrogen atoms were omitted for clarity. Selected bond distances [Å] and angles [°]: Sc−N1 2.136(2), Sc−N2 2.189(2), Sc−P_α 2.508(1), Sc−C58 2.234(2), P_α−P_β 2.050(1), C57−C58 1.350(3), P_β−C57 1.844(2); Sc−P_α−P_β 92.4(1), P_α−P_β−C57 115.9(1), P_β−C57−C58 120.2(1), Sc−C58−C57 116.5(1), P_α−Sc−C58 94.4(1).

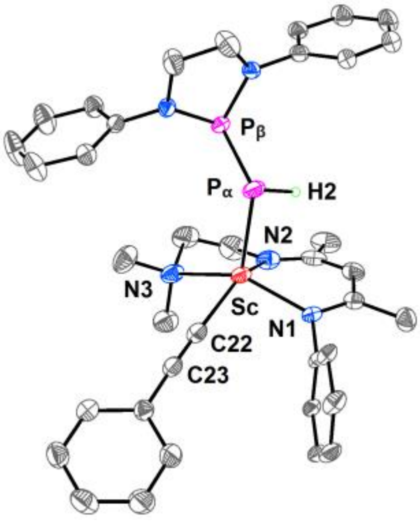

Supplementary Figure 9. Molecular structure of **8** with ellipsoids at 40% probability level. DIPP isopropyl groups and hydrogen atoms (except the hydrogen atom on P_α) were omitted for clarity. Selected bond distances [Å] and angles [°]: Sc−N1 2.146(2), Sc−N2 2.183(3), Sc−N3 2.313(2), Sc−P_α 2.618(1), Sc−C22 2.215(3), P_α−P_β 2.229(1), C22–C23 1.208(4); Sc–P_α−P_β 111.6(1), Sc–C22–C23 178.5(3), P_α–Sc–C22 110.2(1).

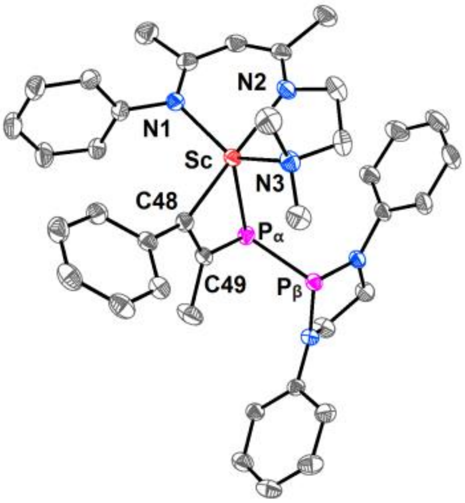

Supplementary Figure 10. Molecular structure of **9** with ellipsoids at 40% probability level. DIPP isopropyl groups and hydrogen atoms were omitted for clarity. Selected bond distances [Å] and angles [°]: Sc−N1 2.198(2), Sc−N2 2.204(2), Sc−N3 2.331(2), Sc–P_α 2.544(1), Sc−C48 2.175(2), P_α−P_β 2.222(1), P_α–C49 1.912(2), C48–C49 1.354(3); Sc−P_α−P_β 110.8(1), Sc−C48–C49 90.9(2), Sc–P_α–C49 69.1(1), C48–C49–P_α 124.2(2).

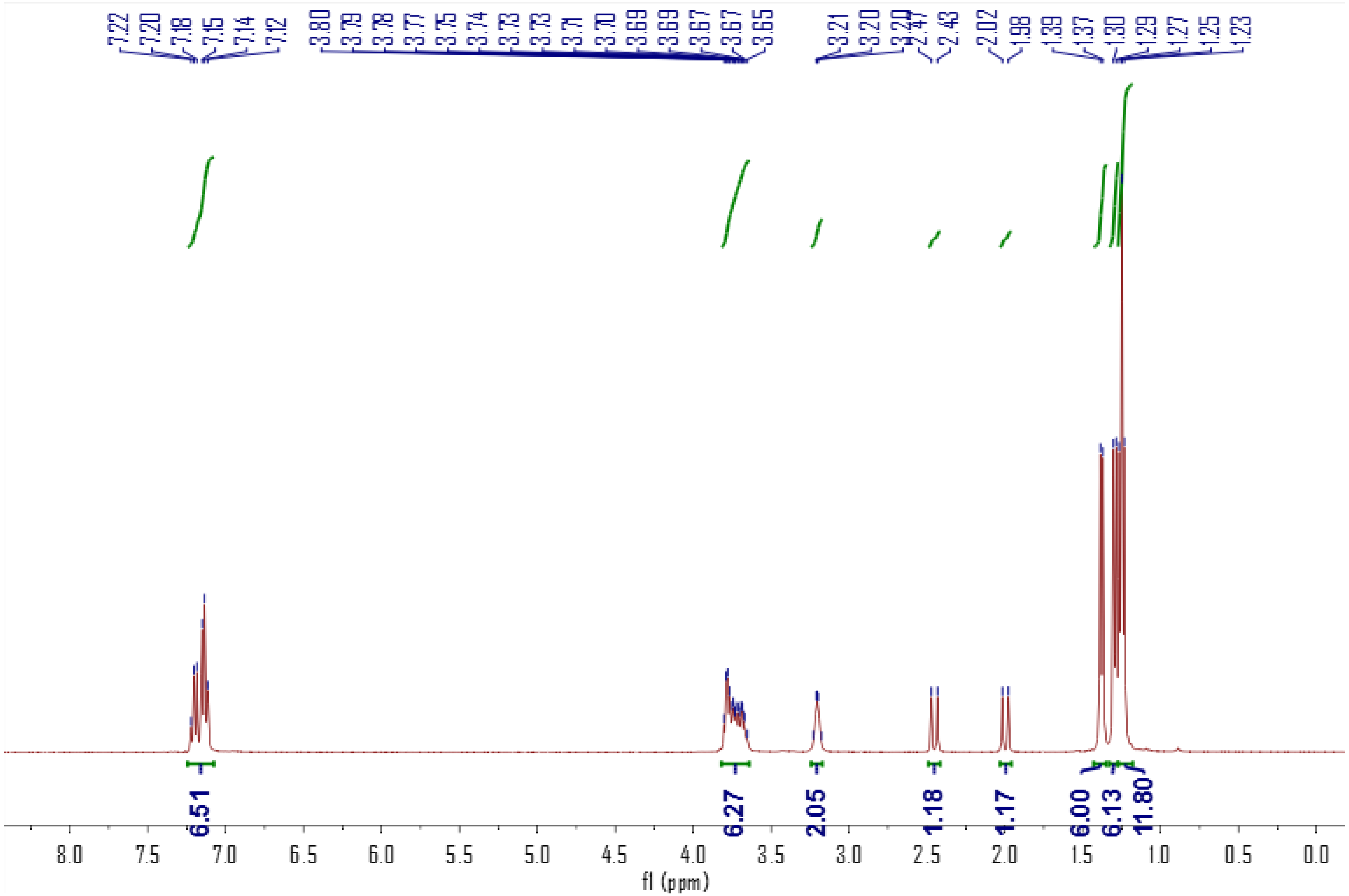

Supplementary Figure 11. ^{1}H NMR of $H_2PP\{N(DIPP)CH_2CH_2N(DIPP)\}$ (400 MHz, C_6D_6, 25 ºC).

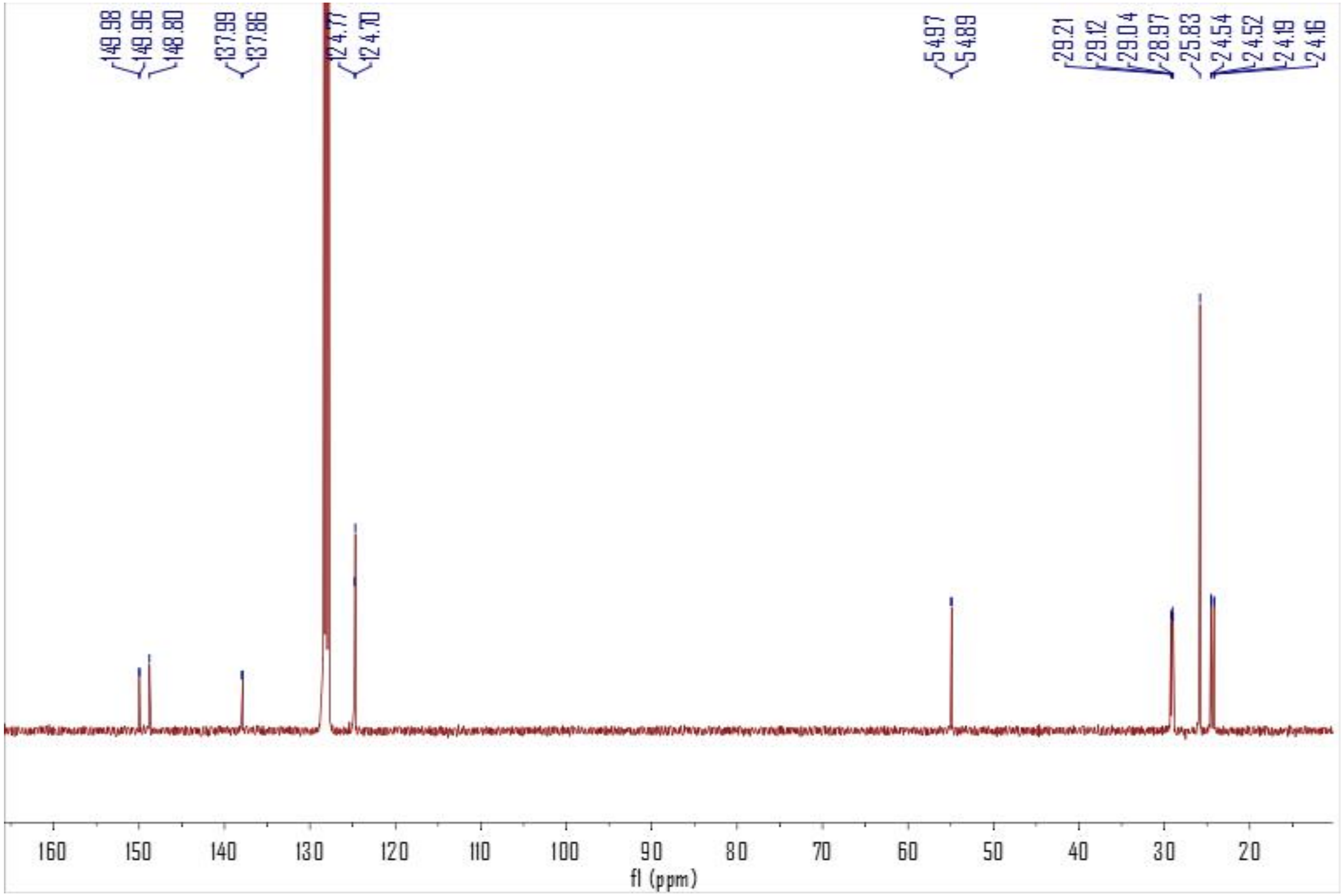

Supplementary Figure 12. $^{13}C\{^1H\}$ NMR of $H_2PP\{N(DIPP)CH_2CH_2N(DIPP)\}$ (100 MHz, C_6D_6, 25 ºC).

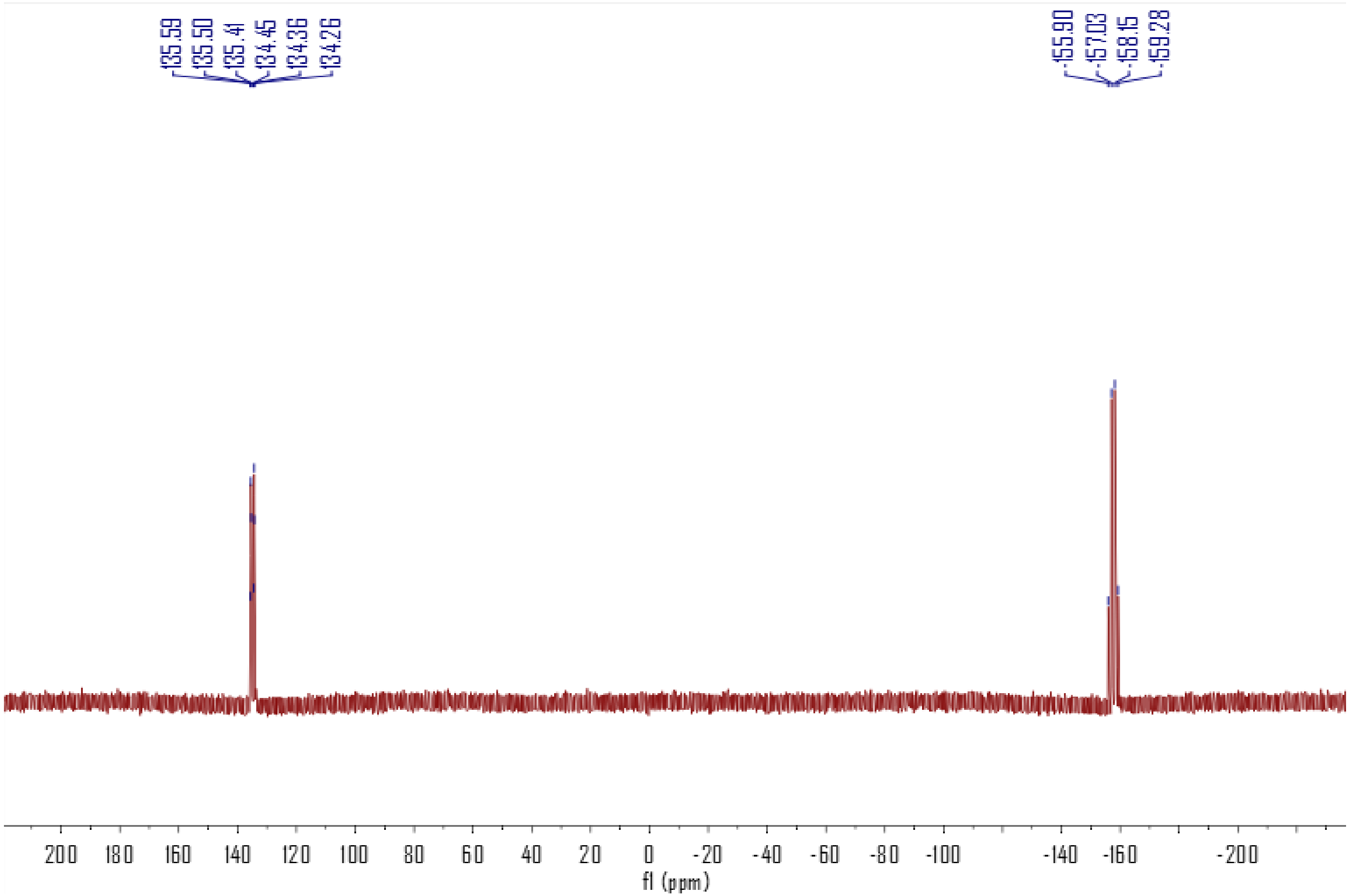

Supplementary Figure 13. ^{31}P NMR of $H_2PP\{N(DIPP)CH_2CH_2N(DIPP)\}$ (162 MHz, C_6D_6, 25 ºC).

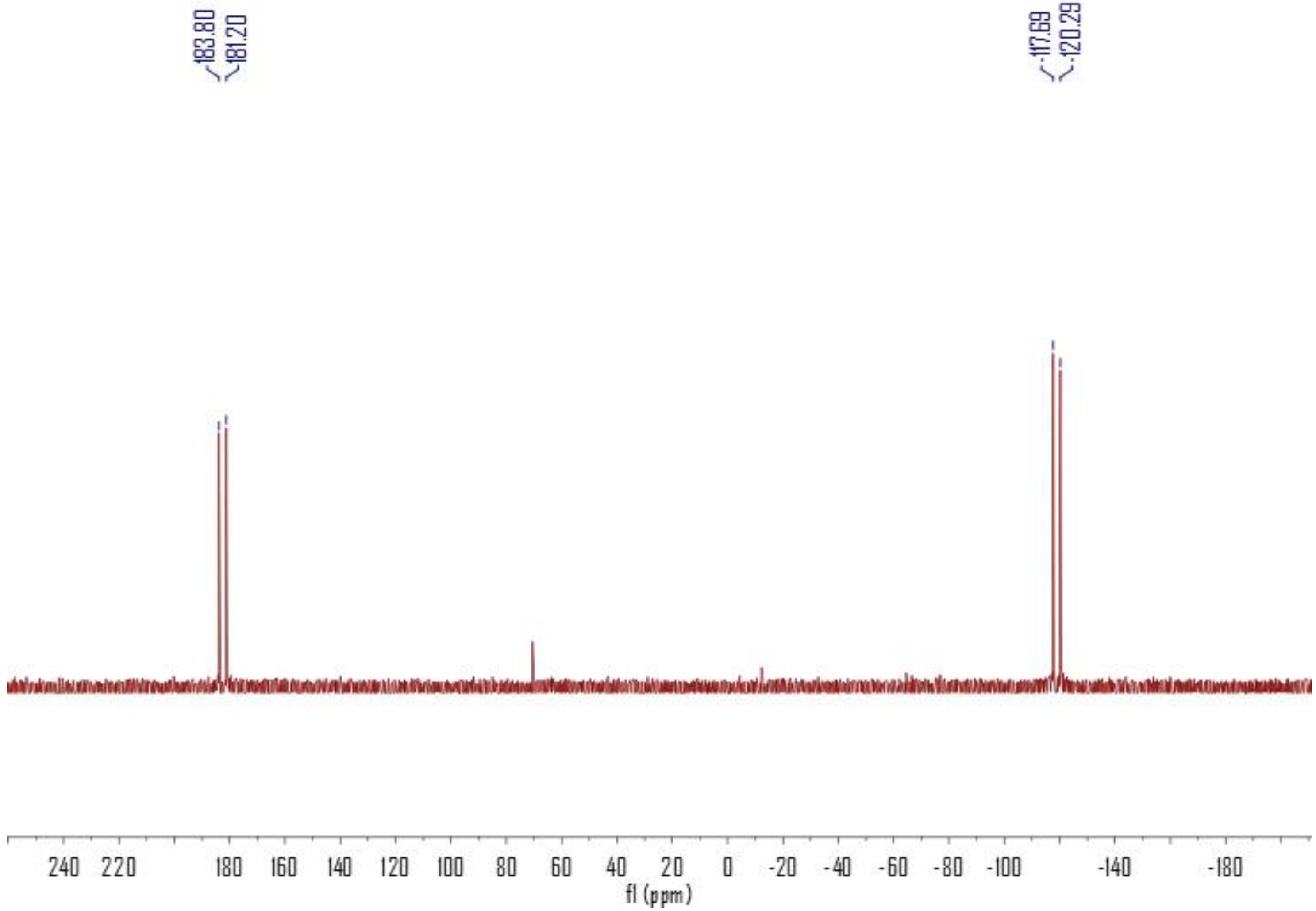

Supplementary Figure 14. $^{31}P\{^1H\}$ NMR of K[HPP{N(DIPP)CH_2CH_2N(DIPP)}] (162 MHz, THF-d_8, 25 ºC).

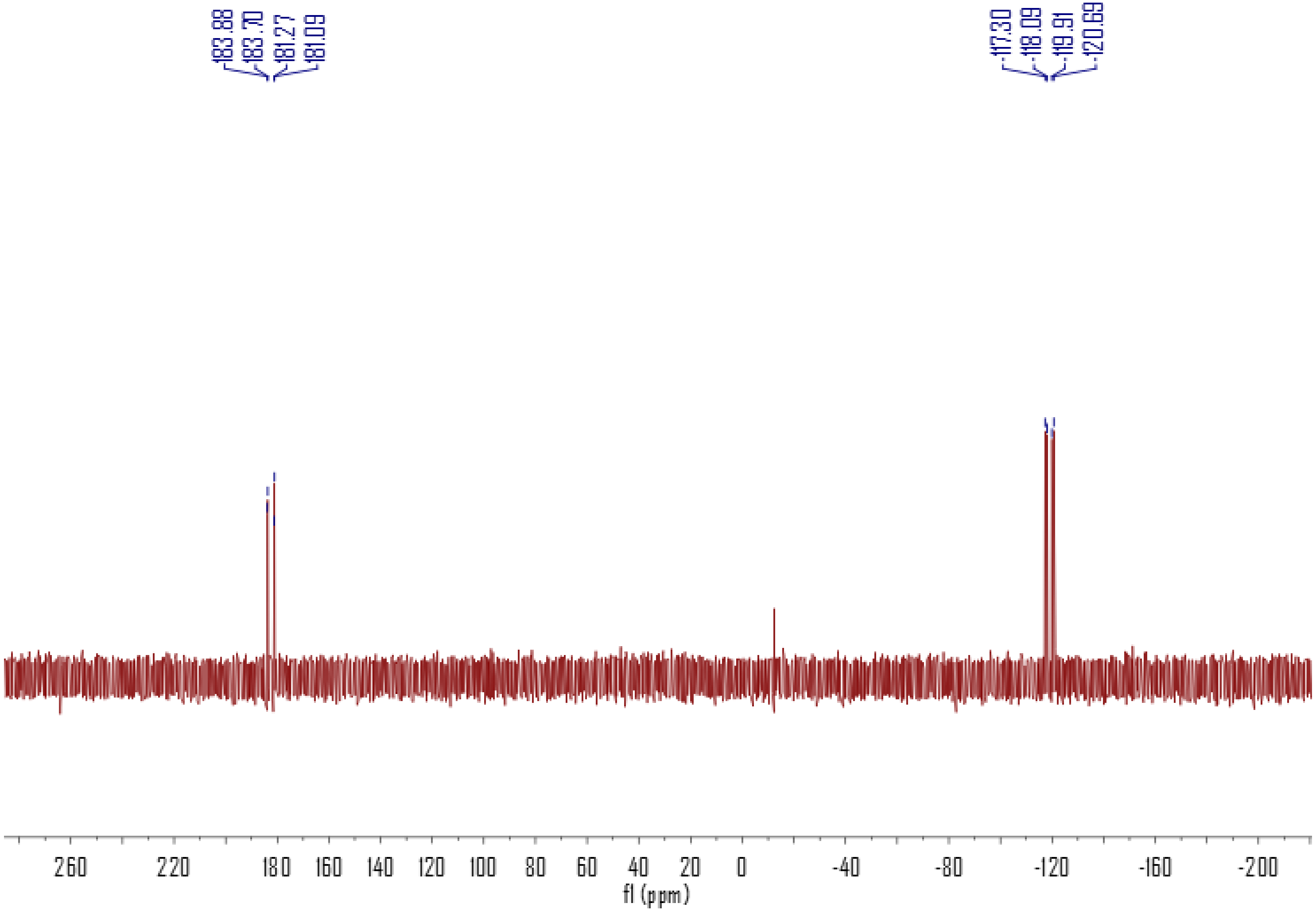

Supplementary Figure 15. ^{31}P NMR of K[HPP{N(DIPP)CH_2CH_2N(DIPP)}] (162 MHz, THF-d_8, 25 ºC).

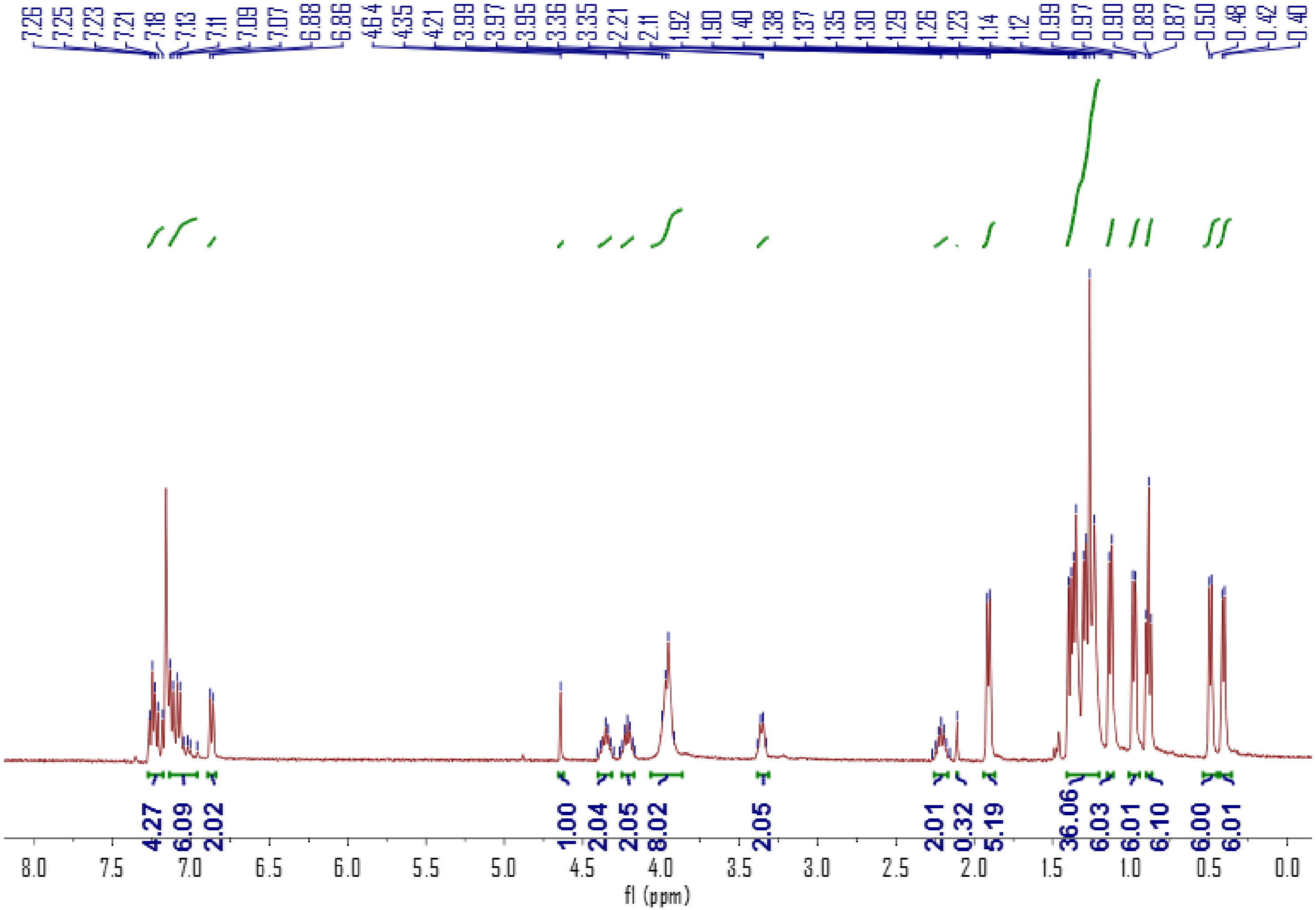

Supplementary Figure 16. ^{1}H NMR of **1**·hexane (400 MHz, C_6D_6, 25 ºC).

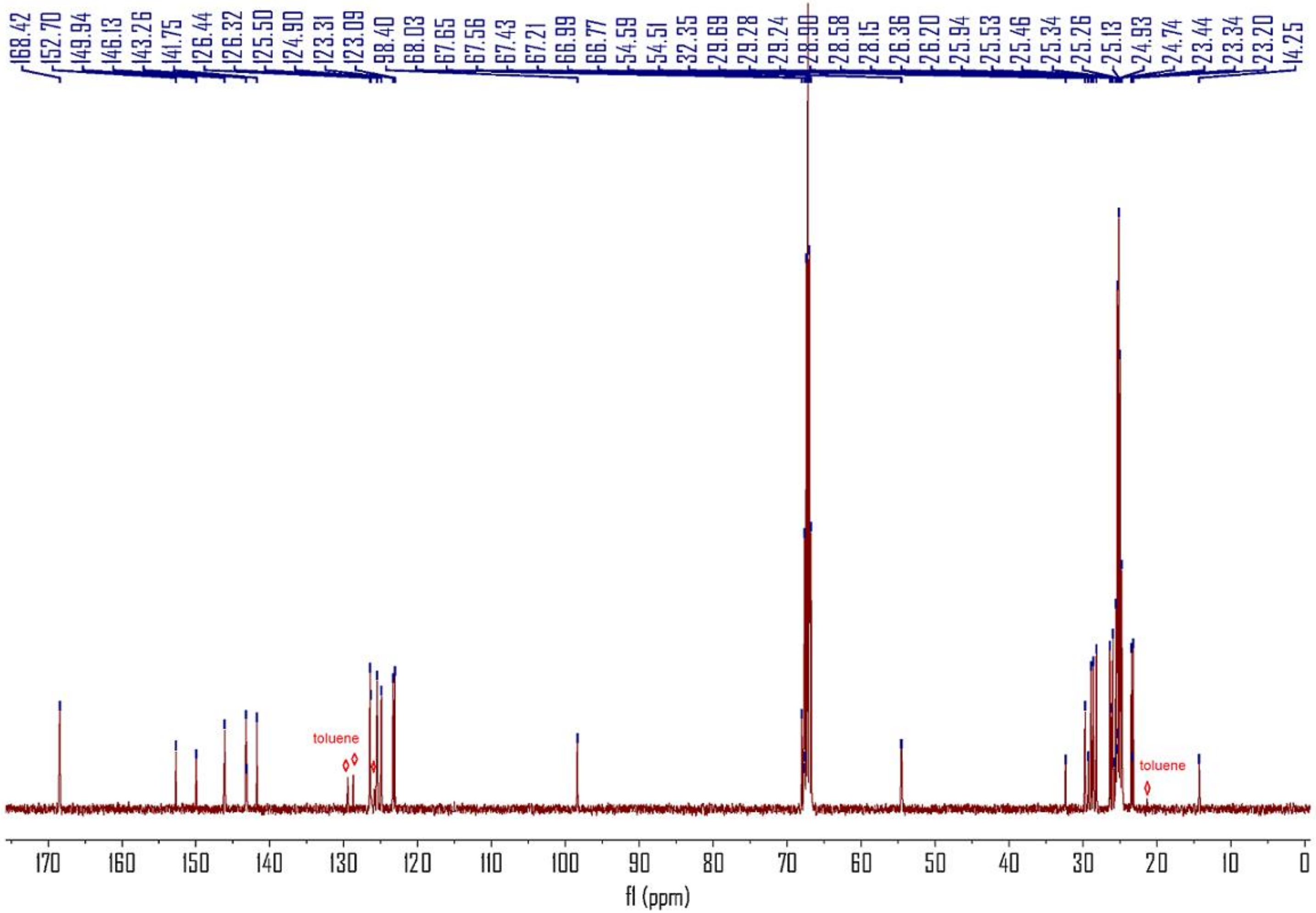

Supplementary Figure 17. $^{13}C\{^1H\}$ NMR of **1**·hexane (100 MHz, THF-d_8, 25 ºC). (There are small amount of toluene residue)

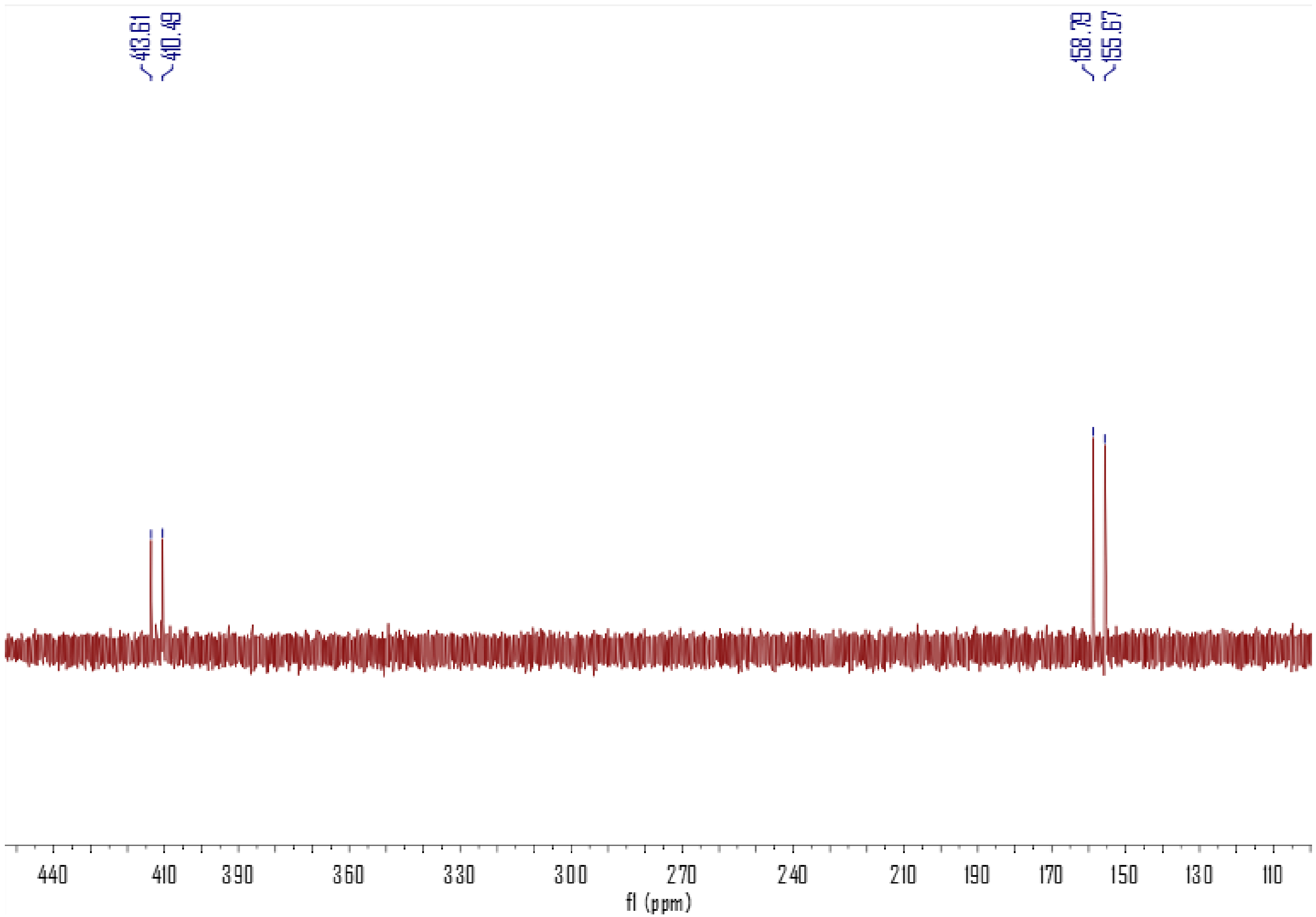

Supplementary Figure 18. $^{31}P\{^1H\}$ NMR of **1**·hexane (162 MHz, C_6D_6, 25 ºC).

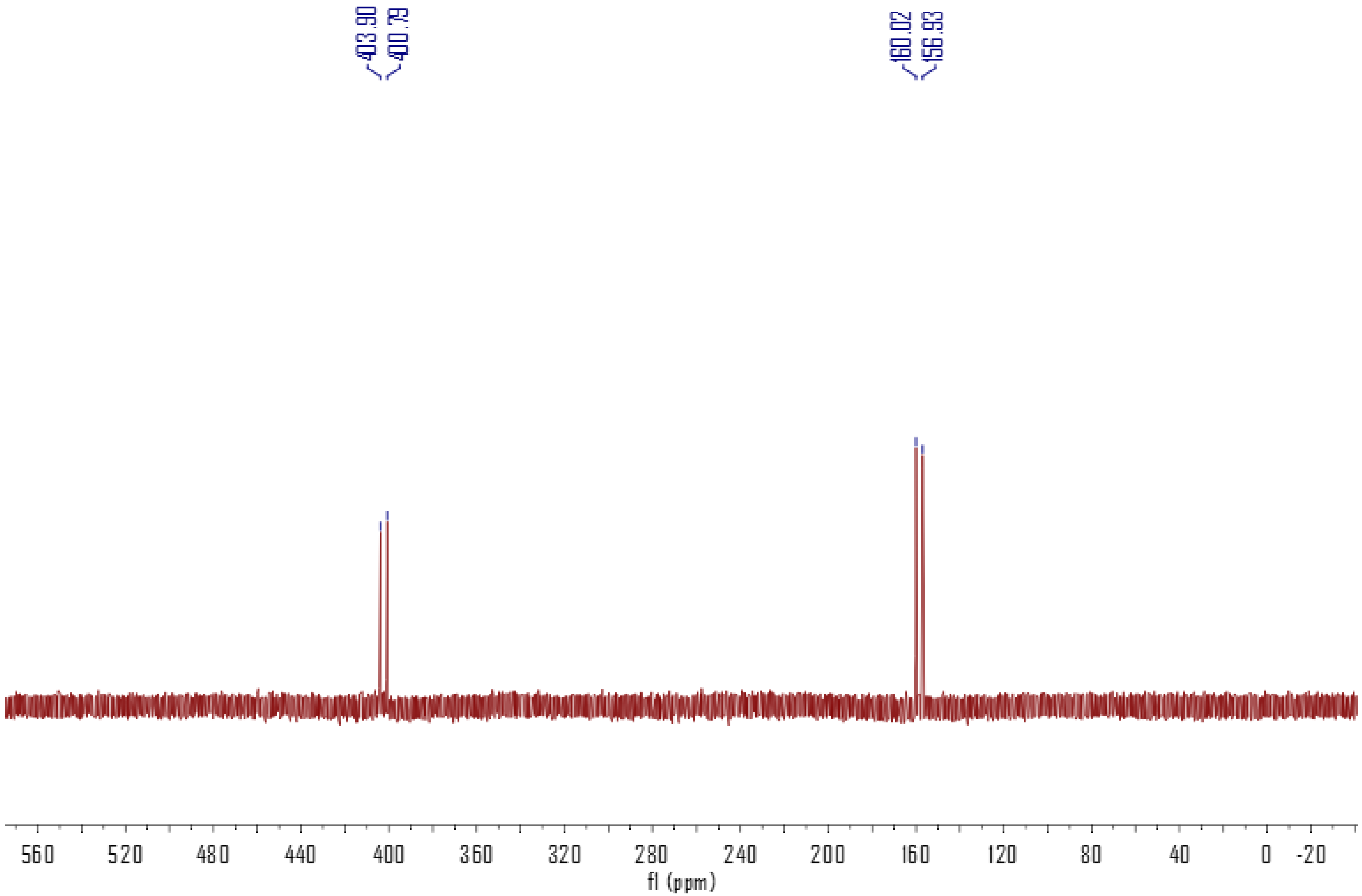

Supplementary Figure 19. $^{31}P\{^{1}H\}$ NMR of **1**·hexane (100 MHz, THF-d_8, 25 °C).

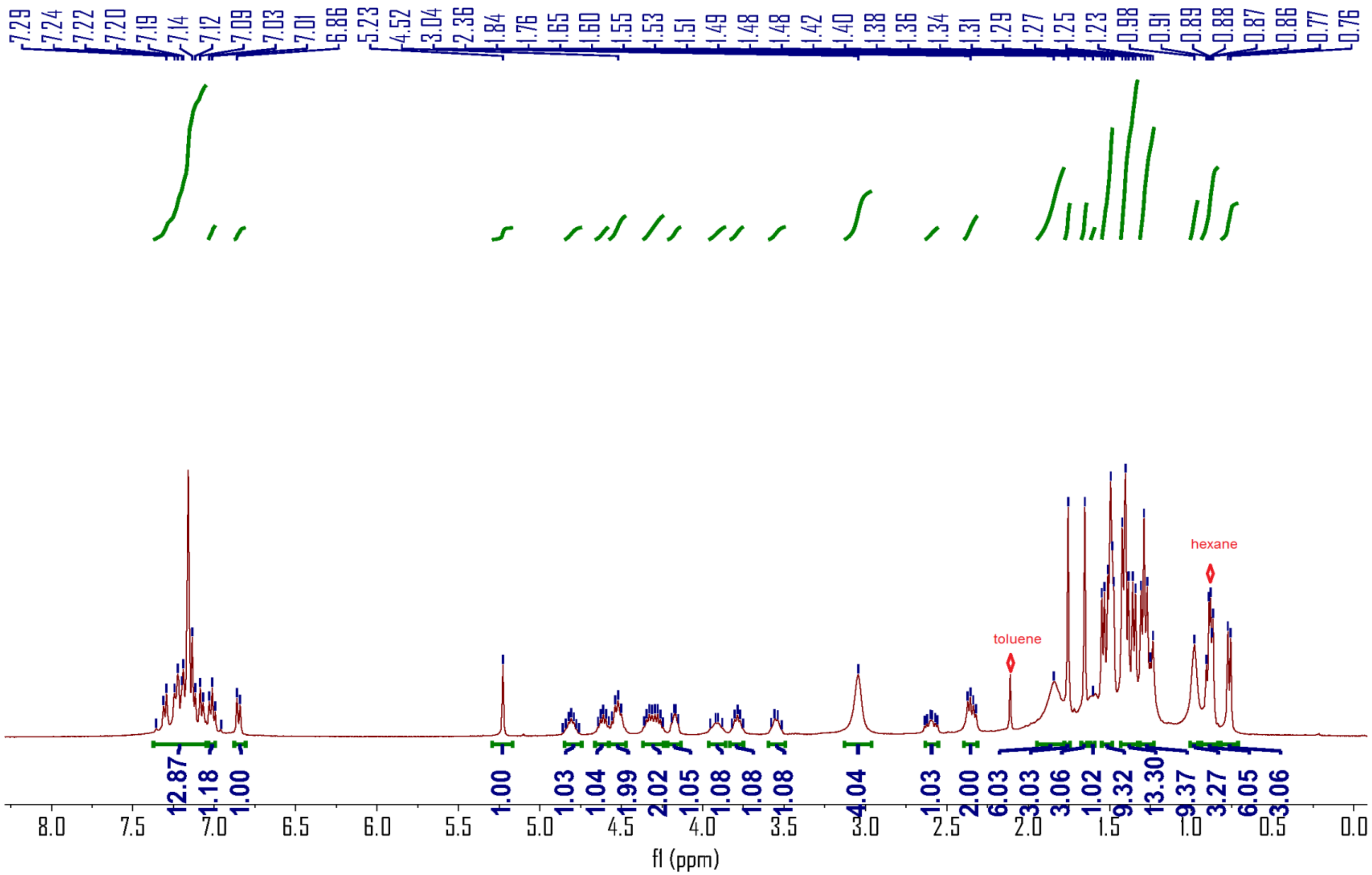

Supplementary Figure 20. ^{1}H NMR of **2** (400 MHz, C_6D_6, 25 °C). (There are small amount of toluene and hexane residue)

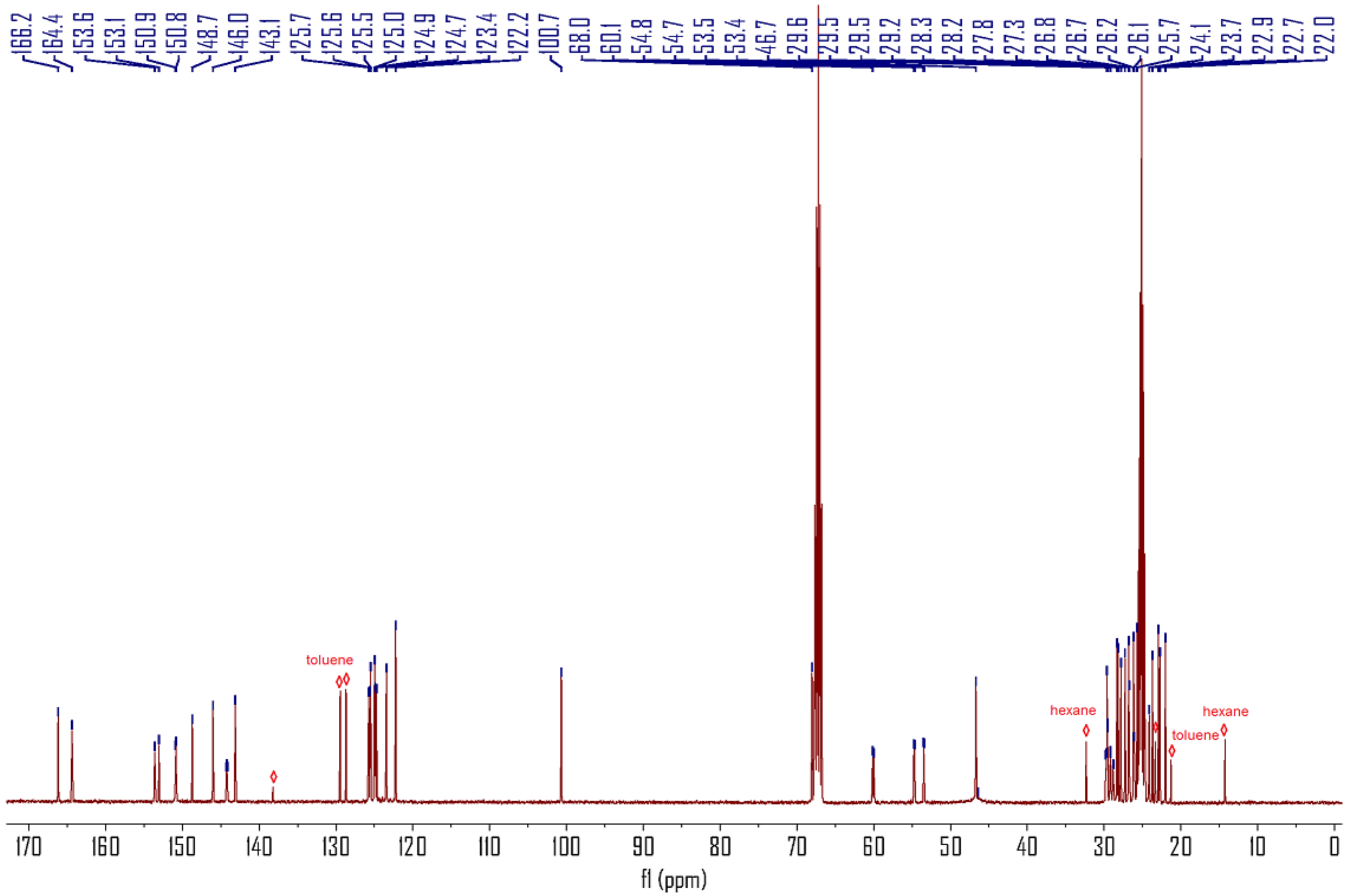

Supplementary Figure 21. $^{13}C\{^{1}H\}$ NMR of **2** (100 MHz, THF-d_8, 25 ºC). (There are small amount of toluene and hexane residue)

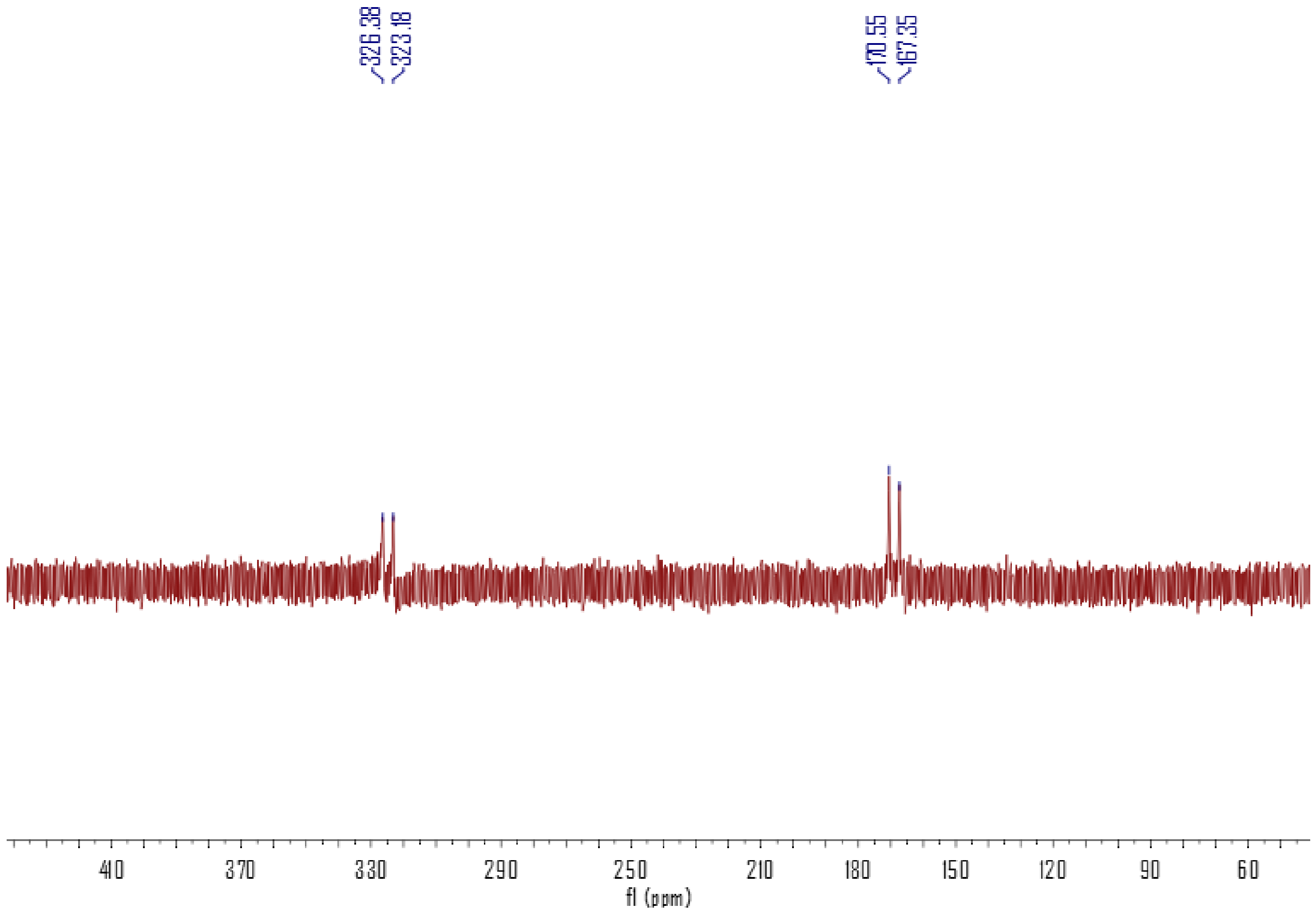

Supplementary Figure 22. $^{31}P\{^1H\}$ NMR of **2** (162 MHz, C_6D_6, 25 ºC).

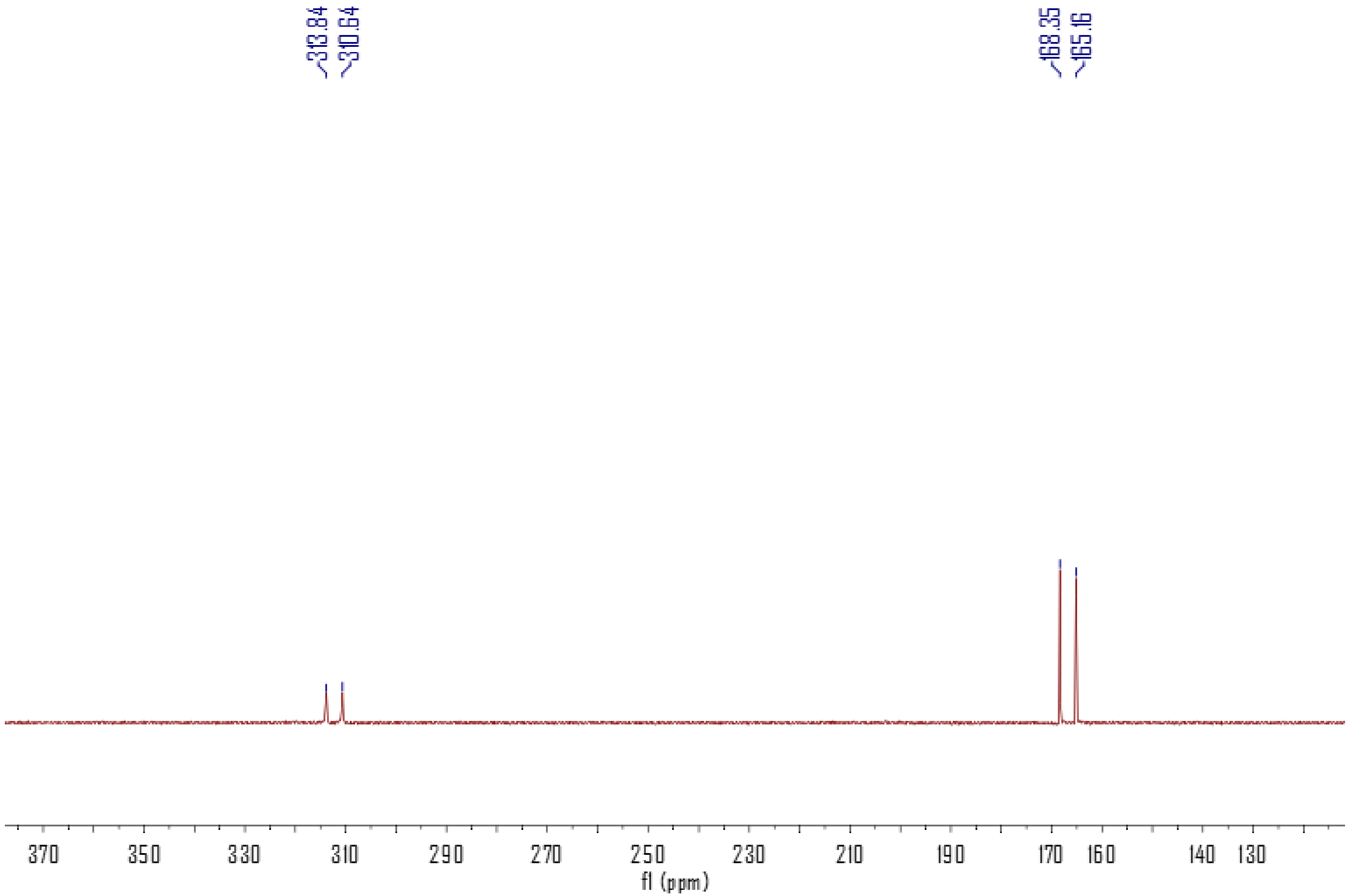

Supplementary Figure 23. $^{31}P\{^{1}H\}$ NMR of **2** (162 MHz, THF-d_8, 25 ºC).

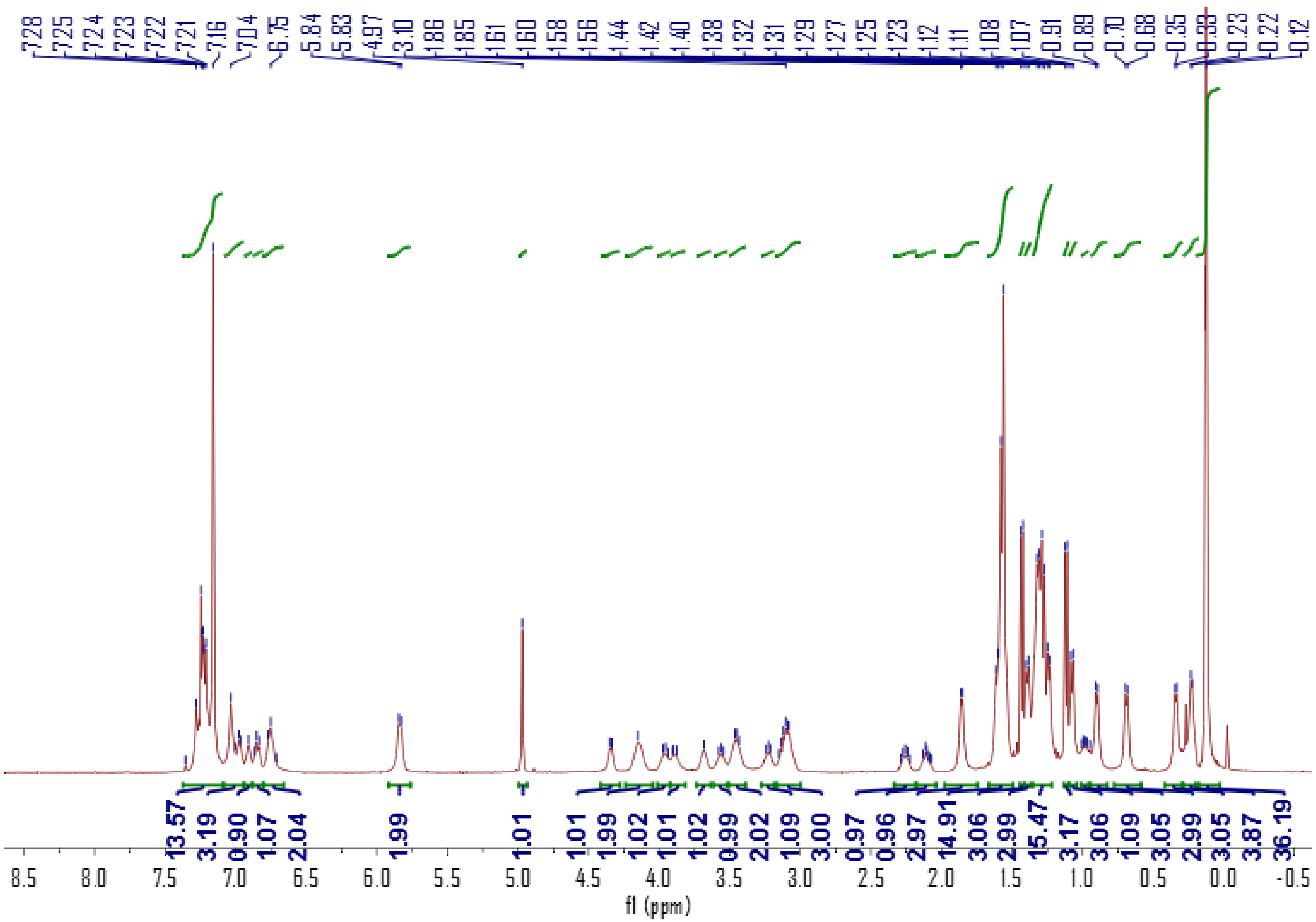

Supplementary Figure 24. ^{1}H NMR of **3**·2Me$_3$SiOSiMe$_3$ (400 MHz, C_6D_6, 25 ºC).

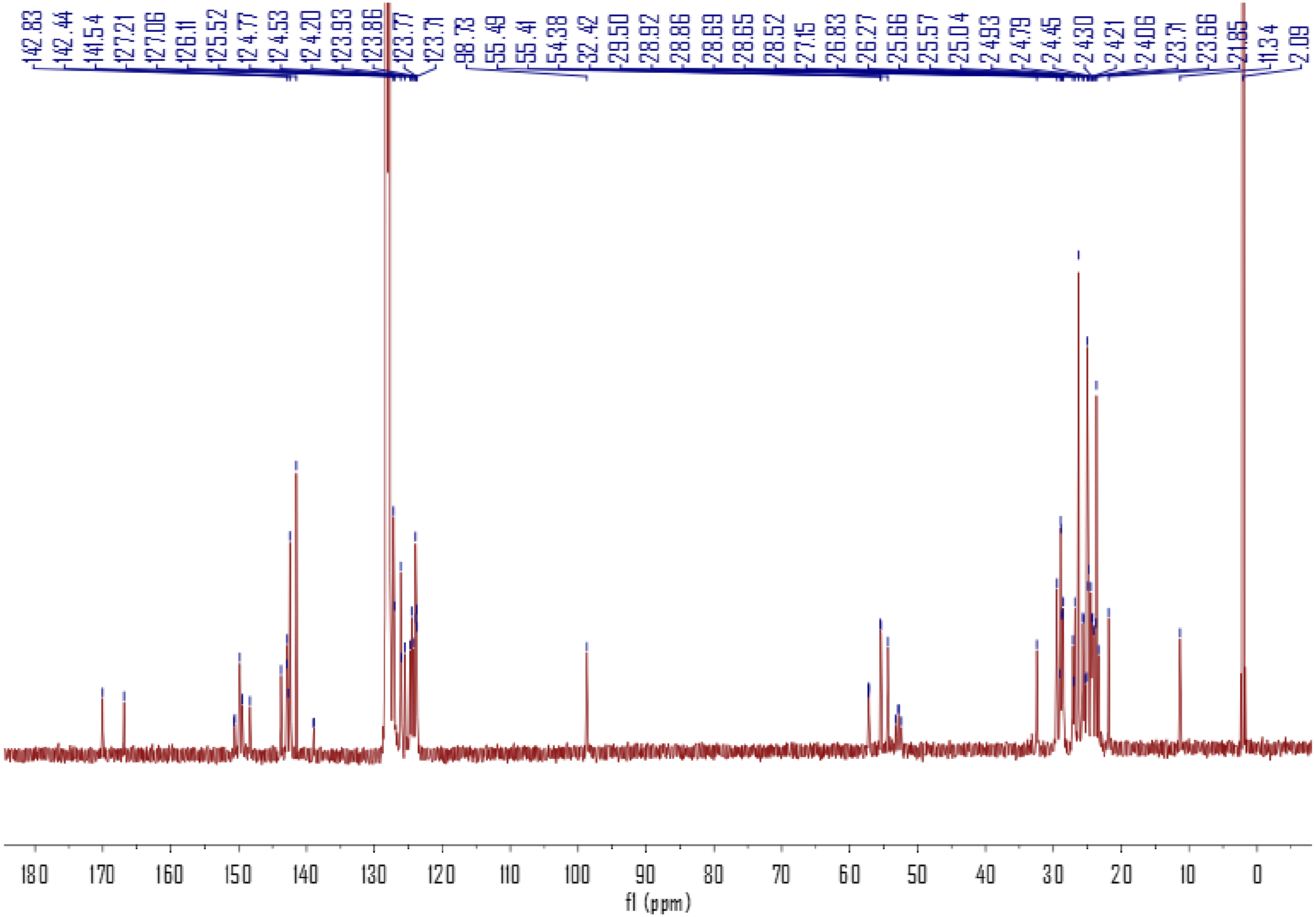

Supplementary Figure 25. $^{13}C\{^{1}H\}$ NMR of **3**·2Me$_3$SiOSiMe$_3$ (100 MHz, C_6D_6, 25 ºC).

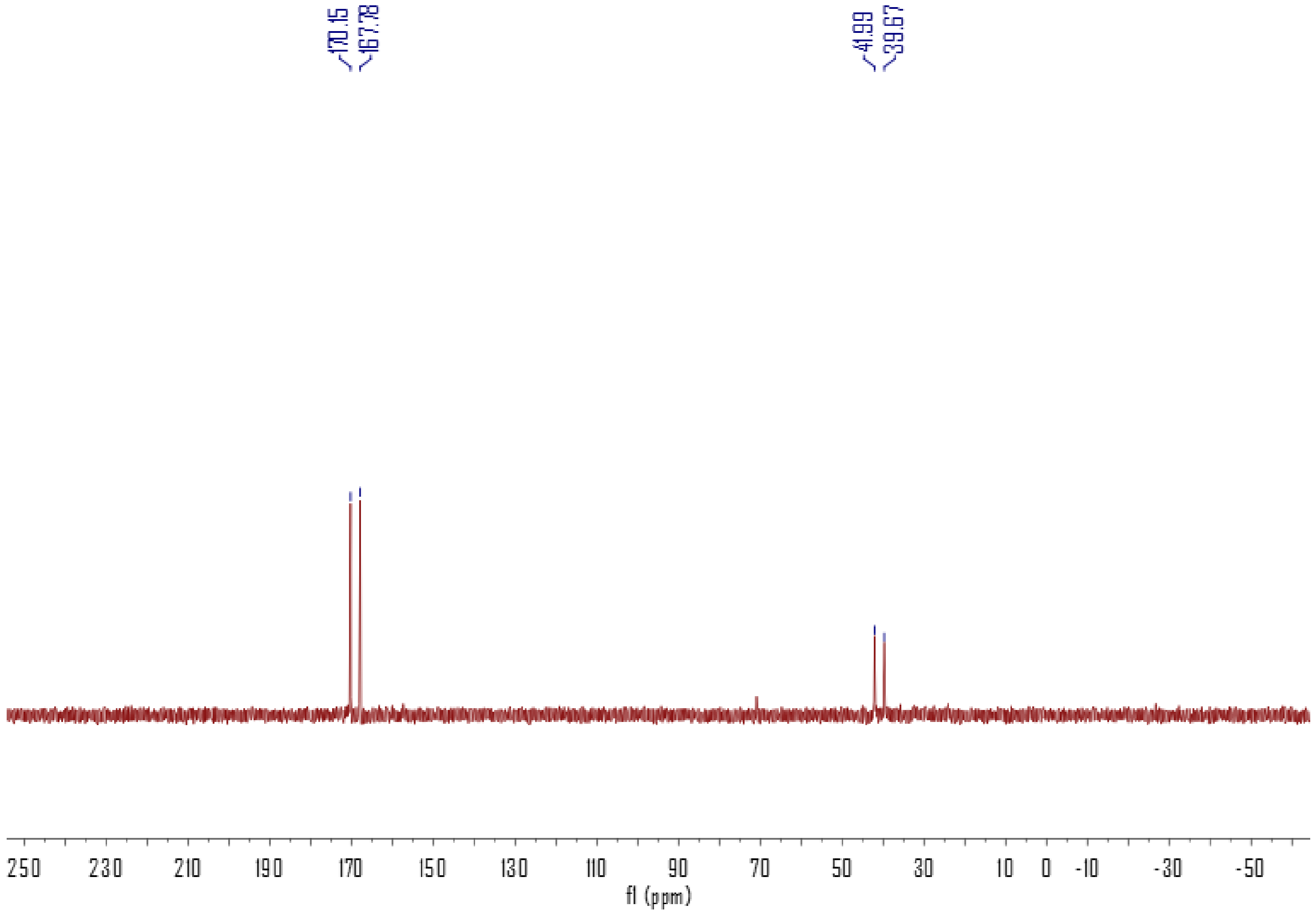

Supplementary Figure 26. $^{31}P\{^1H\}$ NMR of **3**·2Me$_3$SiOSiMe$_3$ (162 MHz, C_6D_6, 25 ºC).

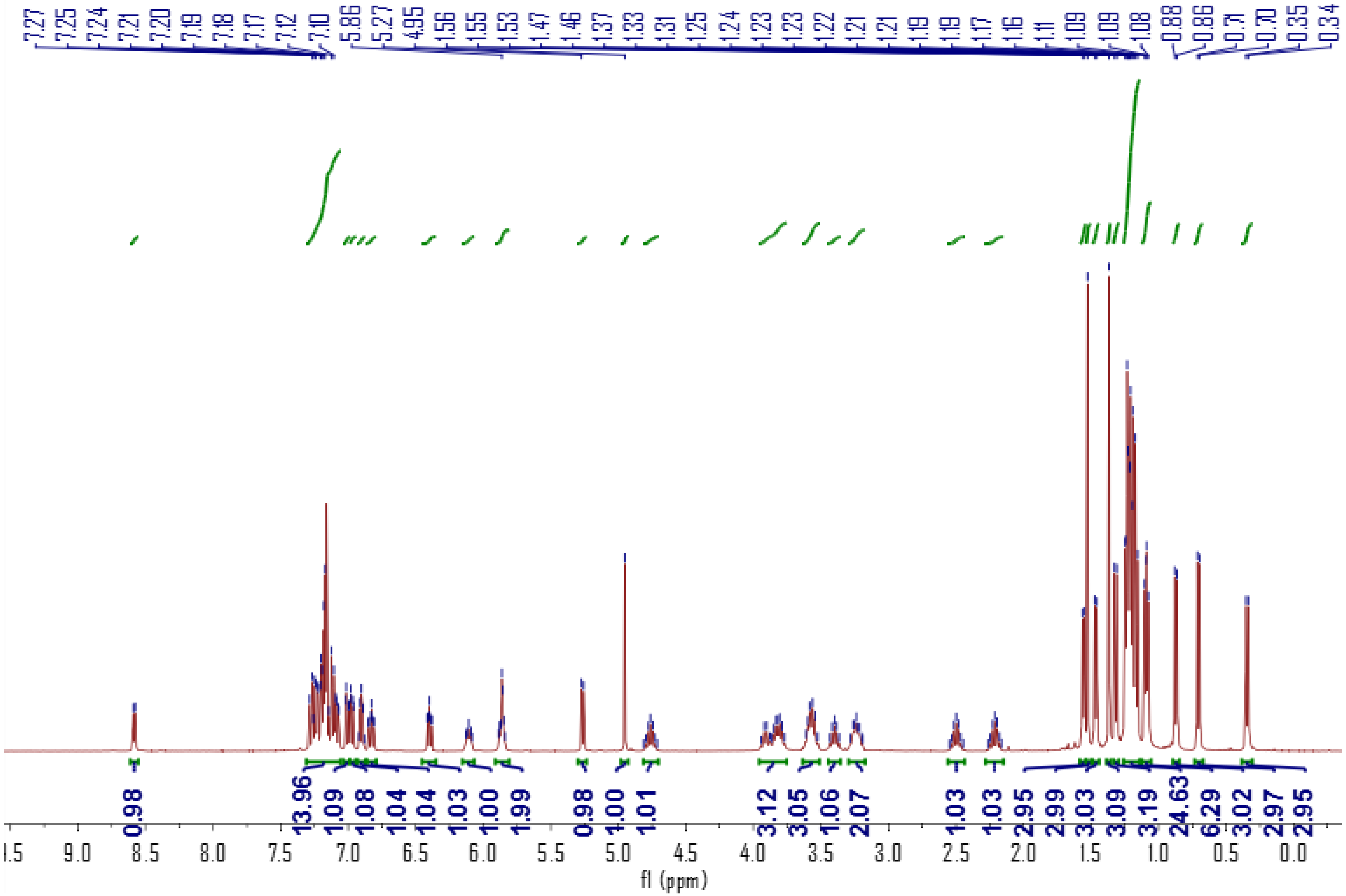

Supplementary Figure 27. ^{1}H NMR of **4** (400 MHz, C_6D_6, 25 ºC).

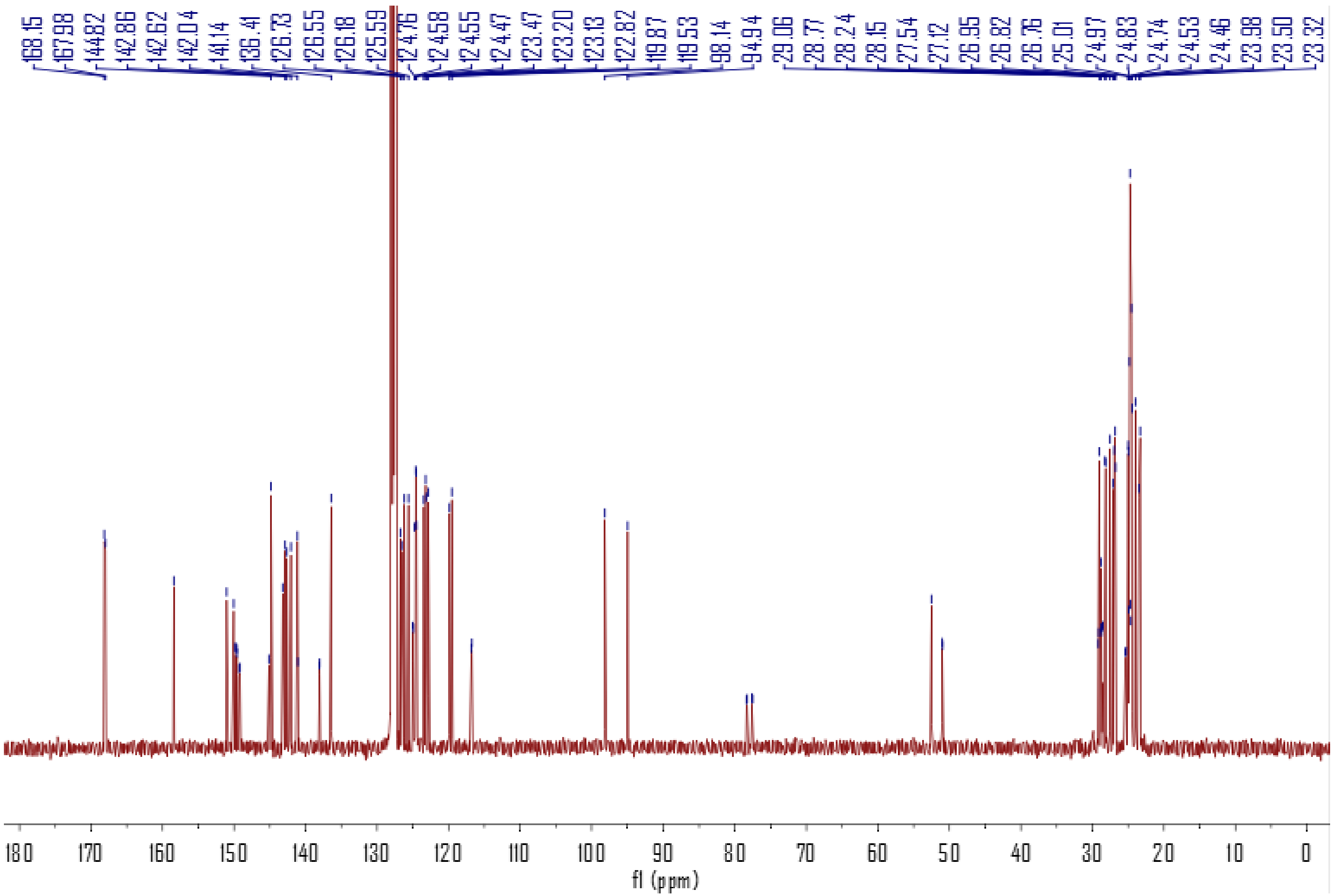

Supplementary Figure 28. $^{13}C\{^{1}H\}$ NMR of **4** (100 MHz, C_6D_6, 25 ºC).

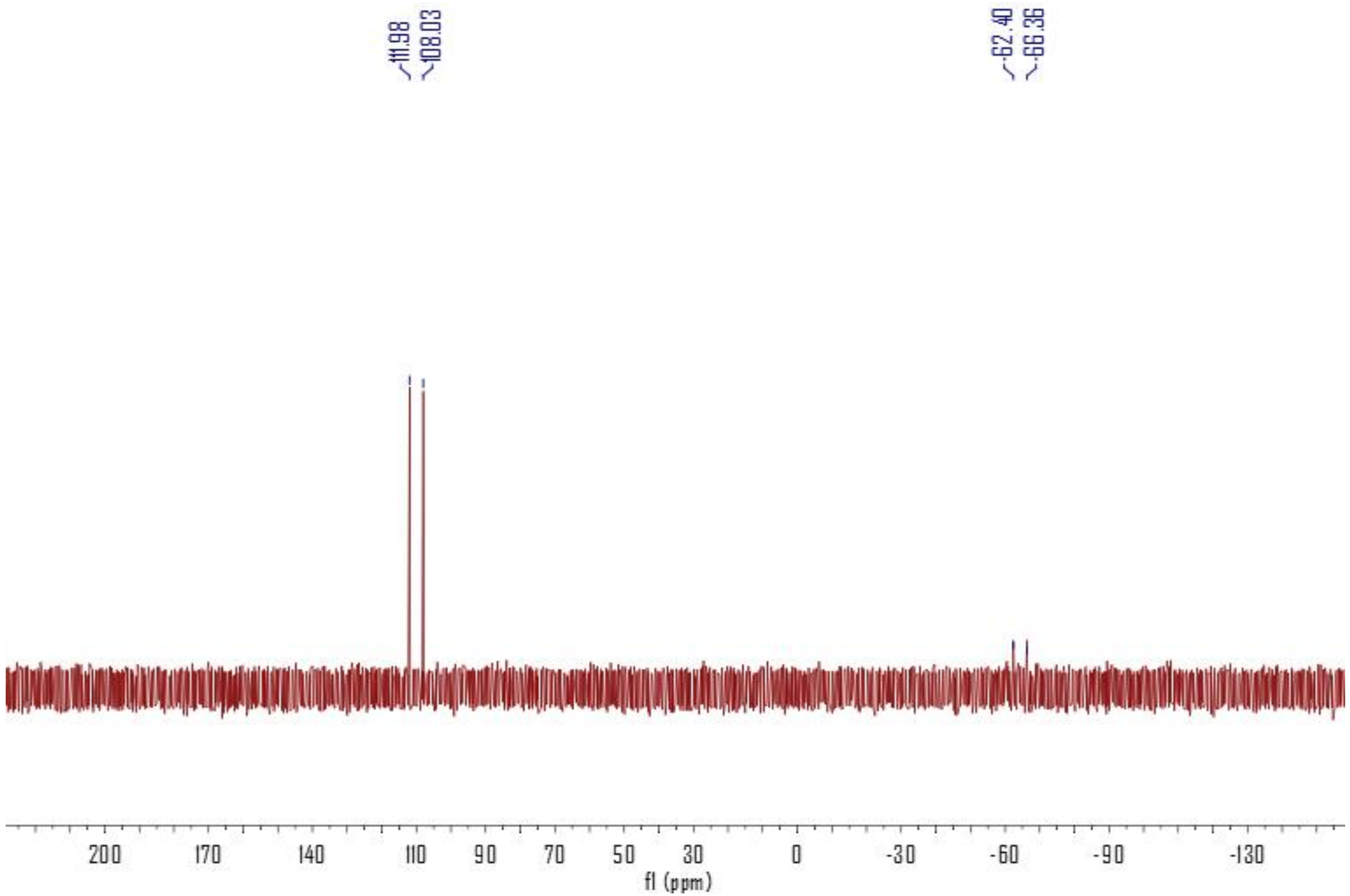

Supplementary Figure 29. $^{31}P\{^{1}H\}$ NMR of **4** (162 MHz, C_6D_6, 25 ºC).

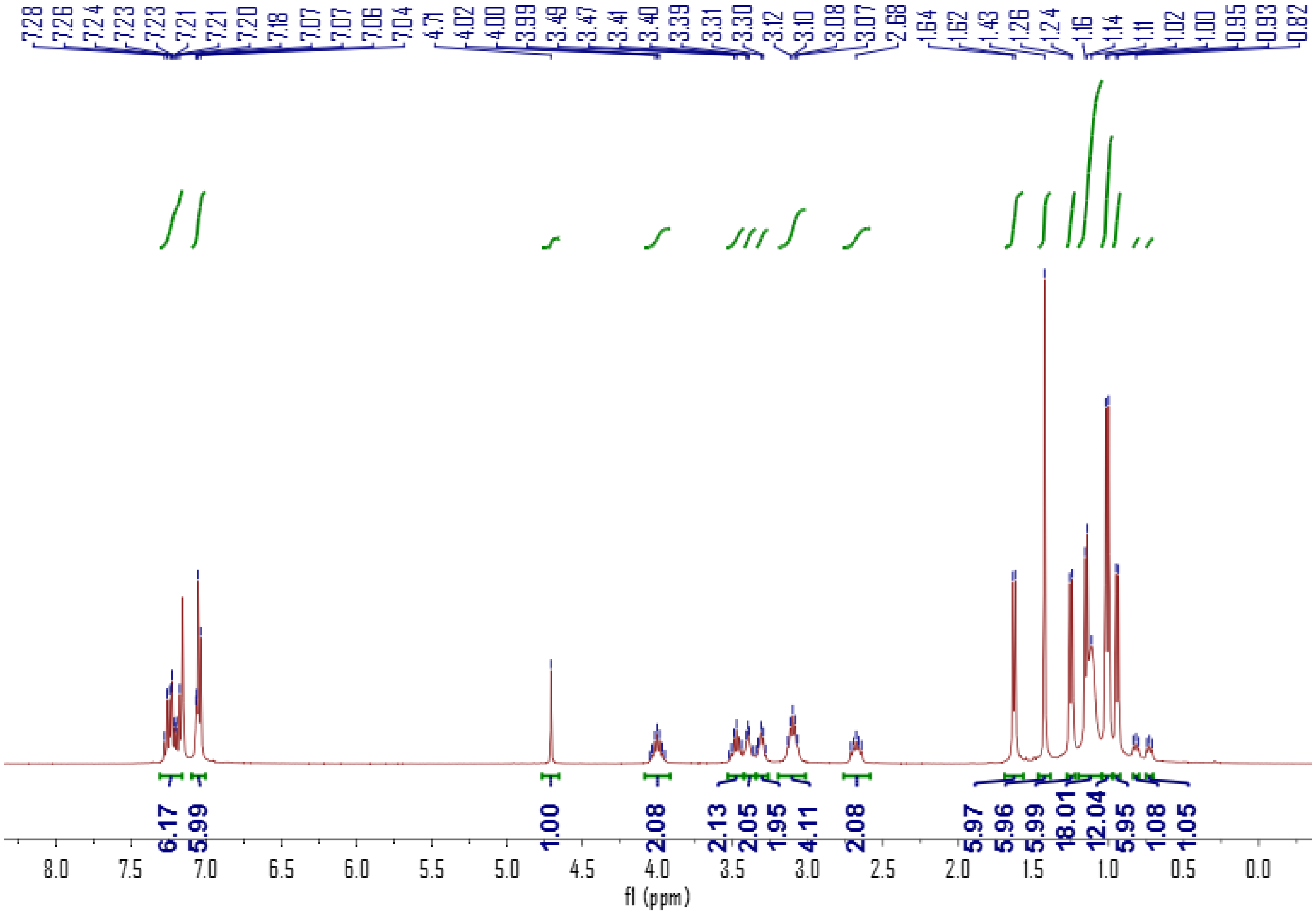

Supplementary Figure 30. ^{1}H NMR of **5** (400 MHz, C_6D_6, 25 ºC).

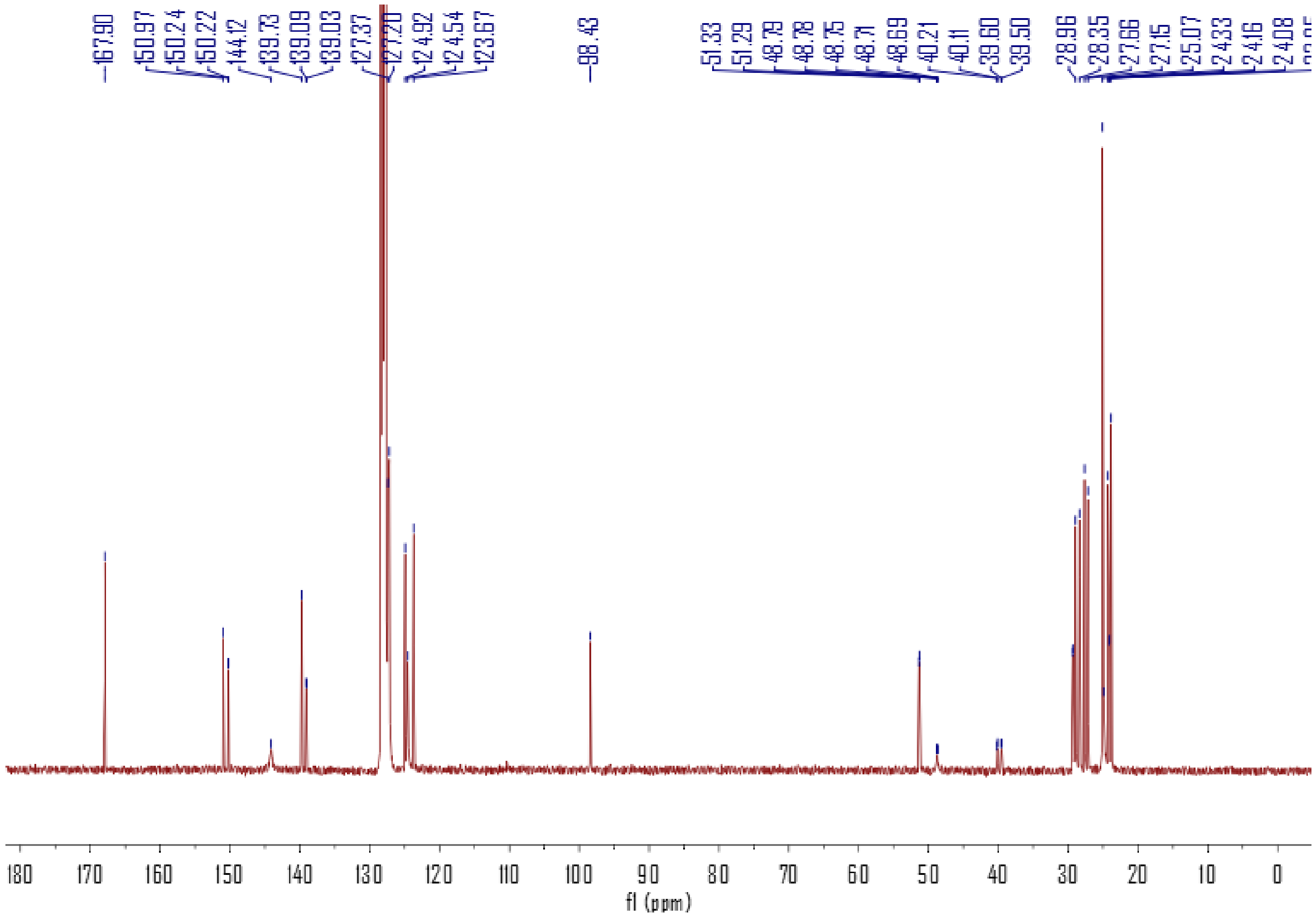

Supplementary Figure 31. $^{13}C\{^{1}H\}$ NMR of **5** (100 MHz, C_6D_6, 25 ºC).

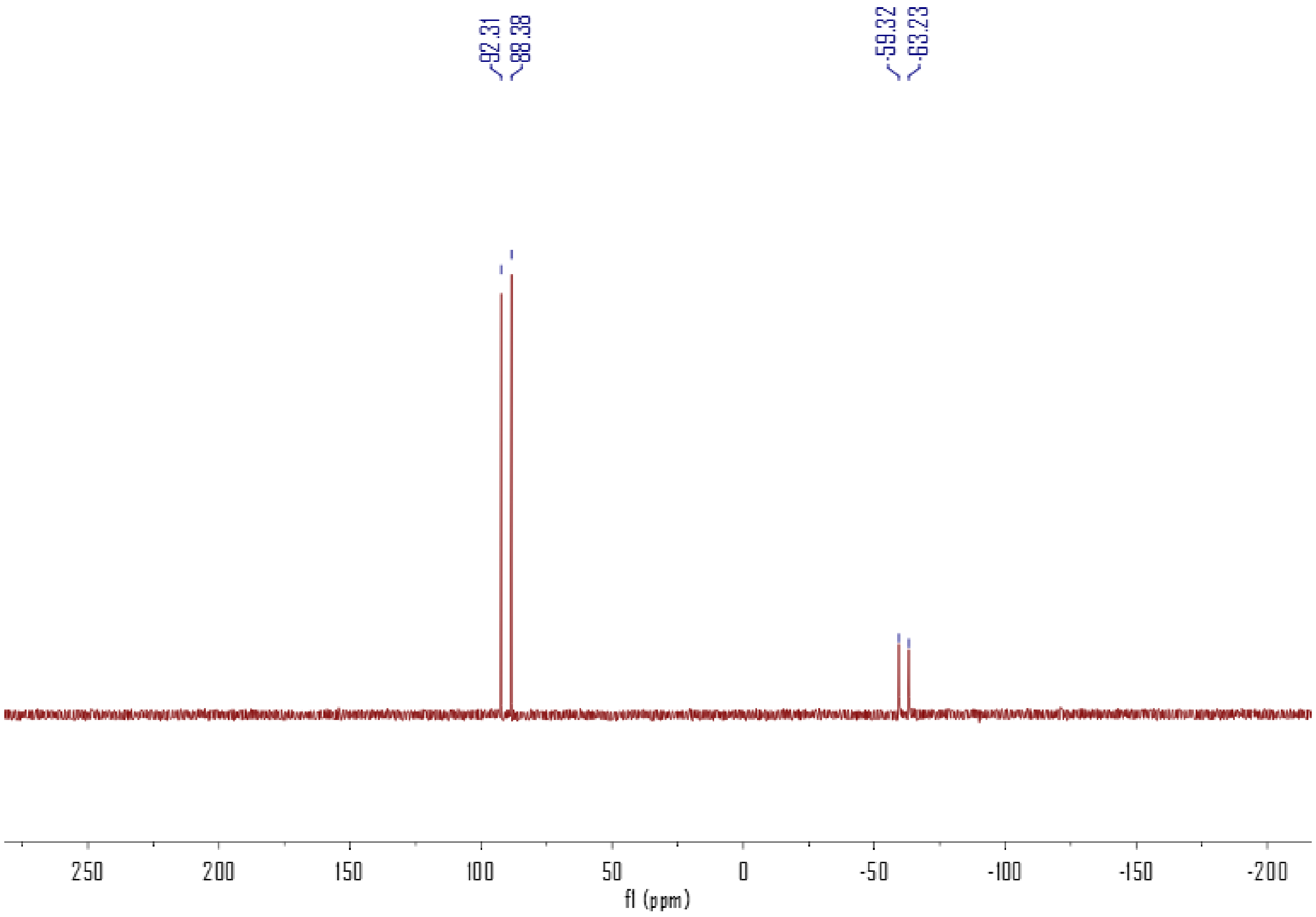

Supplementary Figure 32. $^{31}P\{^{1}H\}$ NMR of **5** (162 MHz, C_6D_6, 25 ºC).

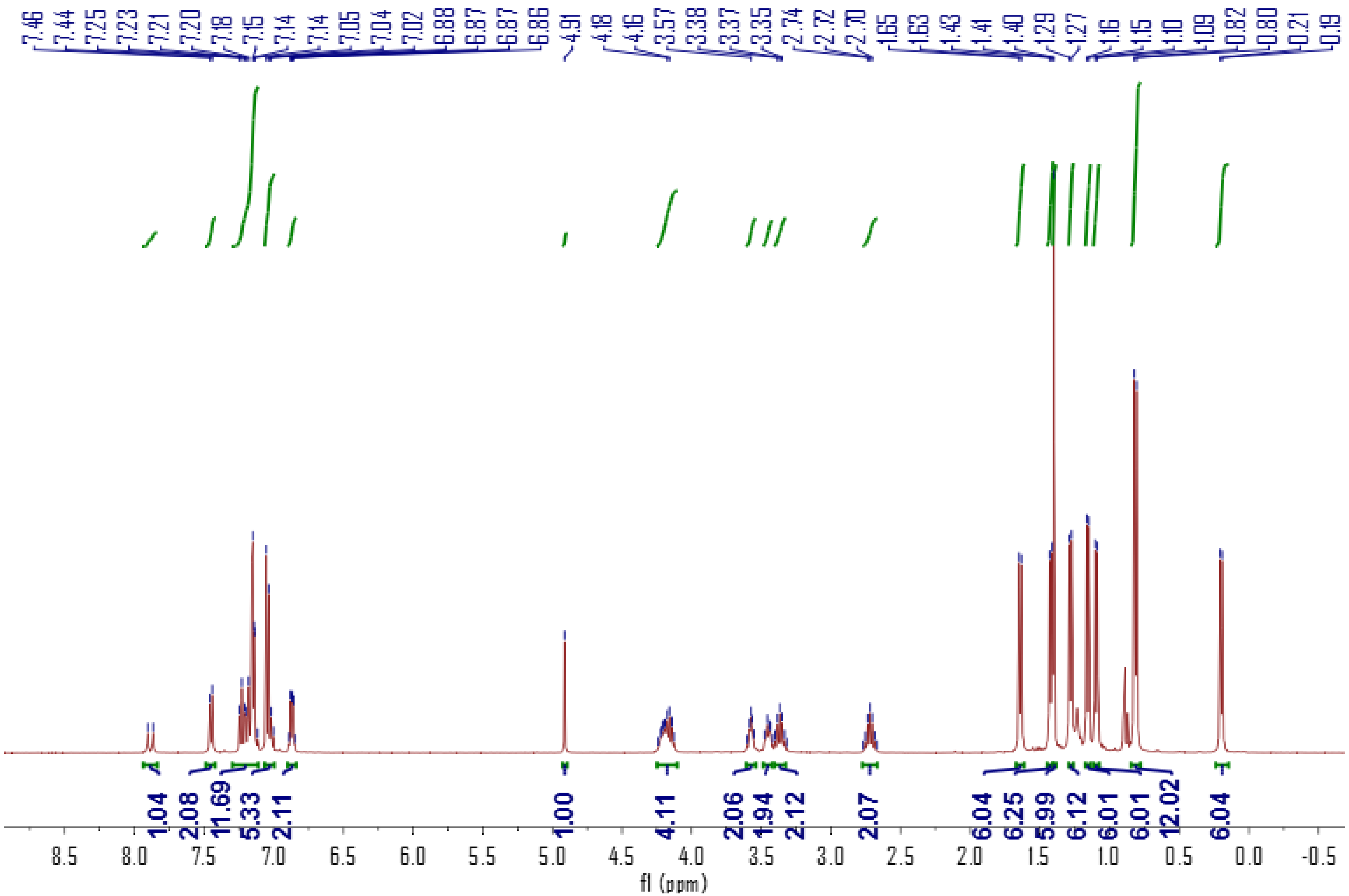

Supplementary Figure 33. ^{1}H NMR of **6** (400 MHz, C_6D_6, 25 ºC).

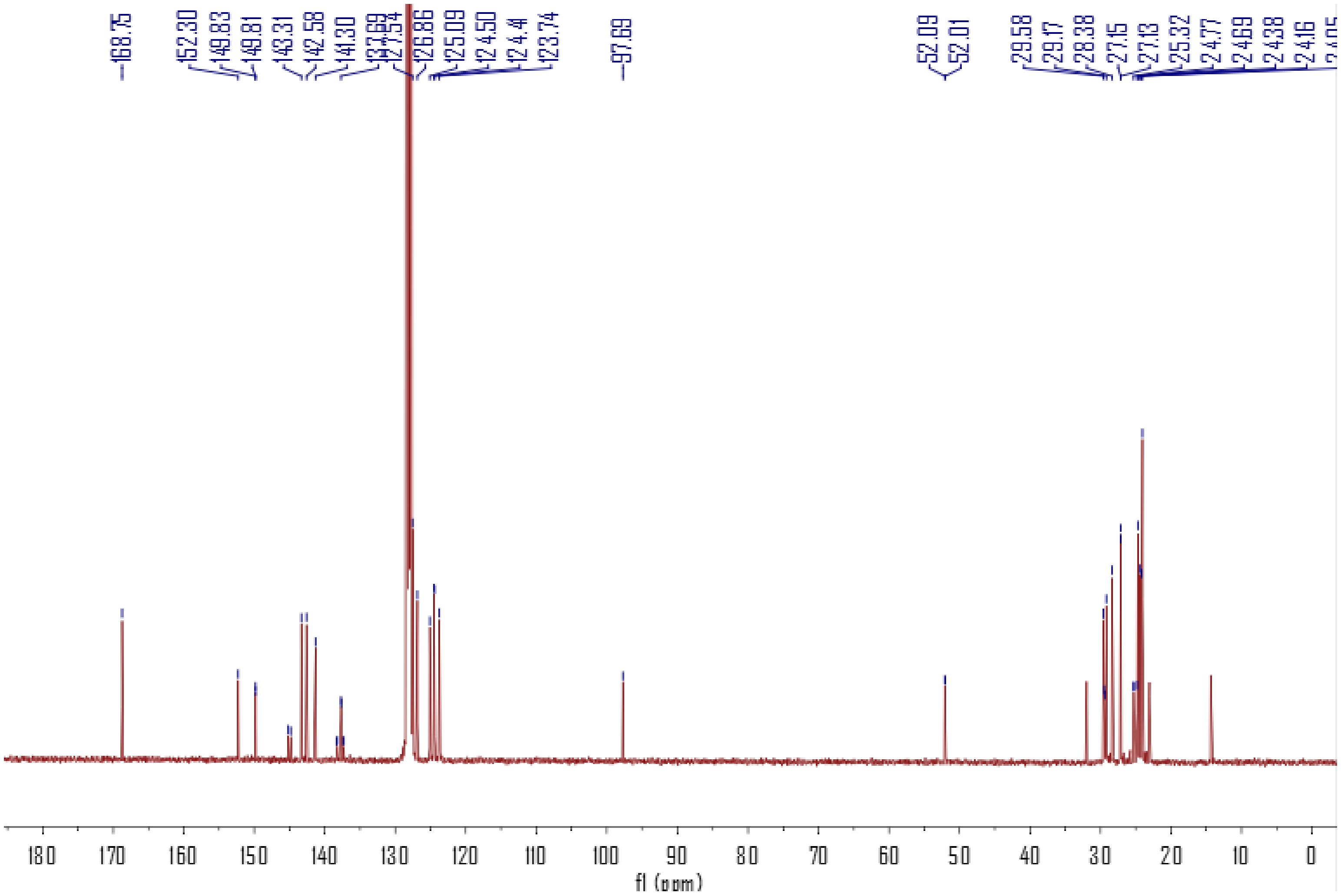

Supplementary Figure 34. $^{13}C\{^{1}H\}$ NMR of **6** (100 MHz, C_6D_6, 25 ºC).

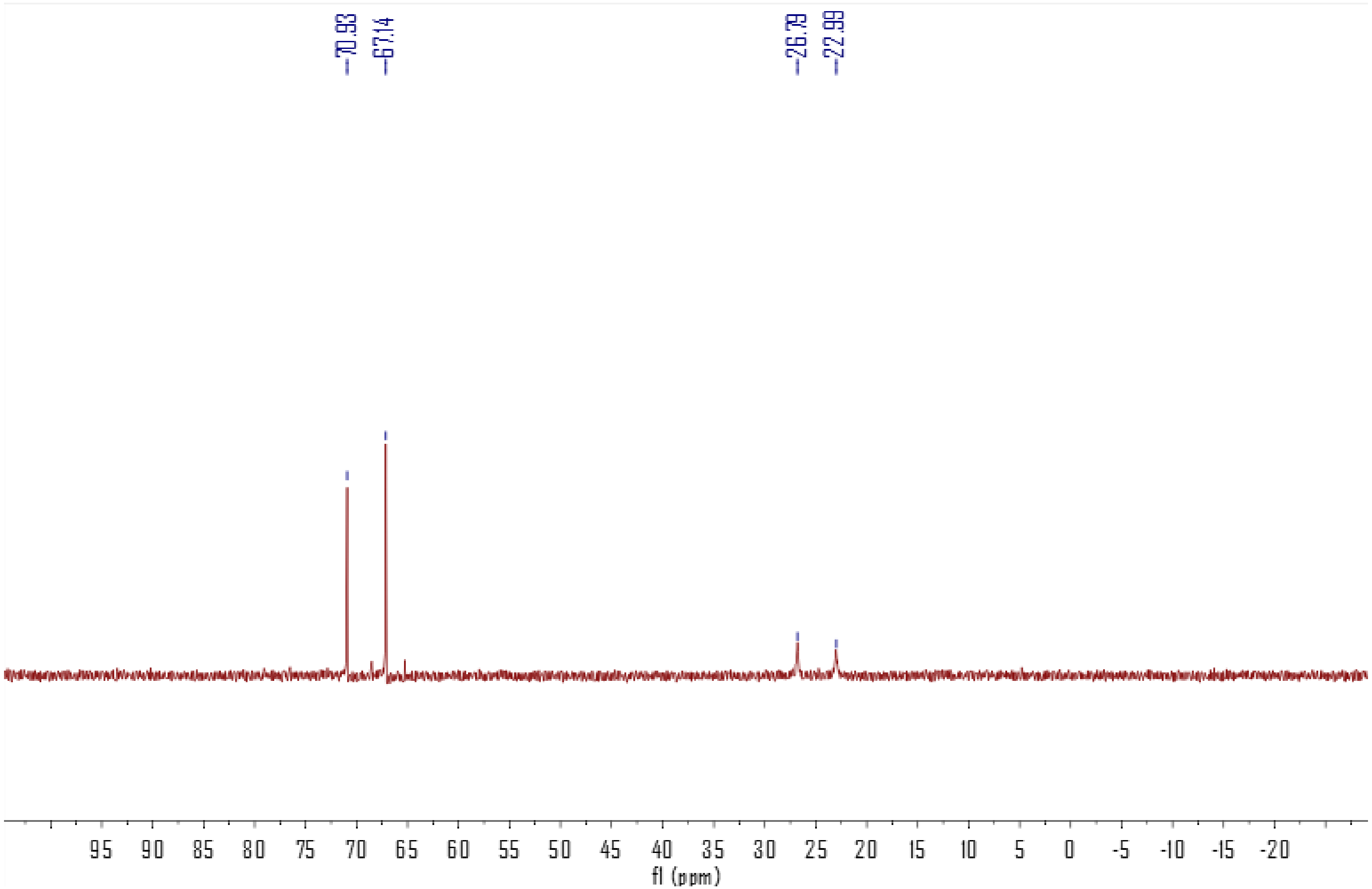

Supplementary Figure 35. $^{31}P\{^{1}H\}$ NMR of **6** (162 MHz, C_6D_6, 25 ºC).

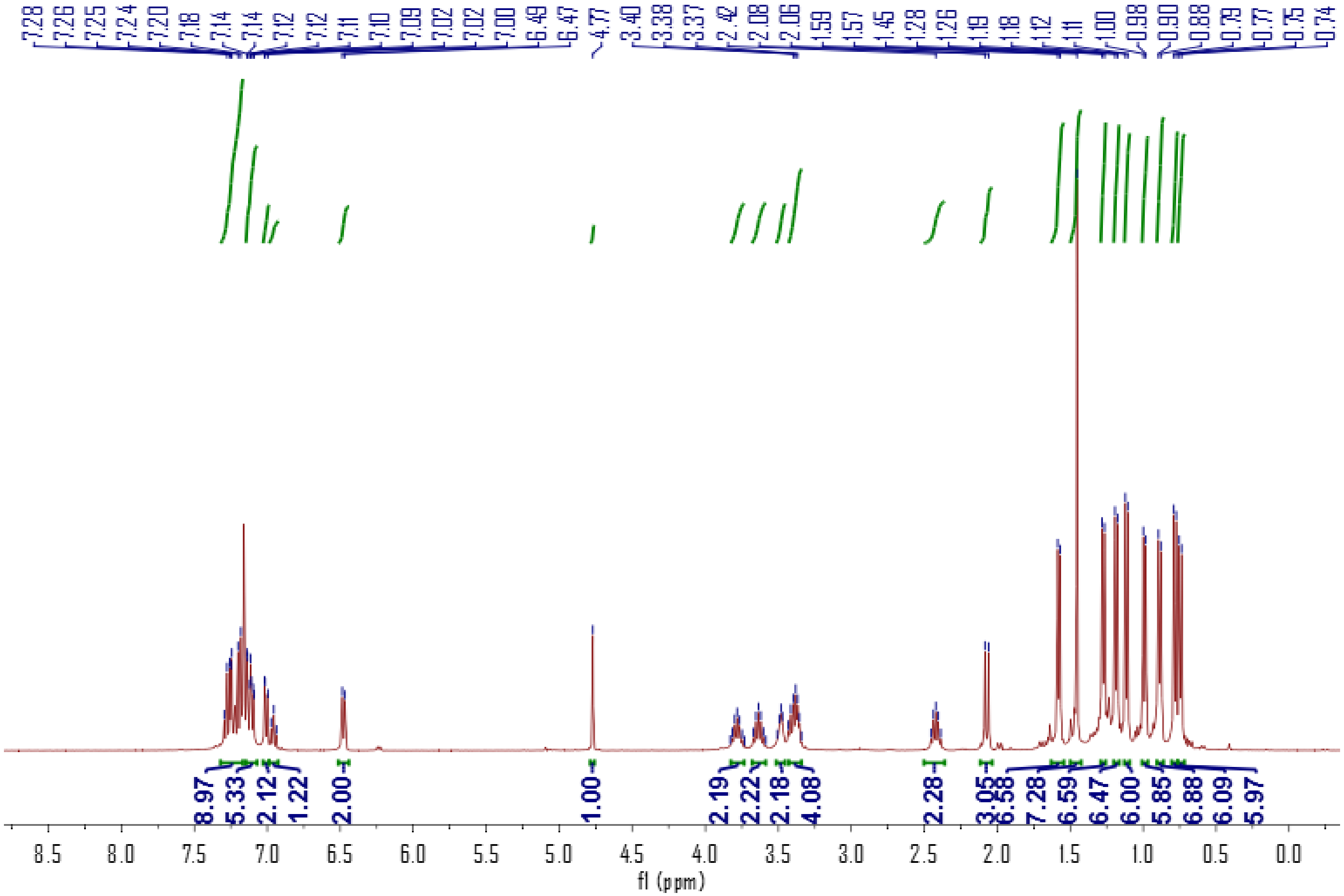

Supplementary Figure 36. ^{1}H NMR of **7** (400 MHz, C_6D_6, 25 ºC).

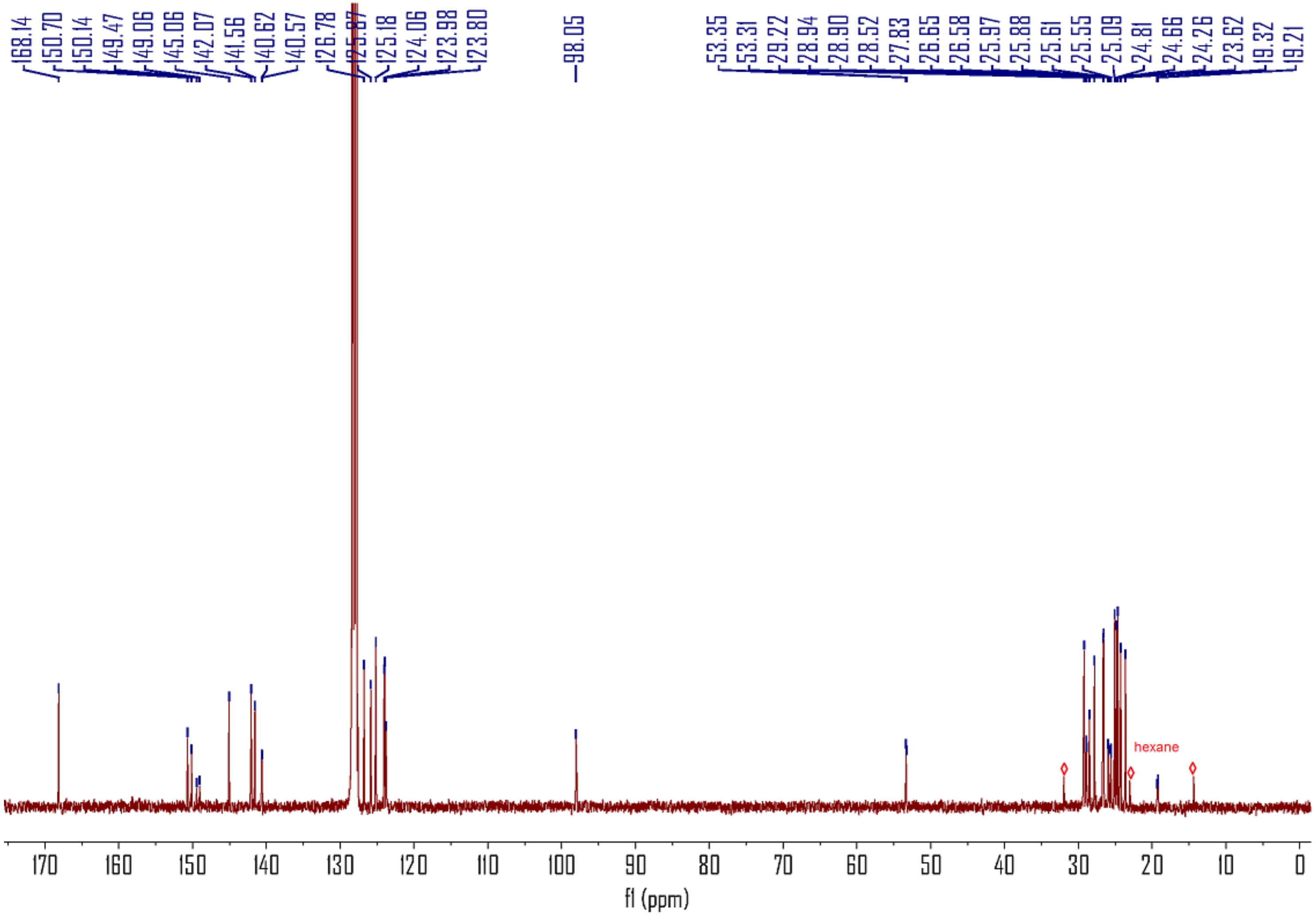

Supplementary Figure 37. $^{13}C\{^{1}H\}$ NMR of **7** (100 MHz, C_6D_6, 25 ºC).

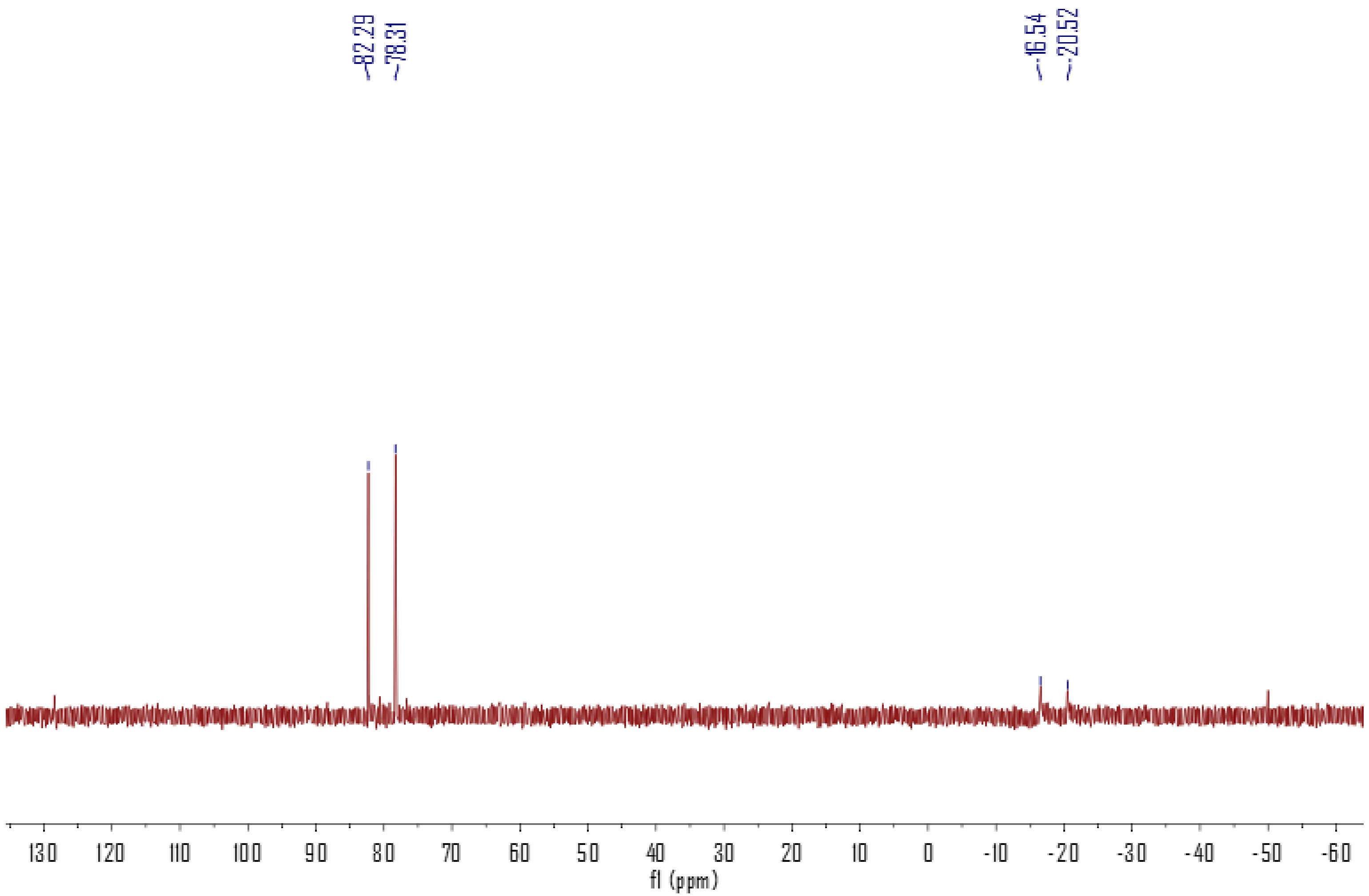

Supplementary Figure 38. $^{31}P\{^{1}H\}$ NMR of **7** (162 MHz, C_6D_6, 25 ºC).

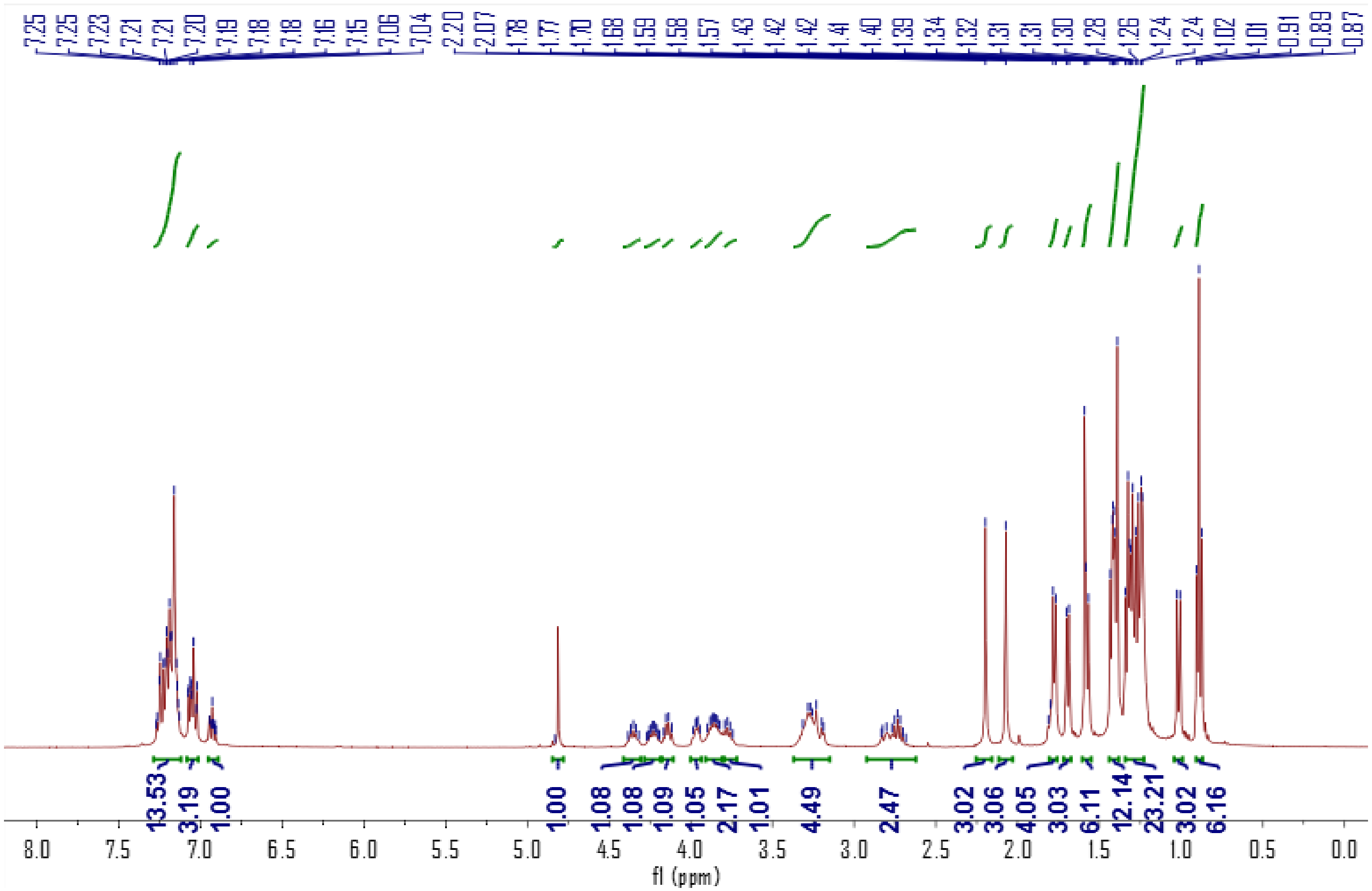

Supplementary Figure 39. ^{1}H NMR of **8** (400 MHz, C_6D_6, 25 °C).

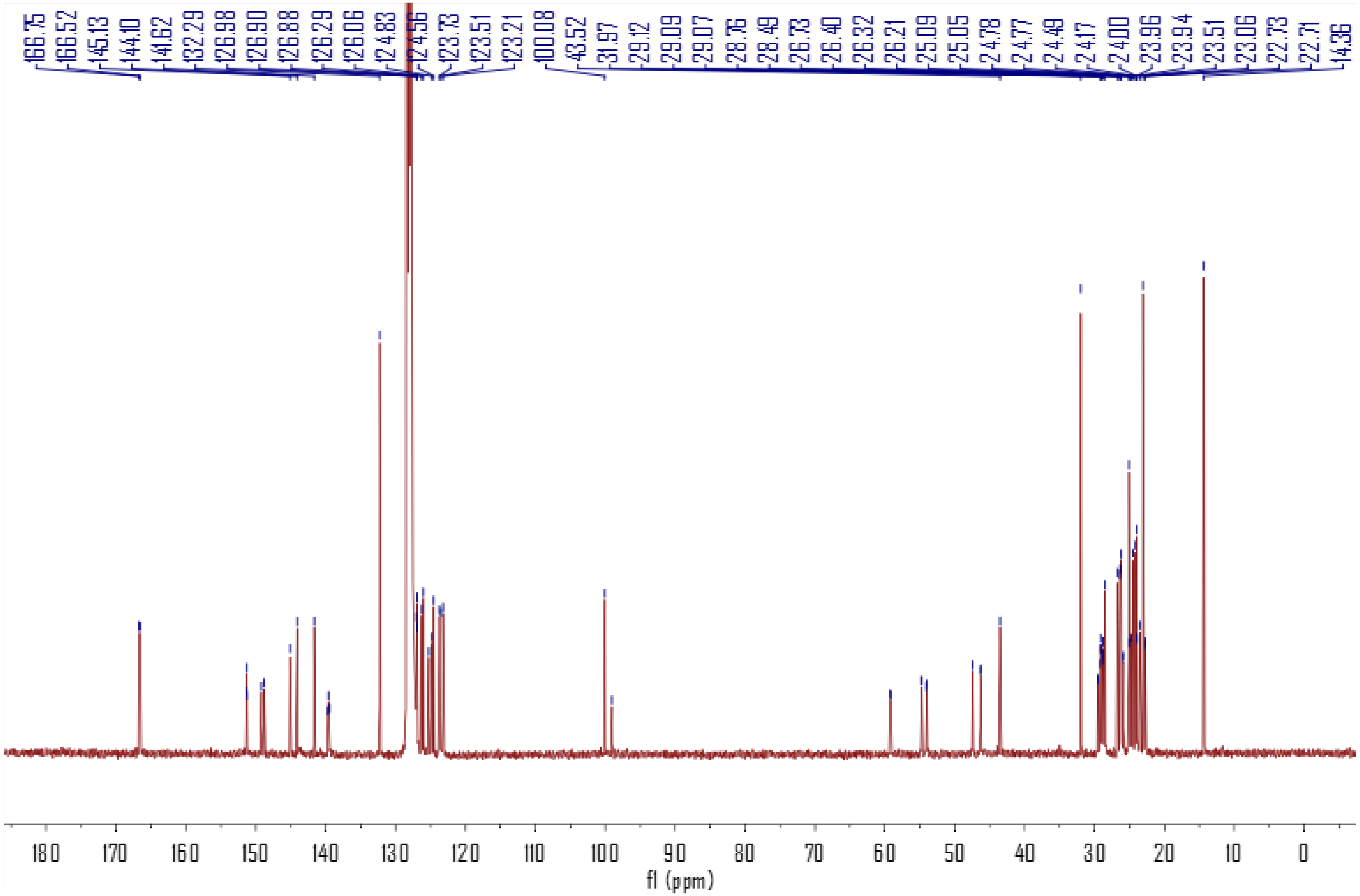

Supplementary Figure 40. $^{13}C\{^{1}H\}$ NMR of **8** (100 MHz, C_6D_6, 25 ºC).

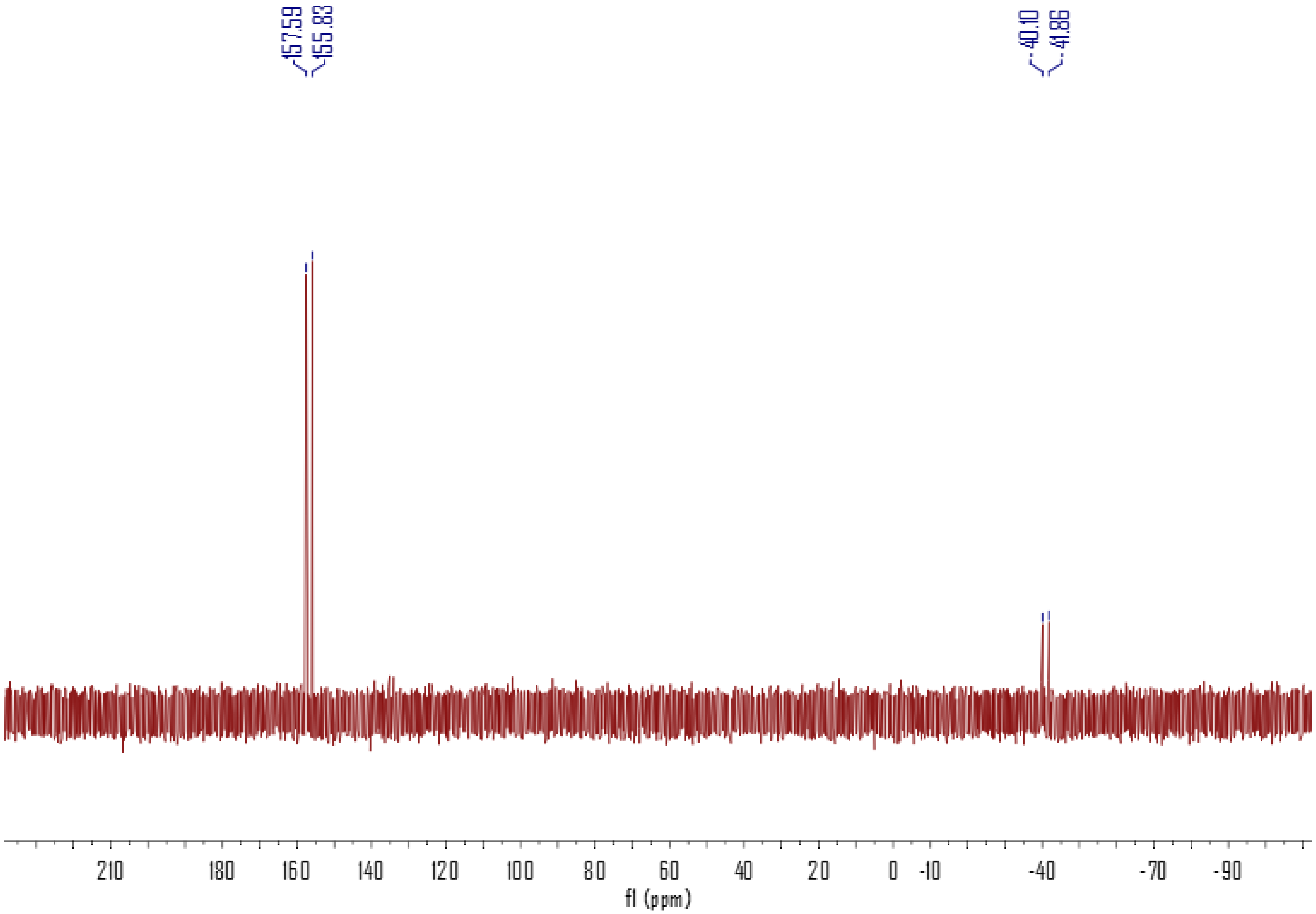

Supplementary Figure 41. $^{31}P\{^{1}H\}$ NMR of **8** (162 MHz, C_6D_6, 25 ºC).

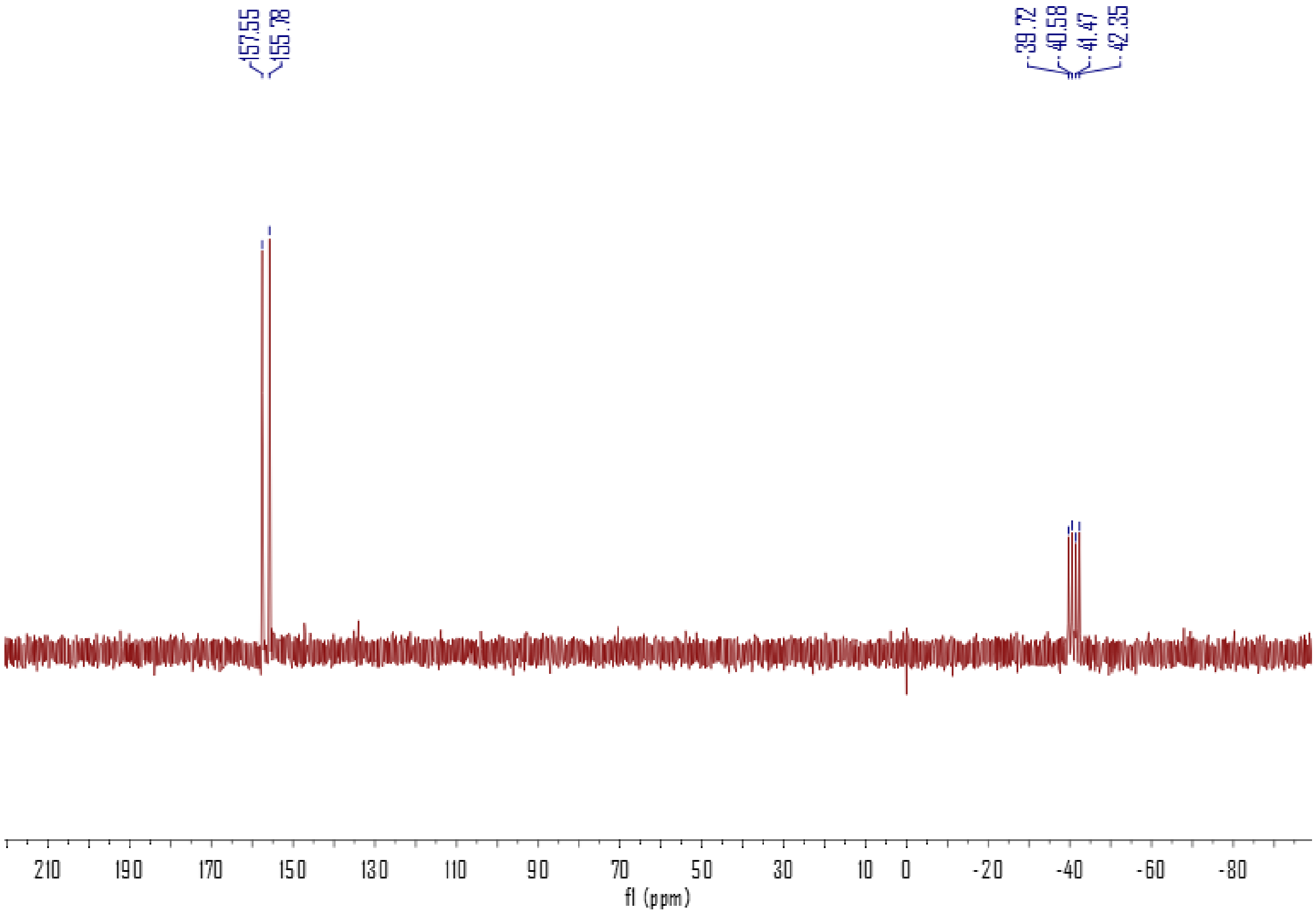

Supplementary Figure 42. ^{31}P NMR of **8** (162 MHz, C_6D_6, 25 °C).

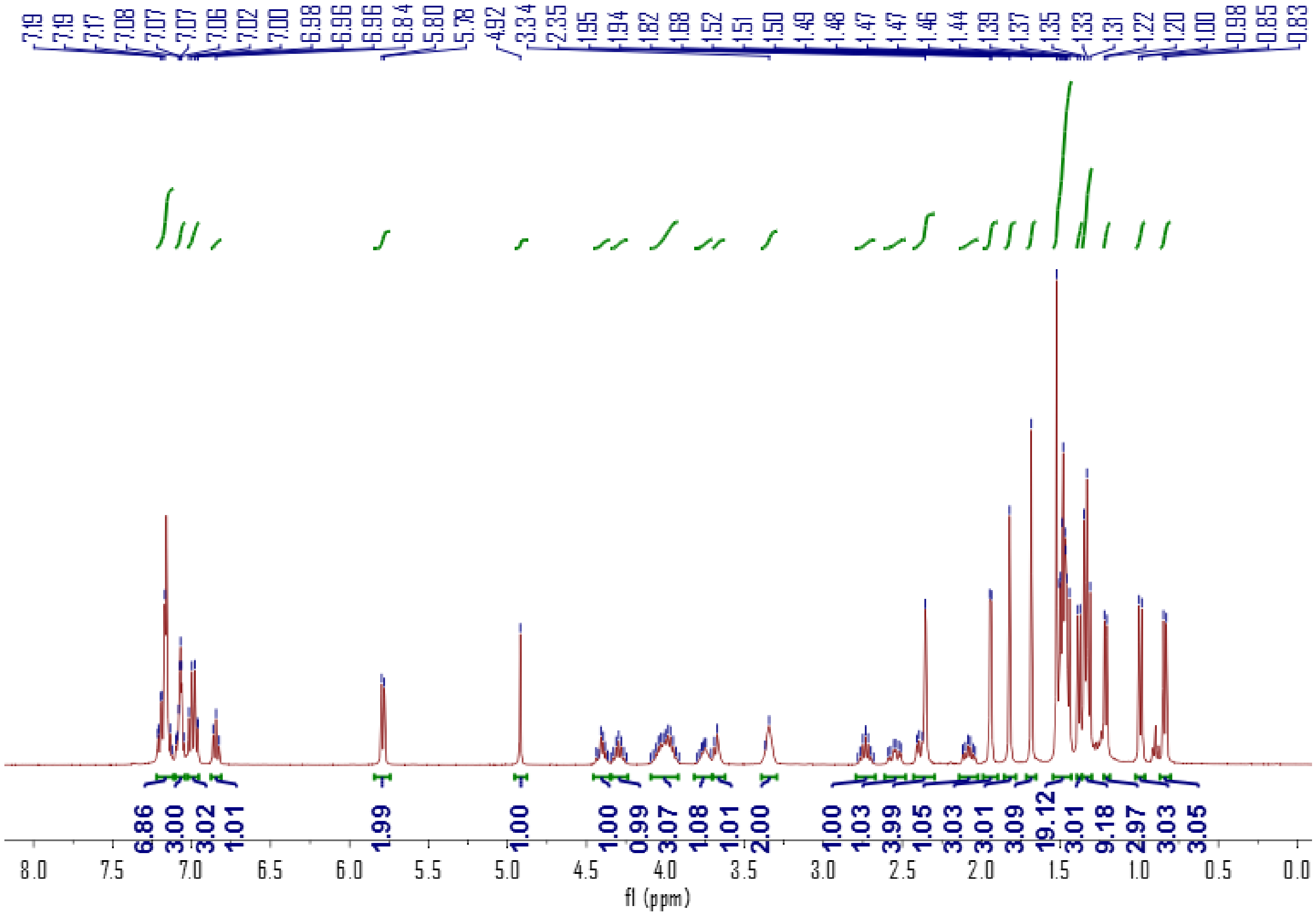

Supplementary Figure 43. ^{1}H NMR of **9** (400 MHz, C_6D_6, 25 ºC).

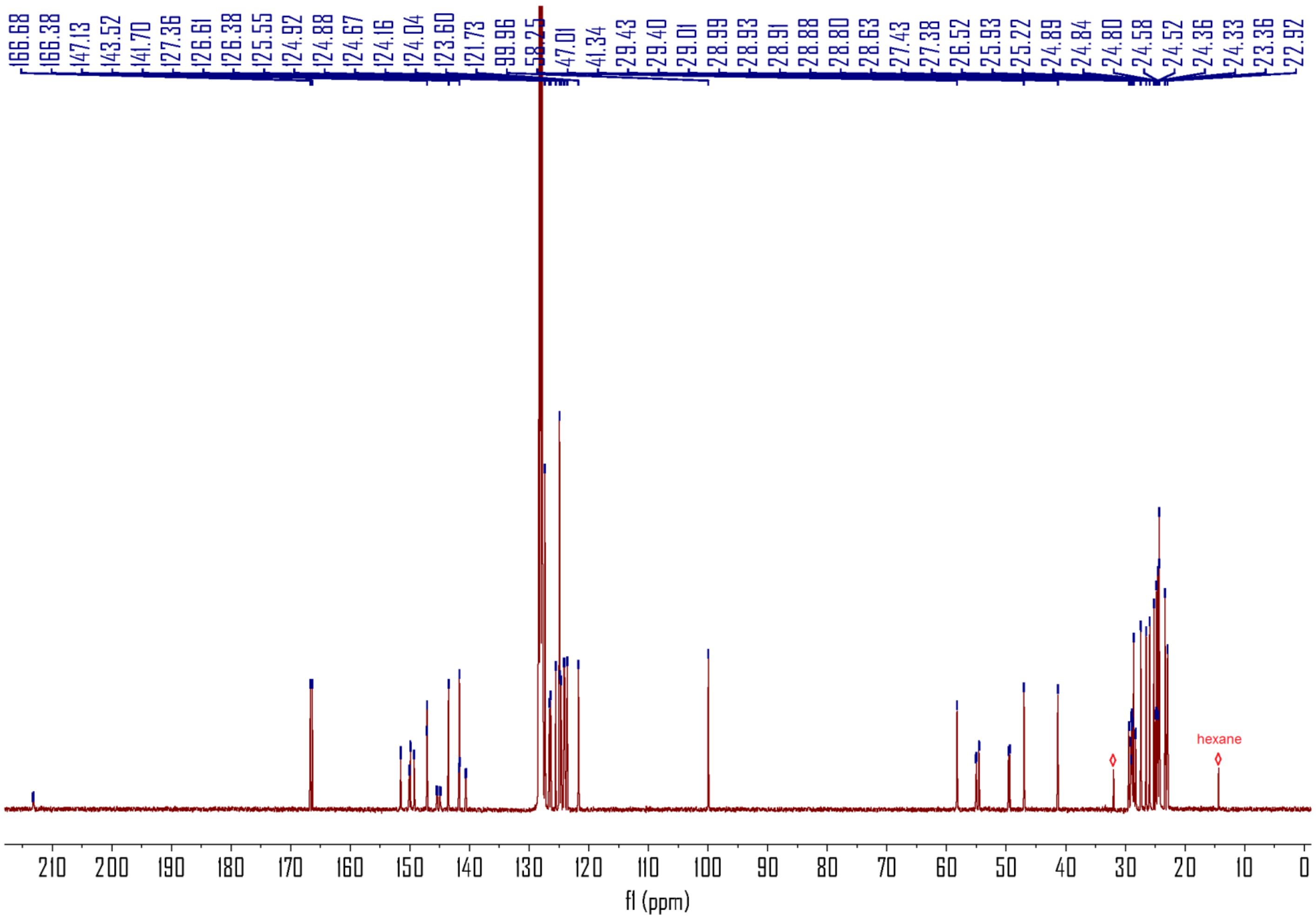

Supplementary Figure 44. $^{13}C\{^{1}H\}$ NMR of **9** (100 MHz, C_6D_6, 25 ºC).

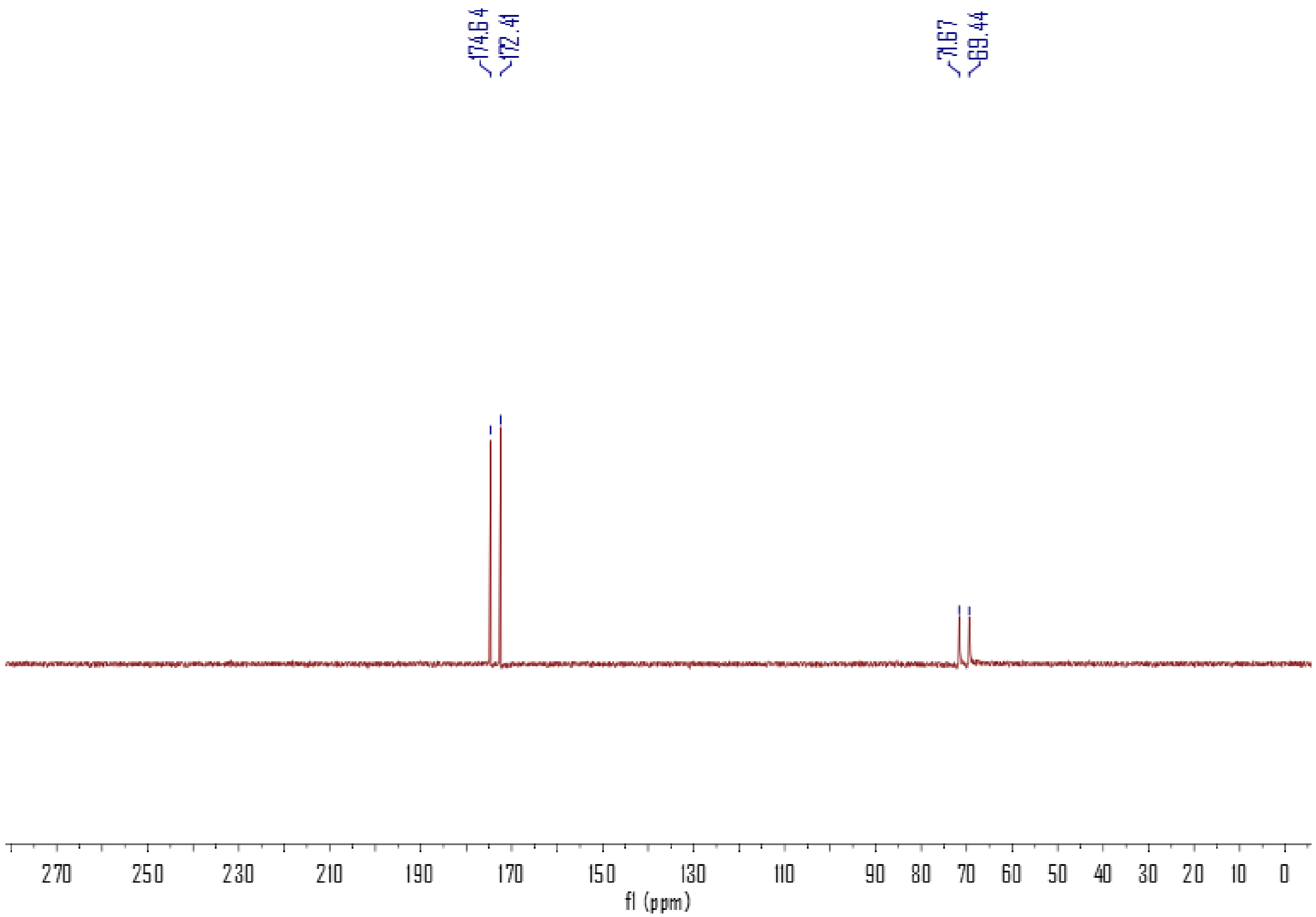

Supplementary Figure 45. $^{31}P\{^{1}H\}$ NMR of **9** (162 MHz, C_6D_6, 25 ºC).

Kinetic study on the reaction of complex 1 with phenylacetylene

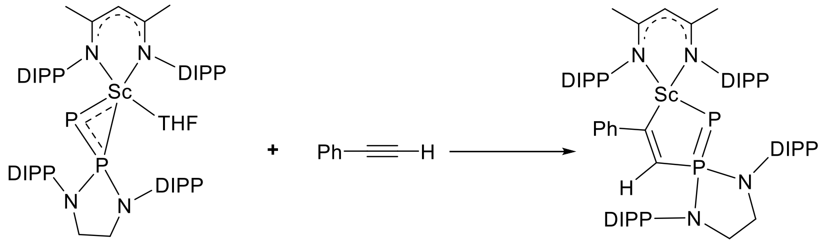

The initial concentrations of complex **1** and phenylacetylene were held constant at 0.0104 M for each run, the concentrations of THF or reaction temperatures were varied. The procedure used for a typical run follows: In a glovebox, a toluene-d_8 solution of complex **1** and the internal standard mesitylene (7.31 mg (7.5 μmol) of **1** and 0.30 mg (2.5 μmol) of mesitylene in 310 mg of toluene-d_8), a toluene-d_8 solution of phenylacetylene (0.77 mg, 7.5 μmol in 249 mg of toluene-d_8) and a toluene-d_8 solution of THF (27.04 mg, 375 μmol in 173 mg of toluene-d_8) were weighed into vials and cooled to -30 °C, respectively. The solutions were rapidly transferred to a precooled J. Young NMR tube. The NMR tube was immediately taken out of the glovebox and placed in a dry ice/ethanol cold bath. The NMR tube was taken out from the cold bath before being inserted into the probe of an Agilent 600 MHz spectrometer, which had been previously set to the target temperature. Data were collected using four scans per time interval with a 10 s delay to ensure accurate integration. The concentrations of complex **1** ([**1**]) were measured from the areas of the signals (MeC(N)C*H*)) at 4.62 ppm standardized to the areas of the signals of mesitylene (Ph*H*) at 6.65 ppm.

1. The reaction of complex **1** with one equiv of alkyne in the presence of 50 equiv of THF in toluene-d_8 at -10 °C was monitored by ^{1}H NMR spectroscopy, which showed the reaction rate is first-order in complex **1** and first-order in alkyne (as a large excess of THF was used, the THF concentration can be regarded as a constant through the reaction proceeding). As the concentration of **1** is same as that of alkyne (complex **1** reacts with alkyne quantitatively), the reaction rate can be simplified to second-order in complex **1** (Supplementary Equation 1).

$$\frac{-d[\mathbf{1}]}{d[t]} = \frac{d[P]}{d[t]} = k_{obs}[\mathbf{1}]\,[alkyne] + C \quad = k_{obs}[\mathbf{1}]^2 + C \qquad (1)$$

C is a constant.

Transposition available to do the indefinite integral equation.

$$-\int \frac{d[\mathbf{1}]}{[\mathbf{1}]^2} = \int k_{obs}\, d[t]$$

The obtained rate equation shows reaction time is positively correlated with the reciprocal of complex **1**'s concentration.

$$\frac{1}{[\mathbf{1}]} = k_{obs}\, t + C'$$

C' is a constant.

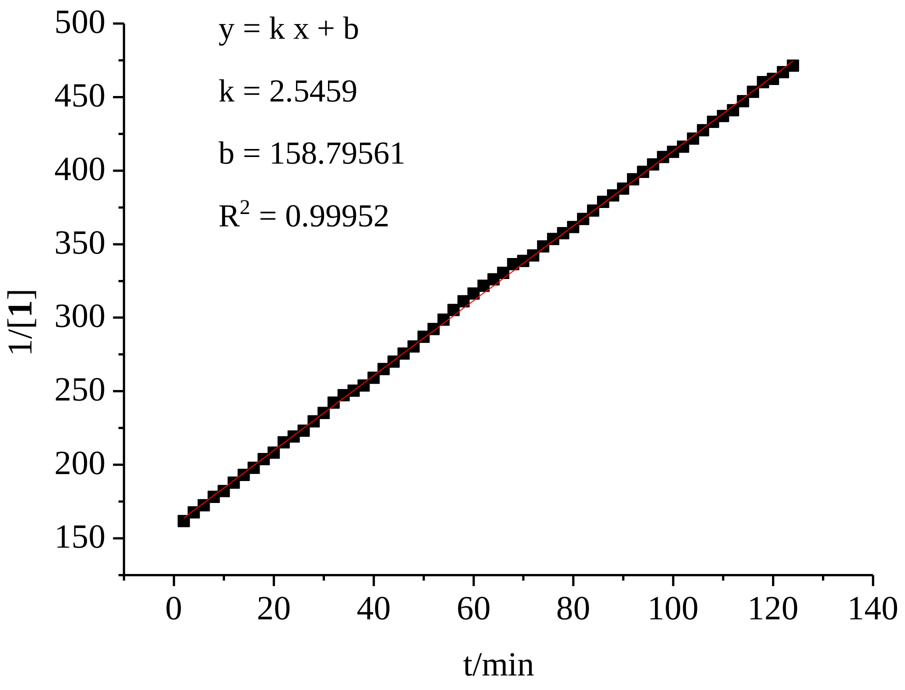

Supplementary Figure 46. Second-order plot with respect to complex **1** for the reaction of complex **1** with one equiv of alkyne in the presence of 50 equiv of THF at -10 °C in toluene-d_8.

2. The reactions of complex **1** with one equiv of phenylacetylene in toluene-d_8 at seven different THF concentrations (the initial [THF]:[**1**] ratios vary from 20 to 80) were monitored by ^{1}H NMR spectroscopy, which showed the reaction rate is first-order

in $[THF]^{-1}$ (as a large excess of THF was used, the THF concentration can be regarded as a constant through the reaction proceeding). Therefore, the reaction rate can be expressed as Supplementary Equation 2.

$$\frac{-d[\mathbf{1}]}{d[t]} = \frac{d[P]}{d[t]} = k'_{obs}[\mathbf{1}][alkyne][THF]^{-1} + C$$

$$\frac{-d[\mathbf{1}]}{d[t]} = k'_{obs}[\mathbf{1}]^2[THF]^{-1} + C \qquad (2)$$

$$k'_{obs} = k_{obs}[THF]$$

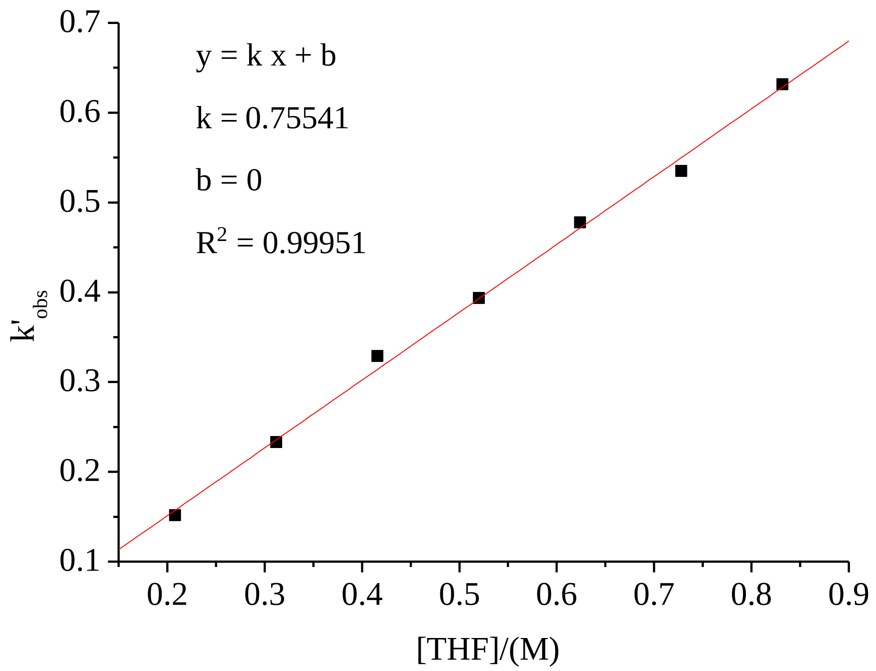

Supplementary Figure 47. Plot of k'_{obs} *vs* [THF].

3. The reactions of complex **1** with one equiv phenylacetylene in the presence of 75 equiv of THF in toluene-d_8 at six different temperatures between -18 and 2 °C were monitored by ^{1}H NMR spectroscopy. The reactions all fit with second-order in complex **1** (Supplementary Figure 48(a-f)), and an Eyring analysis provided activation parameters of $\Delta H^{\ddagger} = 15.0(4)$ *kcal mol*$^{-1}$, $\Delta S^{\ddagger} = 0(1)$ *cal mol*$^{-1}$ *K*$^{-1}$; $\Delta G^{\ddagger} = 15.0$ *kcal mol*$^{-1}$ *for 298.15 K* (Supplementary Figure 48g).

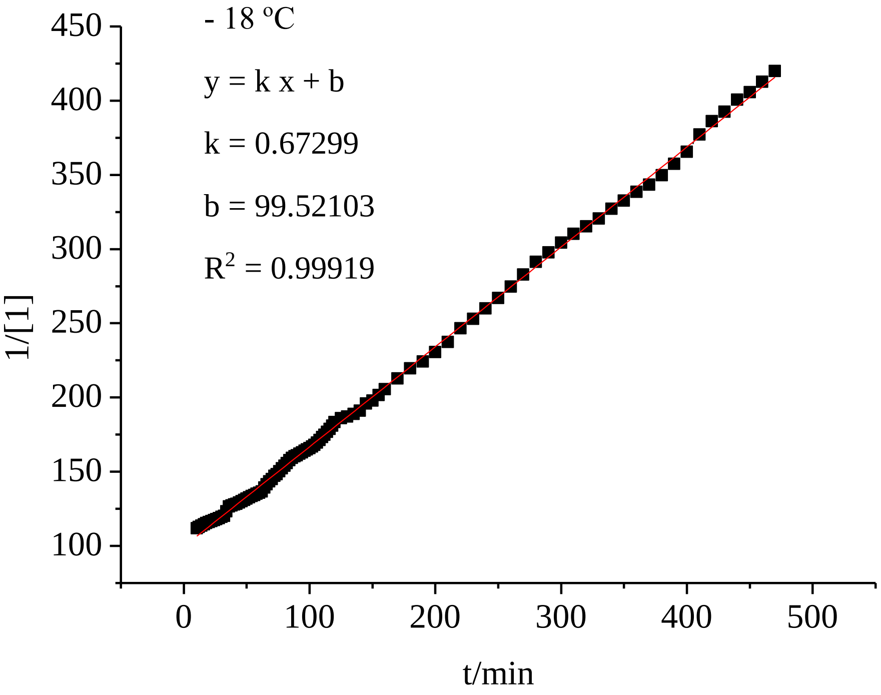
- 18 ºC
y = k x + b
k = 0.67299
b = 99.52103
R2 = 0.99919
1/[1]
t/min

(a)

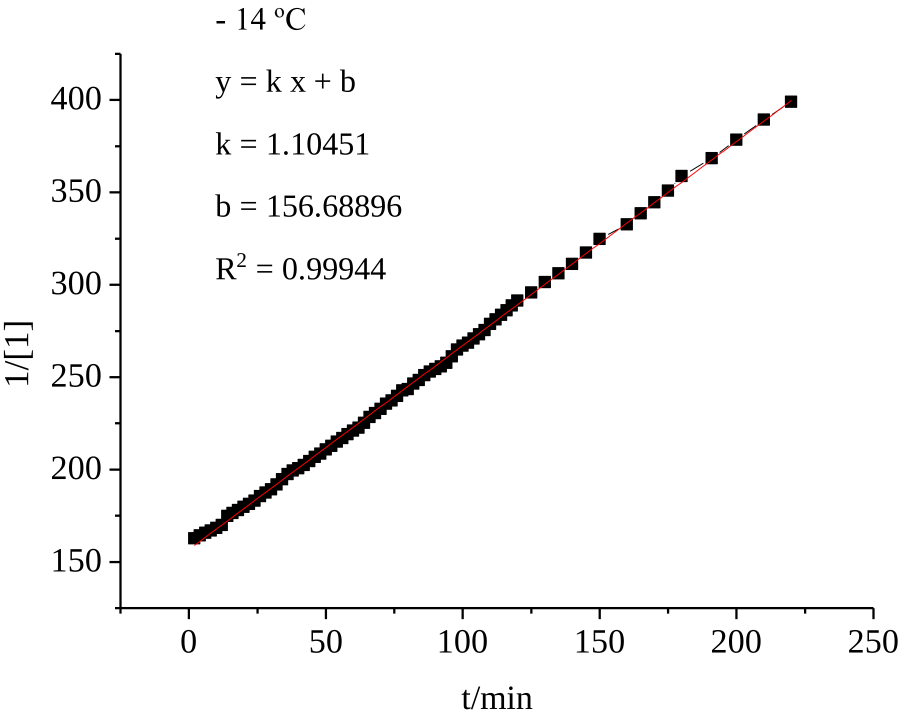
- 14 ºC
y = k x + b
k = 1.10451
b = 156.68896
R2 = 0.99944
1/[1]
t/min

(b)

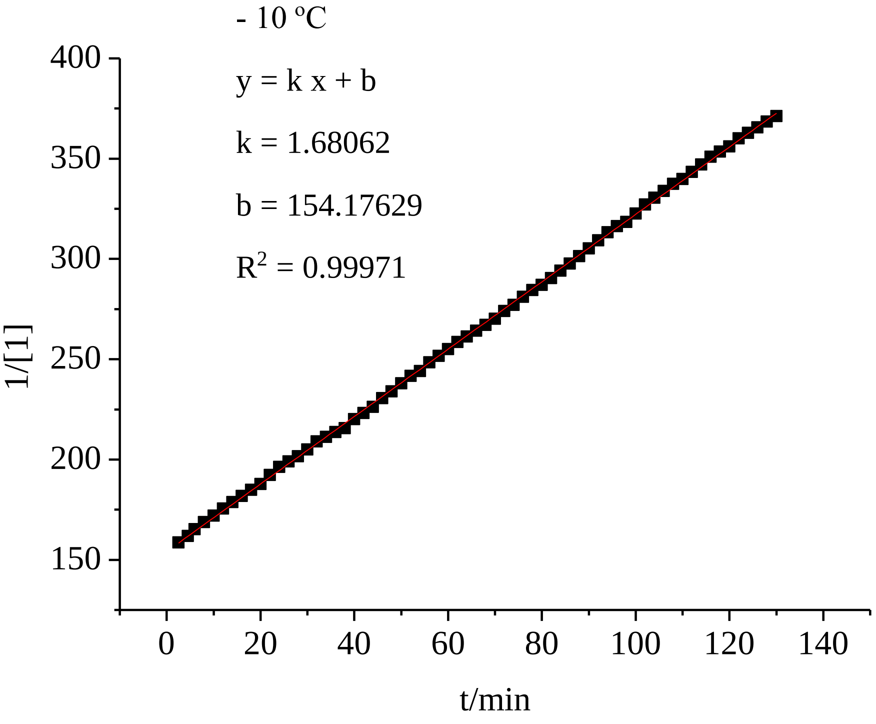
- 10 ºC
y = k x + b
k = 1.68062
b = 154.17629
R2 = 0.99971
1/[1]
t/min
400
350
300
250
200
150
0
20
40
60
80
100
120
140

(c)

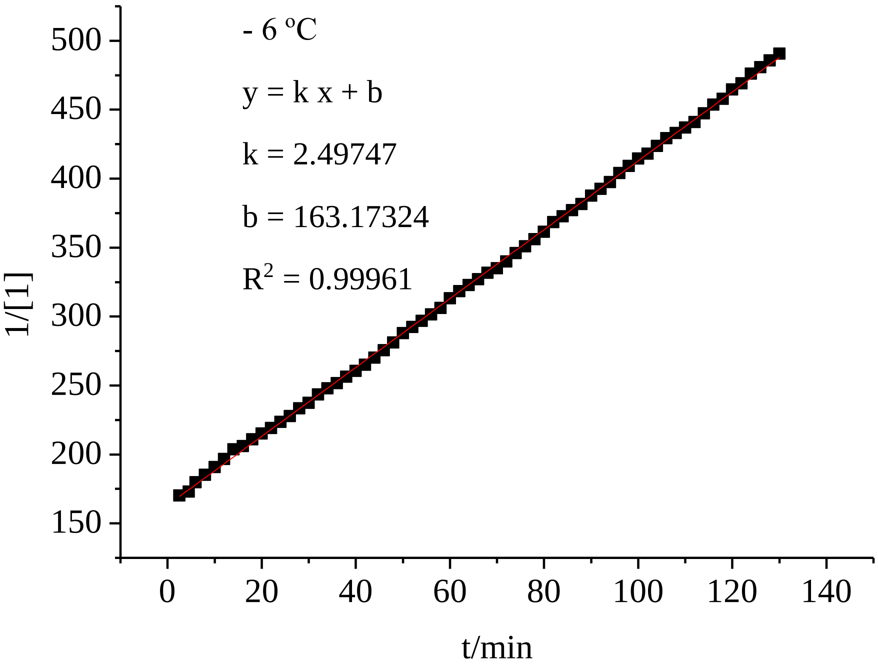
- 6 ºC
y = k x + b
k = 2.49747
b = 163.17324
R2 = 0.99961
1/[1]
t/min
500
450
400
350
300
250
200
150
0
20
40
60
80
100
120
140

(d)

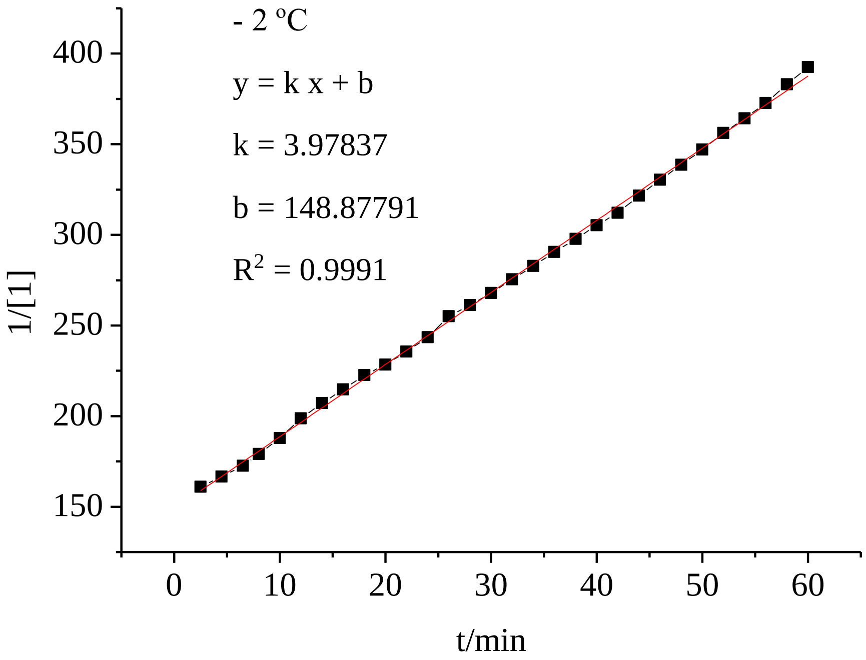
- 2 ºC
y = k x + b
k = 3.97837
b = 148.87791
R2 = 0.9991
1/[1]
400
350
300
250
200
150
0
10
20
30
40
50
60
t/min

(e)

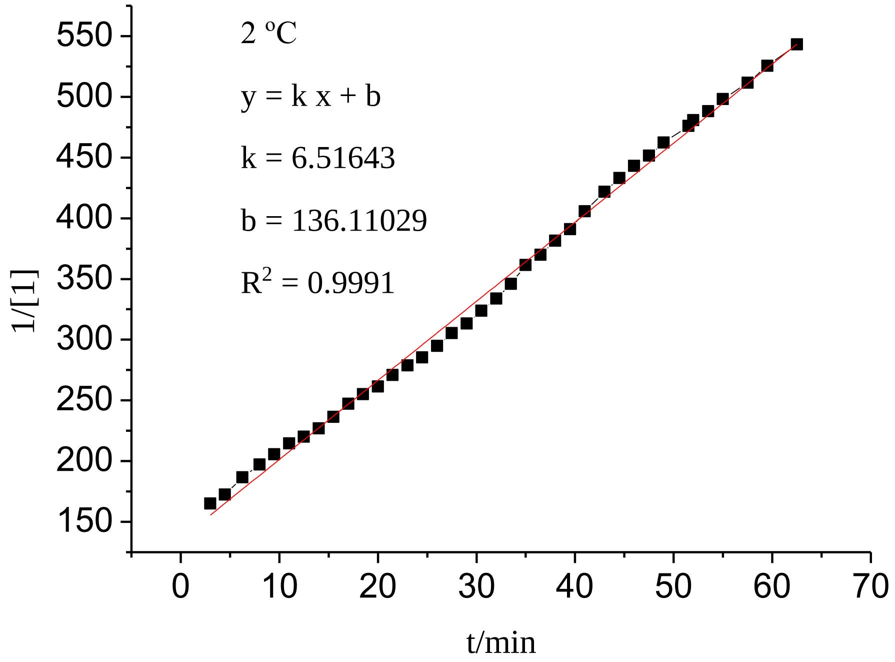
2 ºC
y = k x + b
k = 6.51643
b = 136.11029
R2 = 0.9991
1/[1]
550
500
450
400
350
300
250
200
150
0
10
20
30
40
50
60
70
t/min

(f)

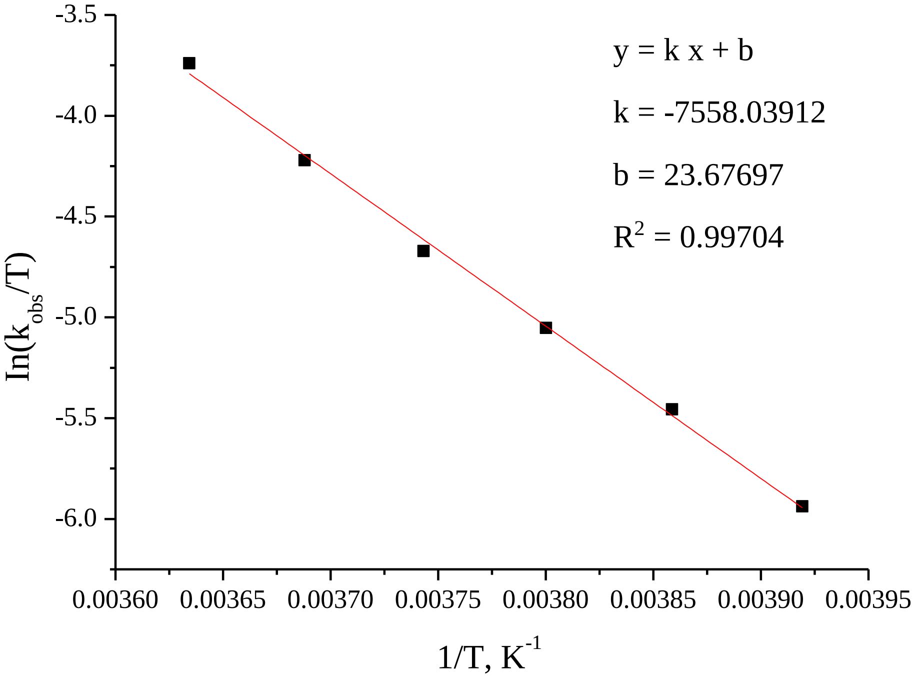

(g)

Supplementary Figure 48. (a-f): Second-order plots with respect to complex **1** for the reactions of complex **1** with one equiv of alkyne in the presence of 75 equiv of THF in toluene-d_8 at six different temperatures between -18 and 2 °C. (g): Eyring plot for the reactions of complex **1** with one equiv phenylacetylene in the presence of 75 equiv of THF. Activation parameters derived from linear regression analysis: $\Delta H^‡ = 15.0(4)$ kcal mol^{-1}, $\Delta S^‡ = 0(1)$ cal mol^{-1} K^{-1}; $\Delta G^‡ = 15.0$ kcal mol^{-1} for 298.15 K.

Computational details

Calculations were carried out with Gaussian09 [14] at the DFT level, with the hybrid functional B3PW91[15-17]. Scandium and silicon atoms were treated with small-core pseudopotentials from the Stuttgart group, with additional polarization orbitals[18,19]. The other atoms that were part of the systems (phosphorus, nitrogen, carbon, and hydrogen) were treated with the extended all electron Gaussian-Type 6-31G** Pople basis set[20]. No symmetry constraints were considered for the geometry optimizations that took as starting point the experimentally obtained geometries of both reagents and products. Analytical calculations of the vibrational frequencies confirmed that the structures obtained were the critical points involved in the reactive process, and also obtained the thermal corrections over the energies. Transition states obtained where connected with its respective intermediates with Intrinsic Reaction Coordinate (IRC) calculations. Bonding was studied doing Natural Bond Orbital analysis over the optimized structures, with NBO software[21]. All of the calculations of Fukui functions were conducted using the Multiwfn software[22].

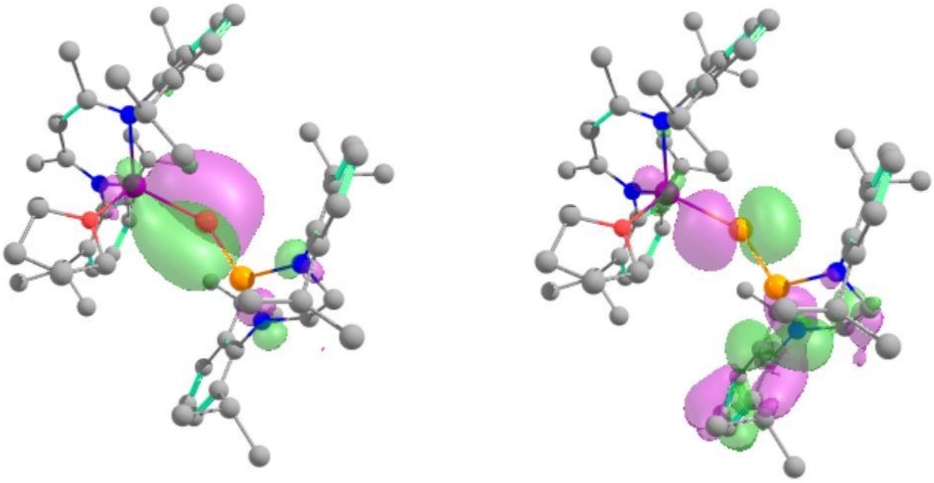

Supplementary Figure 49. HOMO-1 and HOMO-2 of complex **1**. The HOMO-1 and HOMO-2 present the interactions between the P_{α} and Sc. Atom colour code: purple, scandium; orange, phosphorus; red, oxygen; blue, nitrogen; grey, carbon.

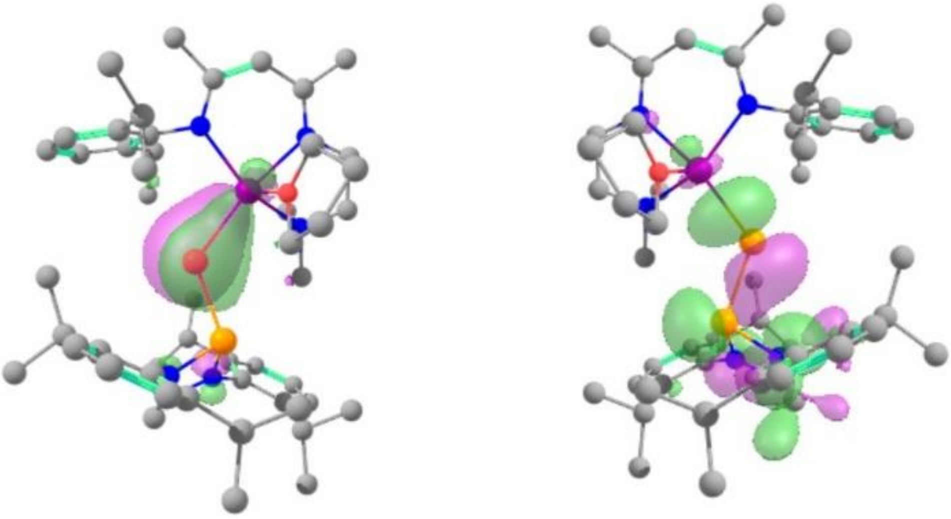

Supplementary Figure 50. HOMO-1 and HOMO-2 of complex **2**. The HOMO-1 and HOMO-2 present the interactions between the P_{α} and Sc. Atom colour code: purple, scandium; orange, phosphorus; red, oxygen; blue, nitrogen; grey, carbon.

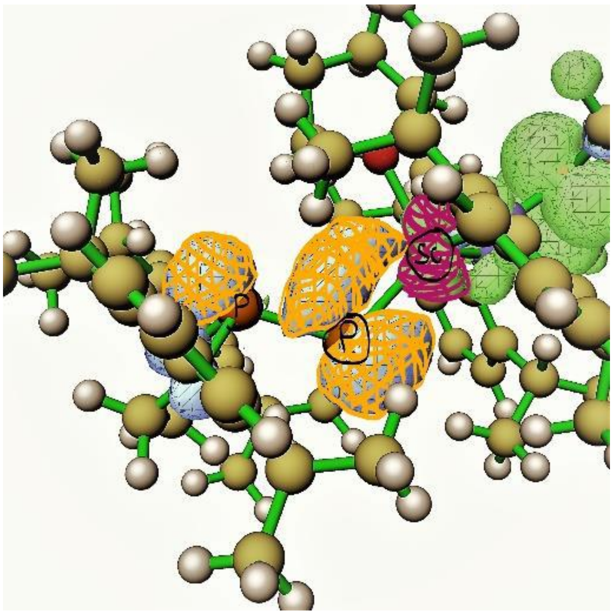

Supplementary Figure 51. Graphical representation of the Dual Descriptor Fukui indexes of complex **1**.

Supplementary Table 2. Hirsfield charges, Fukui descriptor for electrophilicity and nucleophilicity and dual Fukui descriptor of complex **1**

**Atom**	N	$N-1$	$N+1$	$f-$	$f+$	Δf
Sc	0.236511	0.289362	0.232974	0.052851	0.003537	0.007074
P_{α}	-0.429316	-0.207452	-0.457688	0.221864	0.028372	-0.193492
P_{β}	0.112698	0.151977	0.076607	0.129279	0.036091	-0.093188

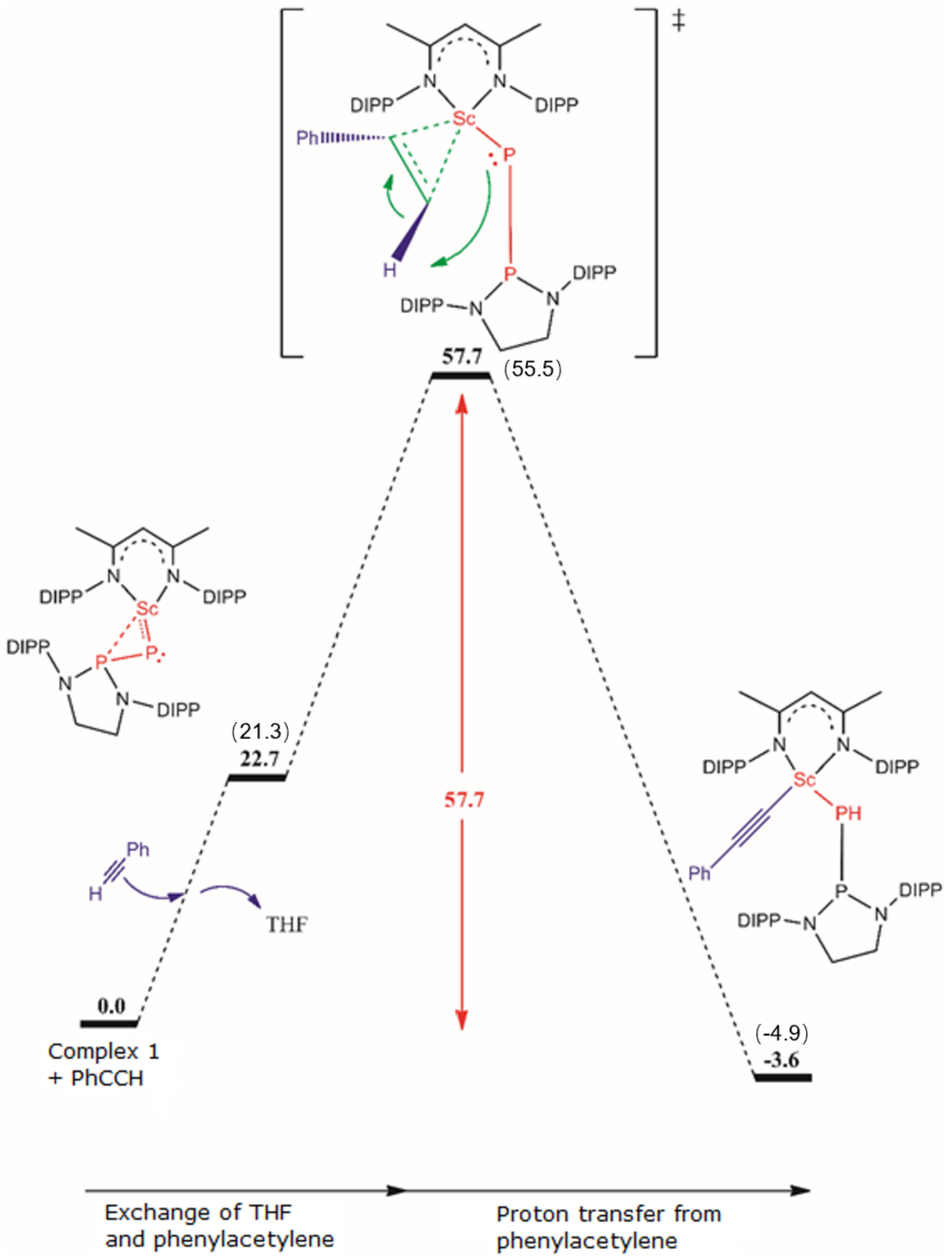

Supplementary Figure 52. Computed enthalpy pathway (in kcal mol^{-1}) for other reactivity of phenylacetylene with complex **1** (*proton transfer reaction*). The values in brackets are the Gibbs free energy.

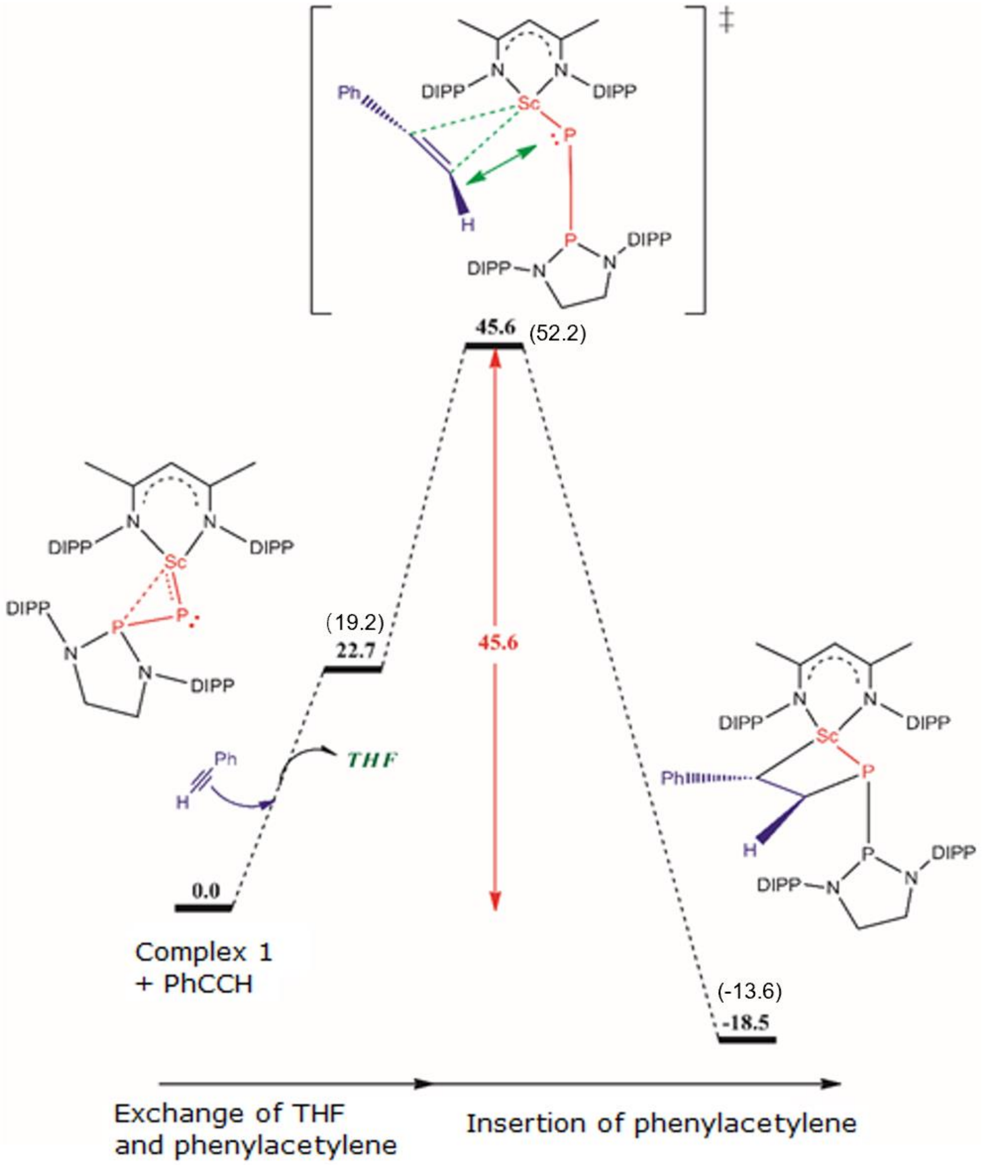

Supplementary Figure 53. Computed enthalpy pathway (in kcal mol^{-1}) for other reactivity of phenylacetylene with complex **1** (*addition to* P_{α} *atom*). The values in brackets are the Gibbs free energy.

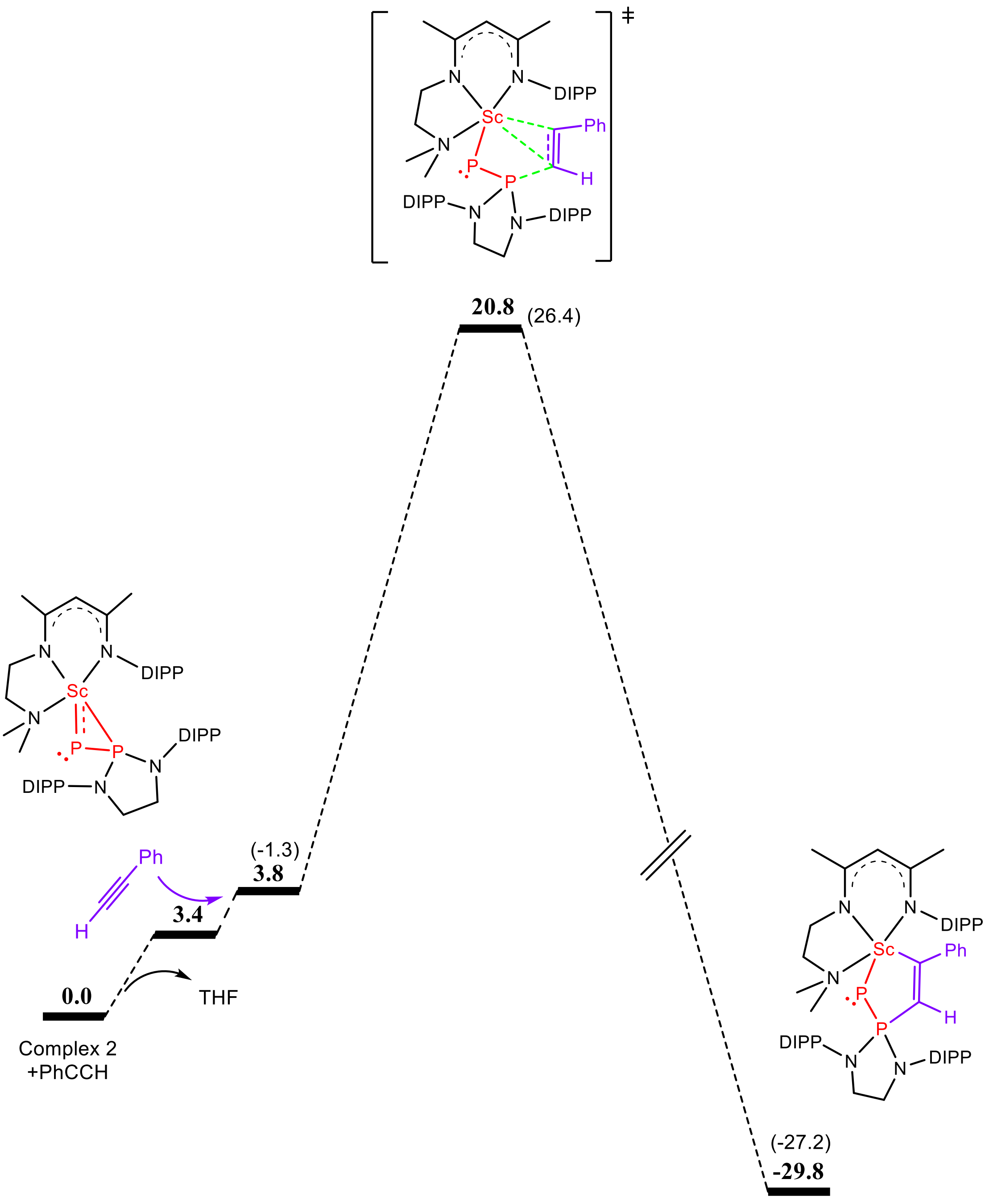

Supplementary Figure 54. Computed enthalpy pathway (in kcal mol^{-1}) for other reactivity of phenylacetylene with complex **2** (*addition to* P_β *atom*). The values in brackets are the Gibbs free energy.

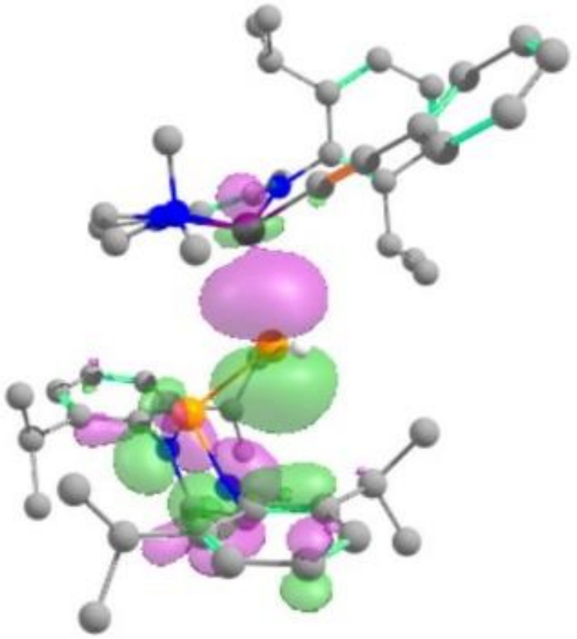

Supplementary Figure 55. HOMO-1 orbital of complex **8**. The HOMO-1 presents the interaction between the P_{α} and Sc. Atom colour code: purple, scandium; orange, phosphorus; blue, nitrogen; grey, carbon.

Supplementary References

1. Abrams, M. B., Scott, B. L. & Baker, R. T. Sterically tunable phosphenium cations: synthesis and characterization of bis(arylamino)phosphenium ions, phosphinophosphenium adducts, and the first well-defined rhodium phosphenium complexes. *Organometallics* **19**, 4944–4956 (2000).
2. Liu, L., Ruiz, D. A., Munz, D. & Bertrand, G. A singlet phosphinidene stable at room temperature. *Chem* **1**, 147–153 (2016).
3. Wildman, E. P., Balazs, G., Wooles A. J., Scheer, M. & Liddle S. T. Thorium–phosphorus triamidoamine complexes containing Th–P single- and multiple-bond interactions. *Nat. Commun.* **7**, 12884 (2016).
4. Knight, L. K., Piers, W. E., Fleurat-Lessard, P., Parvez, M. & McDonald, R. *β*-Diketiminato scandium chemistry: synthesis, characterization, and thermal behavior of primary amido alkyl derivatives. *Organometallics* **23**, 2087–2094 (2004).
5. Lv, Y. D., Zhou, J. L., Leng, X. B. & Chen, Y. F. Substitution reaction of triphenylphosphine oxide with rare-earth metal phosphido methyl complexes. *New J. Chem.* **39**, 7582–7588 (2015).
6. Mungwe, N., Swarts, A. J., Mapolie, S. F. & Westman, G. Cationic palladacycles as catalyst precursors for phenyl acetylene polymerization. *J. Organomet. Chem.* ***696***, 3527–3535 (2011).
7. Sheldrick, G. M. *SADABS*: *An Empirical Absorption Correction Program for Area Detector Data*. (University of Göttingen, Göttingen, 1996).
8. Sheldrick, G. M. *SHELXS-97* and *SHELXL-97*. (University of Göttingen, Göttingen, 1997 and 2008).
9. Sheldrick, G. M. *SHELXS-2014*. (University of Göttingen, Göttingen, 2014).
10. Dolomanov, O. V., Bourhis, L. J., Gildea, R. J., Howard, J. A. K. & Puschmann, H. OLEX2: A Complete Structure Solution, Refinement and Analysis Program. *J. Appl. Cryst.* **42**, 339−341 (2009).
11. *SMART*, Version 5.628; Bruker AXS Inc.: Madison, WI, 2002.
12. *SAINT+*, Vdrsion v7.68A; Bruker AXS Inc.: Madison, WI, 2009.
13. *SHELXTL NT/2000*, Version 6.1; Bruker AXS Inc.: Madison, WI, 2002.
14. *Gaussian 09, Revision A.02*, Frisch, M. J.; Trucks, G. W.; Schlegel, H. B.; Scuseria, G. E.; Robb, M. A.; Cheeseman, J. R.; Scalmani, G.; Barone,V.; Mennucci, B.;

Petersson, G. A.; Nakatsuji, H.; Caricato, M.; Li, X.; Hratchian, H. P.; Izmaylov, A. F.; Bloino, J.; Zheng, G.; Sonnenberg, J. L.; Hada, M.; Ehara, M.; Toyota, K.; Fukuda, R.; Hasegawa, J.; Ishida, M.; Nakajima, T.; Honda, Y.; Kitao, O.; Nakai, H.; Vreven, T.; Montgomery, J. A.; Peralta, Jr., J. E.; Ogliaro, F.; Bearpark, M.; Heyd, J. J.; Brothers, E.; Kudin, K. N.; Staroverov,V. N.; Kobayashi, R.; Normand, J.; Raghavachari, K.; Rendell, A.; Burant, J. C.; Iyengar, S. S.; Tomasi, J.; Cossi, M.; Rega, N.; Millam, J. M.; Klene, M.; Knox, J. E.; Cross, J. B.; Bakken, V.; Adamo, C.; Jaramillo, J.; Gomperts, R.; Stratmann, R. E.; Yazyev, O.; Austin, A. J.; Cammi, R.; Pomelli, C.; Ochterski, J. W.; Martin, R. L.; Morokuma, K.; Zakrzewski, V.; Voth,G.G.A.; Salvador, P.; Dannenberg, J. J.; Dapprich,S.; Daniels, A. D.; Farkas, Ö.; Foresman, J. B.; Ortiz, J. V.; Cioslowski, J.; Fox, D. J. Gaussian, Inc., Wallingford CT, 2009

15. Becke, A. D. Density-functional thermochemistry. III. The role of exact exchange *J. Chem. Phys.* **98**, 5648 (1993).
16. Perdew, J. P. & Wang, Y. Accurate and Simple analytic representation of the electron-gas correlation energy. *Phys. Rev. B* **45**, 13244−13249 (1992).
17. Burke, K., Perdew, J. P. & Yang, W. *Electronic Density Functional Theory: Recent Progress and New Directions*, ed. J. F. Dobson, G. Vignale, M. P. Das, Plenum, New York, 1998.
18. Bergner, A., Dolg, M., Kuechle, W., Stoll, H. & Preuss, H. Ab initio energy-adjusted pseudopotentials for elements of groups 13–17. *Mol. Phys.* **1993**, *80*, 1431−1441.
19. Leininger, T. et al. Spin-orbit interaction in heavy group 13 atoms and T1Ar. *J. Chem. Phys*. **217**, 19−27 (1997).
20. Ditchfield, R., Hehre, W. J. & Pople, J. A. Self-consistent molecular-orbital methods. IX. An extended Gaussian-type basis for molecular-orbital studies of organic molecules *J. Chem. Phys.* **54**, 724−728 (1971).
21. *NBO* Version 3.1, Glendening, E. D., Reed, A. E., Carpenter, J. E. & Weinhold, F.
22. Lu, T. & Chen, F. Multiwfn: A multifunctional wavefunction Analyser, *J. Comput. Chem.* **33**, 580−592 (2012).
