# Supplementary material for: Synthesis and versatile reactivity of scandium phosphinophosphinidene complexes
Source: Nat Commun. 2020 Jun 9;11:2916. doi: 10.1038/s41467-020-16773-w (PMC7283324; doi:10.1038/s41467-020-16773-w)
Supplement: Supplementary file 3 — Description of Additional Supplementary Files [file 41467_2020_16773_MOESM3_ESM.pdf]

Description of Additional Supplementary Files

File Name: Supplementary Data 1
Description: The datasets of Cartesian coordinates in the format of txt
